# Cost-effectiveness of pirfenidone for patients with cancer and grade 2 or grade 3 radiation-induced lung injury: A modeling study

**DOI:** 10.1371/journal.pmed.1004817

**Published:** 2026-08-05

**Authors:** Qiu-Yi Pan, Ya-Xue Wang, Xue-Ying Tang, Zan Hou, Bai-Qiang Dong, Qi-Wen Li, Bao-Qing Chen, Shou-Min Bai, Chen-Fei Wu, Ming Chen

**Affiliations:** 1 Sun Yat-sen University Cancer Center, State Key Laboratory of Oncology in South China, Department of Radiation Oncology, Collaborative Innovation Center of Cancer Medicine, Guangzhou, P. R. China; 2 United Laboratory of Frontier Radiotherapy Technology of Sun Yat-sen University & Chinese Academy of Sciences Ion Medical Technology Co, Ltd, Guangzhou, P. R. China; 3 Department of Radiation Oncology, Sun Yat-sen Memorial Hospital, Sun Yat-Sen University, Guangzhou, P. R. China; 4 Department of Radiation Oncology, Sichuan Clinical Research Center for Cancer, Sichuan Cancer Hospital & Institute, Sichuan Cancer Center, Affiliated Cancer Hospital of University of Electronic Science and Technology of China, Chengdu, Sichuan, China; Washington University in St Louis, UNITED STATES OF AMERICA

## Abstract

**Background:**

The cost-effective treatment strategy for radiation-induced lung injury (RILI) remains unclear. Our recent phase II randomized clinical trial has proved pirfenidone to be safe and effective for grade 2 or 3 RILI. Here, we aim to assess the cost-effectiveness of pirfenidone plus glucocorticoids versus glucocorticoids alone for the treatment of grade 2 or 3 RILI.

**Methods and findings:**

We constructed a Markov model based on the individual-level data from our trial (NCT03902509) to evaluate the cost-effectiveness of pirfenidone plus glucocorticoids. Outcomes were simulated in a 4-week cycle over a 24-week time horizon to model patients who had acute pulmonary exacerbation or RILI progression. Costs and utilities were discounted at 3% annually. Health outcomes were measured using incremental cost-effectiveness ratio (ICER), representing incremental costs per quality-adjusted life year (QALY) gained between the two treatment groups. One-way and probabilistic sensitivity analyses were conducted to evaluate the model uncertainties. The simulated RILI pattern in the Markov model closely matched the pattern in the trial cohort, indicating that the model accurately described the disease process of RILI. Compared with glucocorticoids alone, pirfenidone plus glucocorticoids yielded an additional 0.028 QALYs at an incremental cost of $590, with an ICER of $20,956 per QALY gained. The ICER was considerably below the commonly accepted willingness-to-pay (WTP) threshold of $100,000 per QALY. In one-way sensitivity analyses, the variables with the greatest influence on the ICER included the acute pulmonary exacerbation-free survival and the probability of non-response among patients with grade 2 or 3 RILI in glucocorticoid group. Moreover, the probabilistic sensitivity analysis demonstrated that pirfenidone plus glucocorticoids had a 99.98% probability of being cost-effective at the WTP threshold of $100,000/QALY. Limitations include the phase II trial data with limited sample size and follow-up. An ongoing phase III trial will provide further evidence.

**Conclusions:**

For grade 2 or 3 RILI, pirfenidone plus glucocorticoids is cost-effective compared with glucocorticoids alone. This strategy offers a practical and evidence-based option for RILI clinical management.

## Author summary

**Why was this study done?:**

- RILI is a common and serious complication in patients with cancer receiving thoracic radiotherapy, while current treatment strategies primarily based on glucocorticoids remain limited in effectiveness.
- Pirfenidone plus glucocorticoids has demonstrated favorable efficacy and safety in a large-scaled randomized controlled trial involving patients with grade 2 or 3 RILI, but its cost-effectiveness remains unknown.
- Understanding the economic impact of adding pirfenidone to standard glucocorticoid therapy for grade 2 or 3 RILI could help inform whether pirfenidone regimen improves health outcomes in a cost-effective manner.

**What did the researchers do and find?:**

- We developed a well-calibrated Markov model based on patient-level data from our randomized clinical trial to compare pirfenidone plus glucocorticoids with glucocorticoids alone in patients with grade 2 or 3 RILI.
- The pirfenidone regimen was cost-effective for patients with grade2 or 3 RILI and showed greater cost-effectiveness among patients with a poor response to standard glucocorticoid therapy.
- The pirfenidone regimen was likely to remain cost-effective across a relatively wide range of medication costs, as well as a broad spectrum of tumor types and tumor burden.

**What do these findings mean?:**

- Pirfenidone plus glucocorticoids could improve health outcomes in cancer patients with grade 2 or 3 RILI while representing a reasonable value for money.
- These results support the potential role of pirfenidone in the management of grade 2 or 3 RILI and may inform healthcare providers and policymakers in resource allocation decisions.
- A limitation of our study is that model parameters are based on a phase II trial. Further phase III trial is warranted to confirm the long-term clinical and economic benefits of pirfenidone in patients with RILI.

## Abstract 3

In this modelling study, Qiu-Yi Pan and colleagues provide a cost-effectiveness analysis of pirfenidone plus glucocorticoids versus glucocorticoids alone for the treatment of Grade 2 and Grade 3 Radiation-Induced Lung Injury in patients with cancer.

## Introduction

Radiation-induced lung injury (RILI), characterized by early chronic inflammation and late-stage fibrosis, ranks among the gravest complications of thoracic radiotherapy [1]. Approximately 20% of patients receiving thoracic radiotherapy exhibit RILI of variable severity [2], resulting in irreversible lung deterioration with reduced respiratory status and quality of life (QoL) [3,4]. Currently, standard treatment of RILI is primarily based on glucocorticoids [5]. However, over 22% of the patients receiving glucocorticoids experience recurrence after glucocorticoids tapering [6], leading to prolonged therapy and thus aggravated treatment-related toxicity [7,8]. In addition, the long-term antifibrotic efficacy of glucocorticoids remains unvalidated [5]. These limitations collectively impair sustained pulmonary function improvement.

To date, no validated alternative overcomes the limitations of glucocorticoids for durable recovery of pulmonary function. Trials of amifostine, nintedanib, and pentoxifylline have yielded limited evidence owing to restricted sample sizes, inconsistent efficacy results, or toxicity-related constraints [9–13]. Notably, our recent phase II randomized controlled trial (RCT, NCT03902509) reported Pirfenidone [14], an orally bioavailable pyridone analog with anti-inflammatory, anti-fibrotic, and antioxidant properties [15,16], to be effective and safe for the treatment of RILI. Based on multi-center data from 134 patients with grade 2 or 3 RILI, pirfenidone achieved durable improvement in pulmonary function relative to standard glucocorticoid therapy. Specifically, pirfenidone alleviated inflammation, fibrosis, and acute exacerbations, without amplifying treatment-related toxicity.

Fundamentally, efficacy data derived solely from RCTs are inadequate to support the update of therapeutic regimens [17]. Current value-based oncology care necessitates a nuanced appraisal of therapies integrating both clinical value, QoL and economic feasibility [18]. Despite pirfenidone’s pronounced efficacy in restoring pulmonary function, the long-term outcome of RILI is independently linked to health-related quality of life (HRQoL), such as poor physical function and severe respiratory symptom [19]. Therefore, clinical endpoints alone may inadequately capture the individual-level therapeutic benefit. Moreover, pirfenidone’s steep cost compared with glucocorticoids challenges broad accessibility and clinical use. Potential additional costs like time costs, geographic resource heterogeneity, and variability in benefit patterns, further complicate the process of weighing the benefits and costs for pirfenidone [20].

Accordingly, a rigorous evaluation on pirfenidone’s clinical value is warranted to expand its application and inform healthcare policy. Cost-effectiveness analysis enables quantification of the relative benefits and costs among alternative interventions in a consistent framework. Therefore, this study aims to assess the cost-effectiveness of pirfenidone plus glucocorticoids versus glucocorticoids alone in patients with grade 2 or 3 RILI.

## Methods

### Model construction

Previous studies have suggested that the Markov model can dynamically integrate patient distributions across multiple health states, along with the frequency of clinical events and their associated costs and health utility changes. It also allows the simulation of the stochastic nature of real-world clinical events and supports multi-level sensitivity analyses to enhance external validity, making them particularly suitable for evaluating the cost-effectiveness of pirfenidone in real-world clinical settings. Accordingly, we constructed a Markov decision-analytic model using individual-level data from the phase II RCT to evaluate the cost-effectiveness of pirfenidone plus glucocorticoids versus standard glucocorticoid therapy in patients with grade 2 or 3 RILI. Considering that pirfenidone and glucocorticoids primarily exert anti-inflammatory effects, our analysis focused on the acute radiation pneumonitis phase of RILI occurring within the 24-week following radiotherapy [1,21]. This approach not only aligns with the follow-up period of our RCT, ensuring the availability and reliability of model input parameters, but also avoids confounding from subsequent antifibrotic effects on health outcomes between the two treatment groups. Therefore, we defined the health states of RILI in the Markov model using different RP grades based on the Common Terminology Criteria for Adverse Events (CTCAE v5.0) following our RCT [22]. Of note, since only 9.7% of RCT-enrolled patients presented with grade 3 RP. To guarantee the statistical robustness of the model parameters, we combined grades 2 and 3 into a single health state in accordance with our phase II trial design. In addition, acute pulmonary exacerbation (APE) is a key clinical outcome in our RCT that reflects pulmonary function impairments and impacts overall survival [23,24]. We incorporated it into the model as a complementary indicator of pirfenidone’s anti-inflammatory efficacy. Accordingly, the model includes the following mutually exclusive health states: No RP, RP (grade 1, grade 2 or 3, grade 4), APE, No APE, and death.

**Fig 1:**
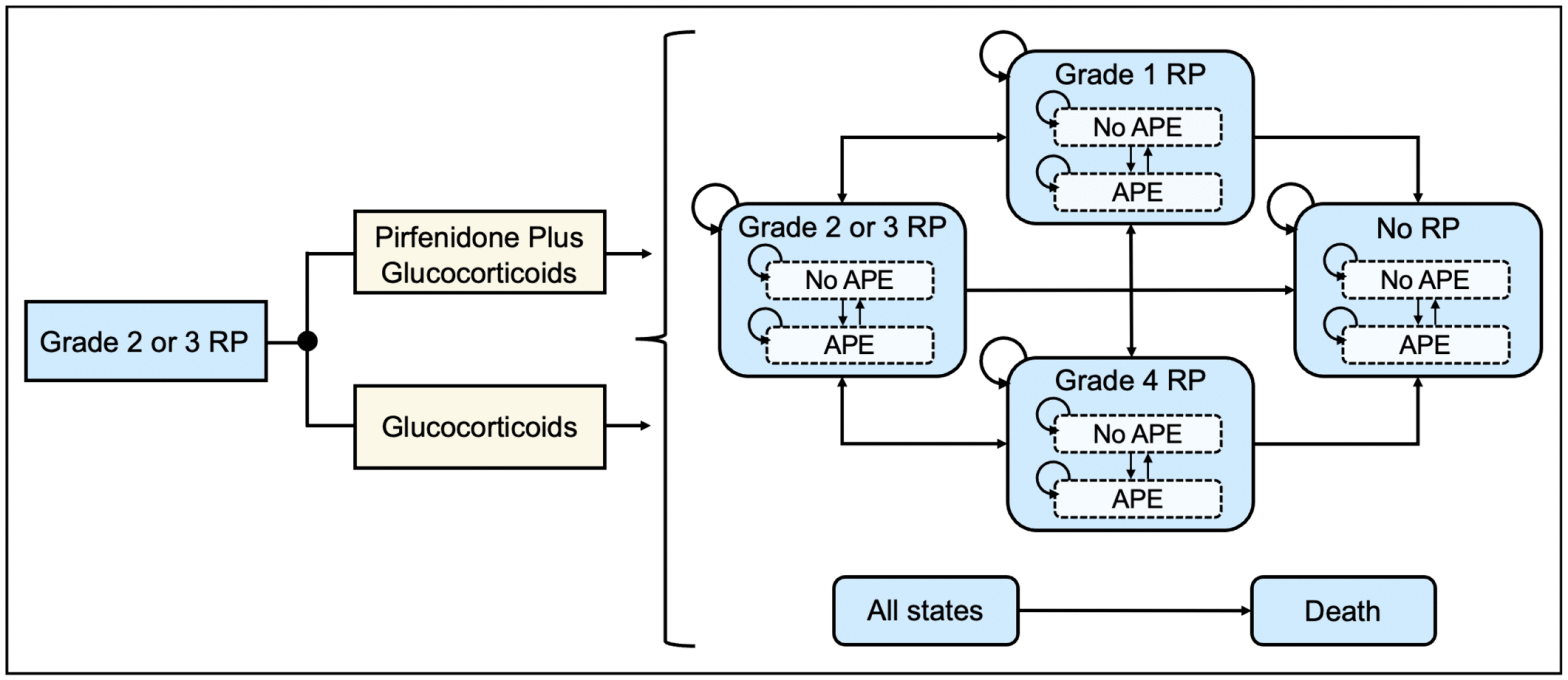
Diagram of Markov model. Patients with grade 2 or 3 RP treated with either pirfenidone plus glucocorticoids or glucocorticoids would enter the Markov model, where patients move between the health states based on prespecified 4-week transition probabilities. During the treatment period, patients may transition between RP states and are at risk of APE. Abbreviations: RP, radiation pneumonitis; APE, acute pulmonary exacerbation.

A simplified diagram of the Markov model is shown in Fig 1. The simulated cohort enters the model in the grade 2 or 3 RP state at the first cycle and then transitions to other states based on predefined transition probabilities [25], accruing assigned costs and utilities. All costs and utilities were discounted at an annual rate of 3% (Table A in S1 Appendix) [26–28]. Detailed parameter assignment methods, including transition probabilities, costs, and utility values, are provided in S2 Appendix. Following the surveillance schedule of our phase II RCT, the model cycle length was set at 4 weeks, and the model ran for 6 cycles to align with the acute phase of RILI [21].

Primary outcomes were cost, effectiveness, incremental cost-effectiveness ratio (ICER), and net health benefit (NHB). The ICER was defined as the additional cost per quality-adjusted life year (QALY) gained. A willingness-to-pay (WTP) threshold of $100,000/QALY was applied, with strategies below this threshold considered cost-effective [29]. The NHB, a unitary measure capturing population health gain, was calculated as NHB = effectiveness − (cost/WTP) [30]. The strategy with the highest NHB at the given WTP threshold was deemed the most cost-effective [31].

All analyses were conducted in accordance with the Consolidated Health Economic Evaluation Reporting Standards (CHEERS 2022) (Table B in S1 Appendix) [32]. As this analysis used publicly available data and de-identified summary results from the corresponding RCT, the institutional ethics committee of Sun Yat-sen University Cancer Center approved the study (SL-B2021-336-02). The Markov model was developed in TreeAge Pro 2022 (TreeAge Software, Williamstown, MA), and additional statistical analyses were performed using R software (version 3.6.1, http://www.r-project.org). All statistical tests were two-sided, with *P* values < 0.05 indicating statistical significance.

### Model validation

Since the model time horizon was aligned with our RCT duration, real-world individual-level data were available for robust validation of the model. To guarantee an accurate and realistic simulation of the progression of RILI, we performed a two-level validation. First, we compared the model-simulated 24-week acute pulmonary exacerbation-free survival (APEFS) against the APEFS from the RCT cohort. Model-simulated APEFS was generated from the Monte Carlo simulation cohort, while the real-world APEFS was directly derived from the RCT individual-level data. Second, the model-simulated distribution of RP severity (including no RP, grade 1, grade 2 or 3, and grade 4) at four surveillance time points was compared against observed data in our RCT cohort using Chi-squared tests.

### Costs

Unit costs were derived from either the 2025 Medical Insurance Administration Bureau of Guangzhou, China, or published studies from the societal perspective. All cost inputs are provided in Table A in S1 Appendix, and detailed treatment and follow-up regimens are available in S2 Appendix. Direct medical costs included pirfenidone, glucocorticoids, medications for infection [33] and respiratory symptom relief (e.g., antitussives, expectorants, bronchodilators) [34], respiratory support [35], routine imaging and laboratory testing, and additional expenditures associated with APE events [36]. All treatment and surveillance protocols for RILI were consistent with the international guidelines on radiation-induced lung injury [37,38], and APE management was based on the 2022 ATS/ERS/JRS/ALAT Clinical Practice Guideline for Idiopathic Pulmonary Fibrosis [39]. According to the official methodological guidelines of the International Society for Pharmacoeconomics and Outcomes Research (ISPOR) regarding the definition of a “societal perspective” [40,41], this study comprehensively considered multiple relevant societal costs. For outpatients, transportation, accommodation, and additional meal expenses during follow-up were included [42,43], whereas for patients hospitalized due to severe RILI or APE, wage loss and additional nutrition costs were additionally accounted for [43,44]. All costs mentioned above were converted to U.S. dollars using the exchange rate on Aug 5, 2025 (1 USD = CNY 7.2011).

### Utility estimates

QoL utility estimates for different health states were expressed in quality-adjusted life-years (QALYs) (Table A in S1 Appendix) [45]. In accordance with the phase II RCT inclusion criteria, baseline health utility was assigned based on the utility values of patients responding to thoracic radiotherapy [46]. Utility decrements due to RP were informed by published data on infectious pneumonitis and RP [47,48]. Disutility values for mild and severe APE were derived from previous literature based on respiratory symptom type and severity [46,49]. Treatment-related toxicity was not incorporated in the model, given the comparable incidence of grade 3 or higher adverse events between the pirfenidone and glucocorticoid groups in our RCT. Details regarding the assignment of utility values are provided in S2 Appendix.

### Sensitivity analysis

To evaluate the robustness of the results to parameters’ uncertainty, we conducted extensive sensitivity analyses. One-way sensitivity analyses were performed by varying individual parameters within ranges derived from published literature or applying a deviation of 20% from the base-case values. In addition, two-way sensitivity analyses were conducted for key parameters of interest (Table A in S1 Appendix). Probabilistic sensitivity analyses (PSA) were conducted using 10,000 Monte Carlo simulations, with all parameters simultaneously sampled from predefined probability distributions: beta for probabilities and utilities, gamma for costs, and uniform for the discount factor. PSA results were illustrated using the incremental cost-effectiveness scatter plot and the cost-effectiveness acceptability curve.

### Use of artificial intelligence tools

ChatGPT was used for grammar verification and phrasing refinement. The authors thoroughly reviewed and edited the text, taking full responsibility for its accuracy and quality.

## Results

### Model validation

The APEFS rates generated from the Markov model-simulated cohort via Monte Carlo simulations closely matched the RCT observations for both the pirfenidone (*P* = 0.9906) group and the control (*P* = 0.9707) group (Fig A in S1 Appendix). The distribution of RP severity in the Markov model-simulated cohort aligned well with the observed data in the RCT cohort across all surveillance time points (Table C in S1 Appendix). Overall, these validation results confirm the model’s capability to accurately simulate real-world RILI disease progression under both treatment strategies.

### Base-case analysis

In the base-case scenario, patients receiving pirfenidone plus glucocorticoids accrued 0.270 QALYs, compared with 0.242 QALYs in the control group of glucocorticoids alone. Total costs were $5,029 and $4,438 for the pirfenidone and control groups, respectively. Compared with glucocorticoids alone, pirfenidone plus glucocorticoids resulted in an incremental cost of $590 for an additional 0.028 QALYs, yielding an ICER of $20,956 per QALY (Table 1). This ICER is well below the commonly accepted WTP threshold (i.e., $100,000 per QALY), indicating that a 24-week course of pirfenidone plus glucocorticoids is highly cost-effective for patients with grade 2 or 3 RILI. Furthermore, the pirfenidone regimen yielded an incremental NHB of 0.022 QALY than glucocorticoids alone, with values of 0.220 QALY and 0.198 QALY, respectively.

**Table 1:** Base-case cost-effectiveness analysis.

Treatment strategy	Total values		Incremental values		ICER ($/QALY)	NHB (QALY)
	Cost ($)	Effectiveness (QALY)	Cost ($)	Effectiveness (QALY)		
Glucocorticoids	4,438	0.242	——	——	——	0.198
Pirfenidone plus glucocorticoids	5,029	0.270	590	0.028	20,956	0.220

Abbreviations: ICER, incremental cost-effectiveness ratio; QALY, quality-adjusted life-year; NHB, net health benefit.

### Sensitivity analysis

**Fig 2:**
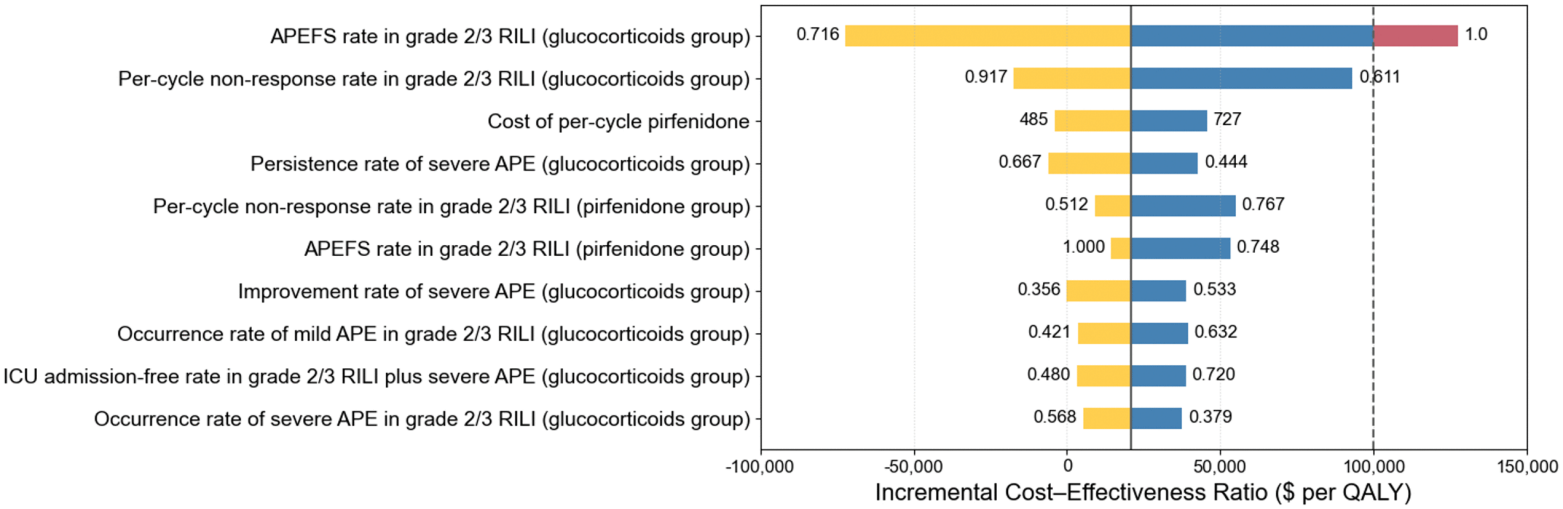
Tornado diagram of one-way sensitivity analysis. The top 10 parameters contributing most to uncertainty in each comparison are shown. The figure depicts the influence of parameter variation on the ICERs between pirfenidone plus glucocorticoids and glucocorticoid alone in patients with grade 2 or 3 RILI. Blue bars and yellow bars illustrate the ICERs that are greater and less than the base-case ICERs, respectively. Red bars indicate that the ICERs go across the WTP threshold, leading to a switch of the most cost-effective strategy. Numbers on either side of the bars indicate the range of values used for each parameter in the sensitivity analysis. Solid and dashed lines represent the ICERs in the base-case analysis and the WTP threshold of $100,000 per QALY, respectively. Abbreviations: APEFS, acute pulmonary exacerbation free survival; RILI, radiation-induced lung injury; APE, acute pulmonary exacerbation; ICU, intensive care unit; WTP, willingness-to-pay; QALY, quality-adjusted life-year.

To assess the robustness of the model, we conducted sensitivity analyses across all transition probabilities, utilities, and cost parameters (Table A and Fig B in S1 Appendix). One-way sensitivity analysis illustrated that our model remained robust across most scenarios (Fig 2). The ICER was most sensitive to clinical outcomes among patients with grade 2 or 3 RILI, especially the APEFS rate in the glucocorticoid group. When the APEFS rate varied from 71.6% to 100%, the ICER increased from −$72,155 to $127,505 per QALY. Notably, when the APEFS rate of glucocorticoid group exceeded 97.8%, standard glucocorticoid therapy emerged as the preferred strategy due to the high cost of pirfenidone; conversely, when the rate dropped below 86.4%, pirfenidone was dominant, defined as superior clinical effectiveness with less economic requirements [50]. For all other parameters, ICERs remained below the WTP threshold of $100,000 per QALY, peaking at $93,189 when the per-cycle non-response rate in the glucocorticoid group decreased to 61.1%. Pirfenidone became dominant when the rate surpassed 83.8%. These results further confirm the robustness of pirfenidone’s cost-effectiveness across various clinical scenarios.

It’s worth noting that previous large-scale studies reported a grade 4 RP incidence of less than 1.2% [51–57], and no grade 4 cases were observed in our RCT cohort. Therefore, grade 4 RP-related transition probabilities were estimated by linear extrapolation from lower-grade probabilities. We spanned the probability of grade 0, 1, 2 or 3 RP progressing to grade 4 between 0% and 1.2% in one-way sensitivity analyses. In the pirfenidone group, the ICER was most sensitive to the probability of grade 2 or 3 RP worsening to grade 4, increasing from $20,956 to $28,214 per QALY as this probability rose from 0% to 1.2%. The least influential parameter was the probability of grade 0 RP progressing to grade 4, which increased the ICER only slightly, from $20,956 to $22,694 per QALY. In the glucocorticoids group, the ICER was likewise most sensitive to the probability of grade 2 or 3 RP worsening to grade 4, decreasing from $20,956 to $2,522 per QALY as the probability rose from 0% to 1.2%. In comparison, the probability of grade 0 RP progressing to grade 4 lowered the ICER from $20,956 to $20,178 per QALY. Overall, these results demonstrated that grade 4 RP-related transition probabilities exert a minor impact on the cost-effectiveness of pirfenidone. Given that the real-world incidence of grade 4 RP is typically below 1.2%, its effect on model robustness can be considered negligible.

**Fig 3:**
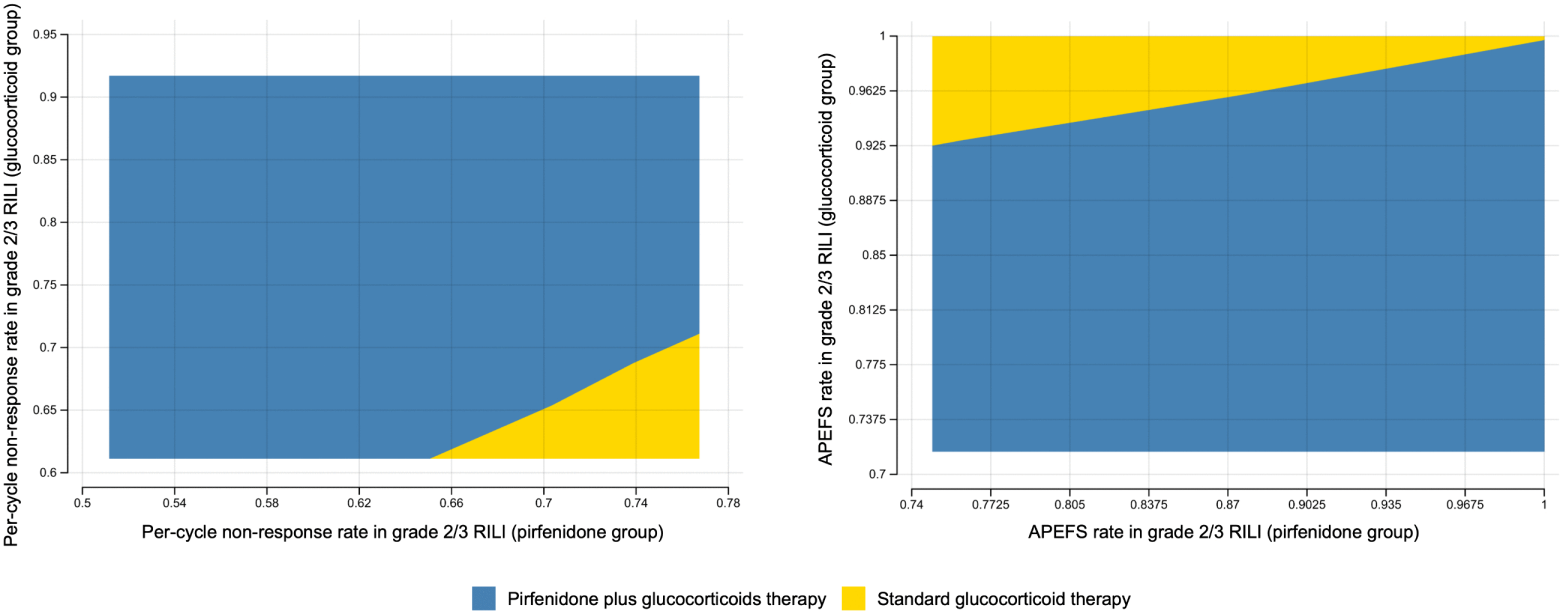
The two-way sensitivity analyses of the Per-cycle non-response rate (left) and the APEFS rate (right) in patients with grade 2 or 3 RILI. The blue area indicates parameter combinations for which pirfenidone plus glucocorticoids is the more cost-effective strategy, whereas the yellow area indicates combinations for which standard glucocorticoid therapy is the more cost-effective strategy. Abbreviations: RILI, radiation-induced lung injury; APEFS, acute pulmonary exacerbation-free survival.

In the two-way sensitivity analysis, we focused on the impact of simultaneous variations in corresponding disease progression parameters between the pirfenidone plus glucocorticoids strategy and the glucocorticoid strategy on the cost-effectiveness conclusions (Fig C in S1 Appendix). The results indicated that, at a WTP threshold of $100,000 per QALY, pirfenidone plus glucocorticoids remained the preferred strategy in the majority of scenarios. Fig 3 highlights the parameter combinations that could potentially influence cost-effectiveness, specifically when the per-cycle non-response rates and APEFS rates of patients with grade 2 or 3 RILI in both treatment arms varied simultaneously. Notably, only under the extreme condition where the APEFS rate in the glucocorticoids group exceeds 99.76% would pirfenidone plus glucocorticoids fail to be cost-effective, regardless of the APEFS rate in the pirfenidone group.

**Fig 4:**
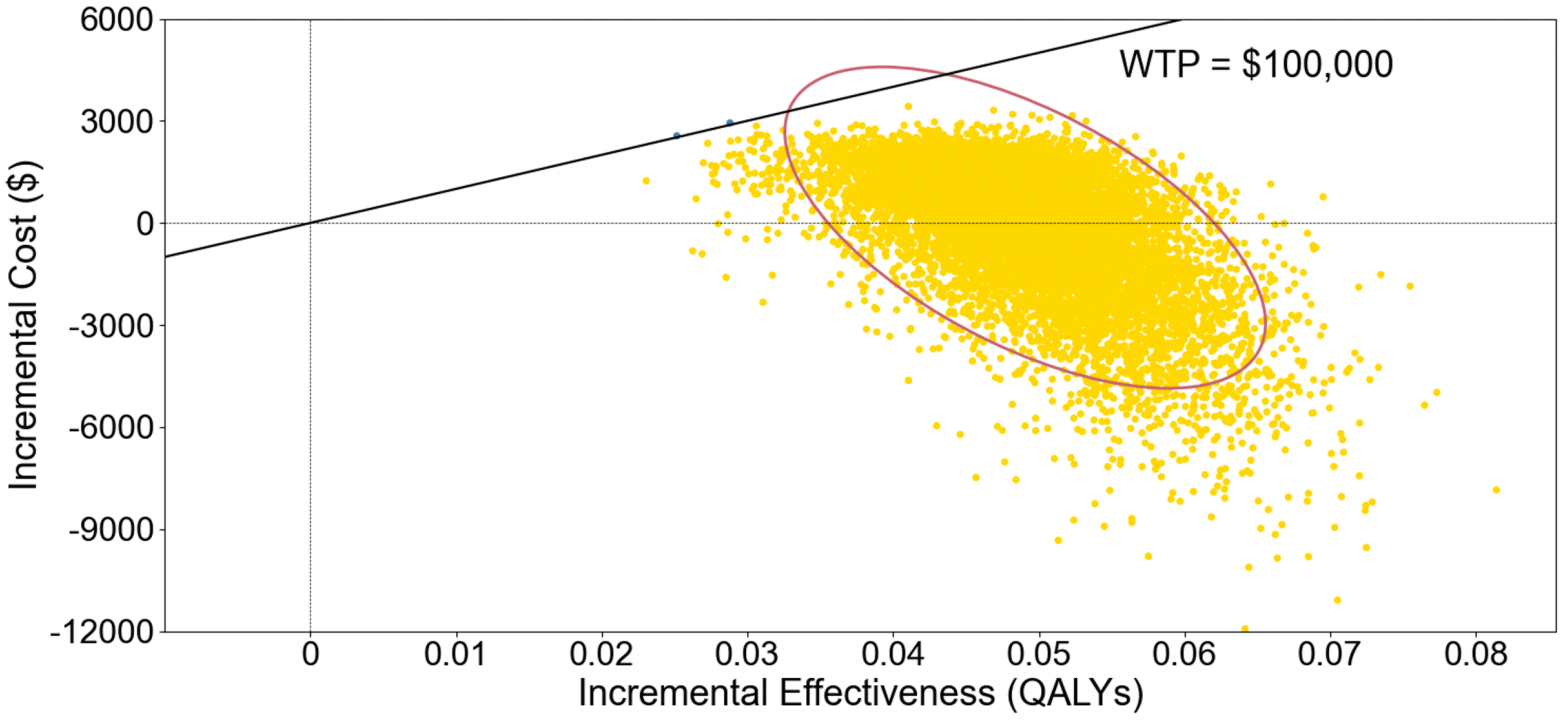
Scatter plot of probabilistic sensitivity analysis. Black line indicates threshold of WTP of $100,000 per QALY. Each point in scatterplot corresponds to one sample of parameter values. Points above reference line indicate higher incremental cost effectiveness ratio than threshold value. The red ellipse represents the 95% confidence ellipse around the mean incremental cost and incremental effectiveness. Abbreviations: QALY, quality-adjusted life-year; WTP, willingness-to-pay.

**Fig 5:**
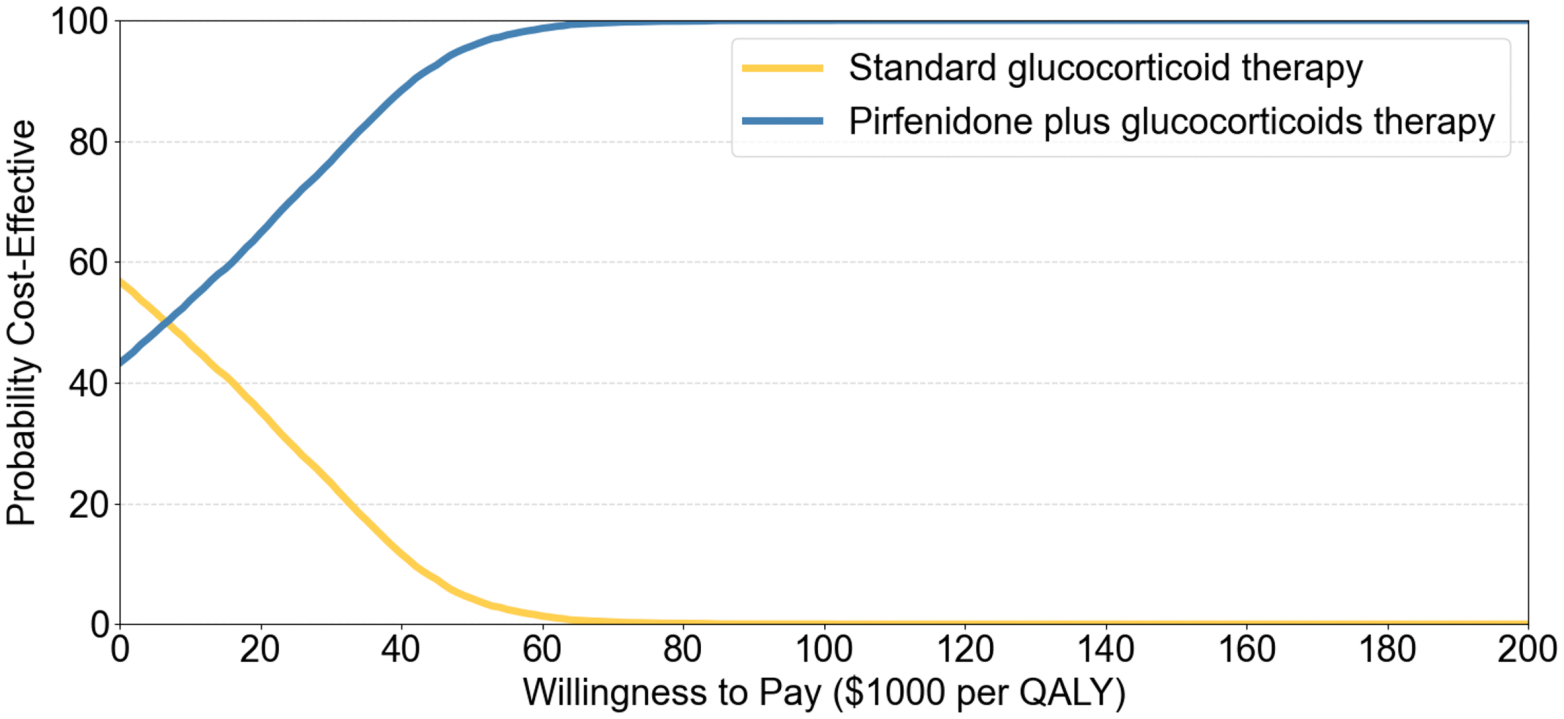
Cost-effectiveness acceptability curves. This figure illustrates the acceptability of each RILI treatment strategy in the probabilistic sensitivity analysis. The curves in different colors illustrate the probability of each treatment strategy being the most cost effective at a given WTP threshold. Abbreviations: QALY, quality-adjusted life-year; WTP, willingness-to-pay.

PSA results from 10,000 Monte Carlo simulations are presented in a cost-effectiveness scatter plot, where quadrants represent differences in cost and effectiveness (Fig 4). Across the Monte Carlo iterations, 56.74% of simulated outcomes were located in the northeast quadrant (cost-effective strategy, defined as more effective and more costly with ICERs below the WTP threshold), whereas 43.24% were in the southeast quadrant (dominant strategy, defined as more effective and less costly). Overall, pirfenidone plus corticosteroids had a cost-effectiveness probability of 99.98% at a WTP threshold of $100,000 per QALY, underscoring its economic advantage over glucocorticoids alone. Furthermore, the cost-effectiveness acceptability curve illustrated that as the WTP threshold increased, the likelihood of the pirfenidone strategy being cost-effective steadily increased, while that of the control strategy declined (Fig 5). At WTP thresholds above $6,681 per QALY, pirfenidone plus glucocorticoids had a higher probability of being cost-effective than glucocorticoids alone.

### Clinical scenario analysis

Specific clinical uncertainties can be further explored in sensitivity analyses. Although our RCT excluded patients with confirmed tumor progression, baseline tumor burden of the patients in real-world clinical practice varied considerably. Specifically, we selected a base-case health utility value of 0.712, which represented patients with non-small cell lung cancer (NSCLC) after treatment since it was the most prevalent subgroup (56.9%) of enrolled patients in our RCT (Table D in S1 Appendix) [46]. Notably, due to the current lack of large-sample, population-specific utility data for Chinese patients, we were compelled to use health utility values derived from White populations as the base-case value. Considering potential influences such as racial differences and health preferences, we applied a relatively wide parameter range of 0.600 to 0.981 in our sensitivity analyses. The chosen range was determined based on the assumption that the health utility value for patients without tumor progression should be lower than that of the general population but higher than that of patients with progressive disease [58]. It was also aimed to comprehensively represent various cancer types and treatment responses in clinical practice, encompassing patients with lung, esophageal, and breast cancer across disease states from progressive disease to complete response, respectively. Data indicate that among these thoracic malignancies, patients with small cell lung cancer (SCLC) in progressive disease have the lowest utility, whereas patients achieving complete response reach utility values approaching those of a tumor-free general population [59]. Accordingly, the lower bound of 0.600 was derived from the studies reporting utility values for patients with progressive small cell lung cancer (SCLC) [59]. This value was selected because it is lower than the reported utility values for patients with progressive NSCLC, esophageal cancer, and breast cancer [60,61], thereby allowing the analysis to cover a broader and more convincing range of potential variation. The upper bound of 0.981 was obtained from studies reporting health utility values for the general population in China [62–64]. Sensitivity analyses indicated that the ICER decreased from $21,198 per QALY as baseline health utility increased from 0.60, remaining well below the WTP threshold of $100,000 per QALY. These findings suggest that the cost-effectiveness of pirfenidone is robust across patients with various degrees of tumor burden, providing additional evidence supporting its widespread clinical use.

Due to the RCT design, grade 2 and grade 3 RILI were combined into a single health state in the base-case analysis. However, given their substantial differences in treatment costs and health utility decrements, we performed further validation of the relevant parameters. On the one hand, we estimated per-cycle costs for grade 2 and grade 3 RILI separately based on clinical guidelines (S2 Appendix), and calculated a weighted cost for the combined “grade 2 or 3 RILI” health state using actual proportions of patients with grade 2 and grade 3 from our RCT (Table A in S1 Appendix). Further one-way sensitivity analyses simulating variations in grade 3 RILI incidence showed that when the proportion of grade 3 RILI in the pirfenidone group varied between 1.9% and 2.9%, the ICER ranged from $20,534 to $21,378 per QALY. When the proportion in the glucocorticoids group varied between 3.6% and 5.4%, the ICER decreased from $22,252 to $19,661 per QALY. Notably, pirfenidone would lose cost-effectiveness only if its grade 3 RILI proportion reached 93.2%, far exceeding real-world incidence [65]. On the other hand, we performed one-way sensitivity analyses on the utility decrement of the combined “grade 2 or 3 RILI” health state. Results showed that when this utility decrement varied between 0.024 and 0.032, the ICER decreased from $24,144 to $18,512 per QALY. Notably, the highest ICER of $61,659 per QALY was observed when this utility decrement was set to 0. Taken together, differences in treatment costs and utility decrements between grade 2 and grade 3 RILI are unlikely to materially reverse our cost-effectiveness conclusions.

Adverse events (AE) represent an essential dimension when evaluating treatment strategies. In our base-case analysis, we incorporated no AE-related parameters into the Markov model considering the comparable incidence of severe AEs between the pirfenidone and control groups (11% versus 10%). However, to fully assess the potential impact of pirfenidone-related AEs on the robustness of our cost-effectiveness conclusions, we supplemented the origin model with parameters for pirfenidone-related AEs (Table A in S1 Appendix; S2 Appendix) [66,67]. Under the clinical scenario, the pirfenidone plus glucocorticoids regimen yield an additional 0.027 QALYs at an incremental cost of $609, resulting in an ICER of $22,715 per QALY (Table E in S1 Appendix). One-way sensitivity analyses further demonstrated that pirfenidone regimen consistently remained cost-effective across a ±20% variation in these parameters. Specifically, the highest ICER for pirfenidone of $22,943 per QALY occurred when the per-cycle incidence of grade 3–4 adverse events reached 2.0%. PSA similarly confirmed the robustness of our conclusions: pirfenidone remained cost-effective in approximately 99.94% of simulations, with 56.87% of simulated samples falling in the northeast quadrant and 43.13% in the southeast quadrant (Fig D in S1 Appendix). Taken together, plausible variations in the incidence and severity of pirfenidone-related AEs in real-world clinical practice are unlikely to reverse our cost-effectiveness conclusions.

Given that the WTP threshold is closely related to the local economic context, income level, reimbursement system, and healthcare affordability, the internationally accepted threshold of $100,000 per QALY may not fully represent the cost-effectiveness results of an RCT conducted in the Chinese healthcare context. According to the updated China guidelines for pharmacoeconomic evaluations [68], we selected twice the gross domestic product (GDP) per capita as the WTP threshold for each QALY gained, which corresponds to $27,680 per QALY and better reflects the local decision-making context. Notably, the base-case ICER for the pirfenidone regimen was $20,956 per QALY, falling below this China-specific WTP threshold, indicating favorable cost-effectiveness. Furthermore, the PSA based on 10,000 Monte Carlo simulations reinforced the findings, showing that 73.93% of the simulated samples had an ICER below the WTP threshold, with 43.24% representing a dominant strategy (Fig E in S1 Appendix). Overall, the pirfenidone regimen maintained a clear economic advantage over glucocorticoids alone under this locally-relevant threshold.

## Discussion

Our study provides a cost-effectiveness analysis of the therapeutic agent targeting RILI, based directly on patient-level data from a large-scale, multicenter RCT. Building on the survival outcomes observed in our RCT, this analysis further evaluated the QoL and economic feasibility of pirfenidone, highlighting its cost-effective advantage compared with glucocorticoids alone. Both one-way sensitivity analysis and PSA confirm the robustness of these findings. These results are imperative to guide treatment decisions, particularly in resource-constrained scenarios.

The occurrence of APE provides crucial insight into the long-term clinical implications of RILI resolution. APE affects approximately 50% of patients with RILI, defined as unexplained, acute, and clinically significant respiratory deterioration with widespread new-onset alveolar abnormalities on imaging [69]. Given its considerable clinical and economic burden, the APEFS rate emerged as a key factor impacting ICER, ranging from −$72,155 to $127,505 per QALY gained. Correspondingly, the incremental NHB ranged from 0.061 QALY (0.220 versus 0.159 QALY) to −0.006 QALY (0.220 versus 0.226 QALY). Clinically, baseline pulmonary function strongly correlates with APE frequency and HRQoL decrement [70]. APE episodes cause marked declines exceeding 17% in HRQoL, particularly in patients with persistent hypoxemia or poor baseline pulmonary function [70,71]. Consequently, patients undergoing thoracic radiotherapy may experience amplified HRQoL loss from additional APE episodes. Economically, APE management costs escalate steeply with severity. Taking idiopathic pulmonary fibrosis (IPF) as a reference, mild APE episodes typically cost under $1,000, whereas severe episodes requiring hospitalization, complications management, and supplemental oxygen can exceed $15,000, imposing substantial strain on healthcare systems and patients [72]. Given the higher APEFS rate of pirfenidone plus glucocorticoids over glucocorticoids alone (81.7% versus 61.9%) observed in our RCT, pirfenidone offsets its high medication cost by effectively preventing the substantial expenses and QoL loss associated with APE, demonstrating favorable cost-effectiveness. Therefore, the clinical implementation of pirfenidone should be emphasized, especially for those shared poor response to glucocorticoids, severe clinical symptoms, or poor baseline pulmonary preservation.

Current treatment for RILI typically entails empiric administration of glucocorticoids over 8–10 weeks [73]. Clinical evidence shows that approximately 80% of patients achieve transient symptomatic relief due to the potent anti-inflammatory effects of glucocorticoids [74]. Long-term disease control, however, remains suboptimal. Relapse occurs in over 22% of patients, particularly those with severe baseline manifestations, as anti-inflammatory effects attenuate during tapering [8]. In some cases, symptom deterioration or elevated risk of pulmonary fibrosis is also observed [75]. Prolonged administration to control recurrence may result in serious adverse effects, including immunosuppression, opportunistic infections, and osteoporosis [8]. These complications have been linked to a 1.4-fold increase in 5-year mortality, thereby constraining the long-term utility of glucocorticoids in RILI management [76]. Moreover, glucocorticoids lack proven efficacy in treating established pulmonary fibrosis, and their long-term antifibrotic potential remains unvalidated [5]. Our sensitivity analyses further underscore this clinical dilemma. The cost-effectiveness of pirfenidone appears to stem from its capacity to mitigate the long-term limitations of glucocorticoids. As the per-cycle non-response rate increases among patients with grade 2 or 3 RILI receiving standard glucocorticoids, pirfenidone’s clinical benefit in sustaining remission becomes more pronounced, further enhancing its cost-effectiveness. In essence, pirfenidone represents a more durable strategy for sustaining long-term health outcomes as glucocorticoids’ efficacy wanes.

Other variables, such as pirfenidone cost, also contributed to ICER fluctuations. Yet across the full range of variation, the ICER consistently remained below the WTP threshold, spanning from –$3,831 to $45,744 per QALY gained. The incremental NHB further corroborated these findings, ranging from 0.029 QALY (0.227 versus 0.198 QALY) to 0.015 QALY (0.213 versus 0.198 QALY). Pirfenidone’s cost-effectiveness would be overturned only if its unit price increased by 63.8%, corresponding to approximately $5.1 per 200-mg tablet. Conversely, a 16.9% price reduction would render pirfenidone dominant, enhancing clinical outcomes while reducing costs. Overall, the cost-effectiveness of pirfenidone demonstrates robust stability against drug price uncertainties. This robustness is particularly relevant given international variability in pharmaceutical costs, driven by national pricing regulations, out-of-pocket payment structures, and uninsured populations [77]. Encouragingly, the unit price of pirfenidone available through international online pharmacy platform in many healthcare settings, including the United States, Canada, and the United Kingdom, generally remains below $3.0 per 200-mg tablet (Table F in S1 Appendix) [78]. This finding further provides some support for the potential generalizability and scalability of pirfenidone for clinical implementation across diverse healthcare systems.

One of the key factors ensuring that cost-effectiveness results reflect real-world practice is the accurate estimation of costs [79]. Considering that direct costing analysis provides a reliable estimate of healthcare expenditures [80,81], we performed a direct costing analysis of medical expenses during the RCT follow-up period to further assess the credibility of our cost-effectiveness results. The analysis revealed acceptable discrepancies between the observed costs and the model-simulated costs, with the simulation showing increases of 3.3% ($4,864 versus $5,029) and 12.6% ($3,880 versus $4,438), respectively. Several factors may explain these differences. First, due to the multicenter nature of our RCT, variability in the management of severe APE was inevitable [82], and any deviations from guideline-recommended treatments could contribute to discrepancies. Second, from an operational perspective, the RCT was unable to capture non-medical costs, including productivity losses, transportation, and lodging during hospitalization [80,83,84], which present a source of deviation between observed and simulated costs. Notably, these costs were likely more substantial in the standard glucocorticoid group, given its higher rates of severe APE and healthcare utilization, which explains why the deviation in this group was greater than in the pirfenidone group. Nevertheless, this explainable difference further supports the rationality of the model structure and the validity of the underlying data. The direct costing analysis thus provide complementary evidence to reinforce the cost-effectiveness assessment of pirfenidone in the patient population.

Given the heterogeneity in radiotherapy responses and tumor types among enrolled patients [85], we examined the impact of varying baseline health utility values on model outcomes. Utility values spanned the full spectrum in sensitivity analyses, from progressive disease to complete response across thoracic tumors including NSCLC, SCLC, esophageal cancer, and breast cancer. Our results demonstrated that pirfenidone was more cost-effective in patients with lower tumor burden, likely reflecting that RP-related QoL improvements are more apparent in patients with better tumor control [86]. Considering that approximately 50% of patients with thoracic cancer respond to radiotherapy, the actual QoL benefit of pirfenidone for RILI deserves greater attention [87,88]. Importantly, pirfenidone maintained favorable cost-effectiveness across thoracic cancers with various baseline tumor burden, underscoring its therapeutic value across broad clinical subgroups.

Our model is based on robust RCT evidence reflecting the effectiveness of pirfenidone and glucocorticoids. However, certain modeling assumptions warrant clarification. Regarding dosing intensity, full adherence to the target dose of pirfenidone was assumed and did not account for temporary dose reductions or treatment discontinuation. This assumption is supported by the RCT results showing that despite higher tumor burden in the pirfenidone group, approximately 83% of patients completed the full treatment course. These results suggest that pirfenidone has a favorable adherence and tolerability profile compared with agents such as nintedanib, which demonstrates only 72% adherence [10]. Considering the impact of pirfenidone’s cost on the ICER in sensitivity analyses, our model likely overestimated the medication costs of pirfenidone group, thereby underestimating its cost-effectiveness [89]. Regarding indirect costs, productivity loss due to hospitalization for severe RILI or APE was assumed equivalent across treatment arms, without stratifying by actual hospitalization duration. However, the RCT demonstrated a higher APEFS rate and improved pulmonary function with pirfenidone, implying fewer and shorter hospitalizations, and consequently lower wage loss versus standard glucocorticoid therapy [90]. This would further enhance the cost-effectiveness of pirfenidone. Collectively, while our analysis demonstrates substantial cost-effectiveness for pirfenidone, its true economic value compared with glucocorticoids may be even greater in real-world settings.

Several limitations should be acknowledged. First, due to the absence of patient-reported HRQoL data in our phase II trial, utility values and disutility decrements for different RILI grades, AEs, and APE sourced from published literature were not necessarily generalizable to our study population. However, sensitivity analyses confirmed the robustness of the model to variations in these inputs. Second, our RCT population may not fully represent routine clinical practice. Nevertheless, multicenter design of the RCT and our consideration of tumor heterogeneity enhance the external validity of the model [91]. Third, this cost-effectiveness analysis was conducted primarily in the context of China, aligned with the background of our RCT. Further analyses in other geographic settings are warranted to validate our findings. Fourth, this analysis primarily evaluated health benefits using QALYs. Other potential considerations of pirfenidone treatment, such as its impact on patients’ return to work, caregiver burden, and the long-term consequences of complications, were not fully captured. Therefore, in future clinical implementation, treatment strategies should be further optimized by integrating these real-world considerations. Fifth, given the low proportion of immunotherapy exposure in our RCT (approximately 33%) [14], the low incidence of checkpoint inhibitor pneumonitis (CIP) [92], and its similar treatment regimen to RILI [93,94], our study did not account for the impact of immunotherapy exposure. However, our ongoing phase III RCT (NCT07388680) has explicitly distinguished between CIP and RILI to support further exploration. Finally, given the relative cost-effectiveness between pirfenidone and other available anti-fibrotic agents, such as nintedanib, remains controversial [89,95], the optimal long-term therapeutic strategy for RILI still warrants further investigation.

The present analysis primarily focused on the cost-effectiveness of pirfenidone during the early inflammatory phase of RILI, consistent with our RCT design and the predominant anti-inflammatory actions of pirfenidone and glucocorticoids. Notably, previous studies have demonstrated the anti-fibrotic efficacy of pirfenidone in patients with IPF. Long-term treatment has been shown to significantly alleviate dyspnea, with the UCSD Shortness of Breath Questionnaire score improving from 16.504 to 7.760 at 12 months, thus leading to meaningful improvements in patients’ HRQoL [96]. In contrast, the long-term anti-fibrotic efficacy of glucocorticoids has not been clearly established [5]. Therefore, given that pirfenidone has already demonstrated favorable cost-effectiveness during the acute inflammatory phase of RILI, its potential long-term anti-fibrotic effects may further amplify the economic advantage observed in the current analysis. Additionally, our ongoing phase III RCT aims to evaluate the clinical benefits of long-term pirfenidone treatment in patients with RILI. The large sample size and extended follow-up duration of the trial are expected to provide critical parameter estimates for extrapolating cost-effectiveness analyses to longer time horizons [89]. In future studies, incorporating sequential transition states covering both the acute inflammatory phase and the chronic fibrotic phase, together with modeling frameworks using extended time horizons, may allow a more comprehensive assessment of the long-term clinical and economic value of pirfenidone in RILI.

Overall, compared with standard glucocorticoid therapy, pirfenidone plus glucocorticoids demonstrated favorable cost-effectiveness for treating grade 2 or 3 RILI, balancing patient clinical benefits with resource consumption. Our findings provide supportive evidence suggesting that pirfenidone plus glucocorticoids may represent a promising treatment option for RILI and could serve as a reference for healthcare policy development.

### Declarations

#### Ethics approval and consent to participate.

The ethical committee of Sun Yat-sen University Cancer Center (SYSUCC) approved the study protocol (B2021-336) and conducted in accordance with the principles of the Declaration of Helsinki and the Good Clinical Practice guidelines of China. Written informed consent was obtained from all participants before enrollment.

## Supporting information

S1 Appendix Supplementary Materials. **Table A. Model Parameters.** Note. All costs were inflated to year 2025 according to the medical care component of the Consumer Price Index in China and were converted into 2025 US dollars. Unit costs for treatment practice were as follows: X-ray: $3.5 per test; full blood count: $2.6 per test; inflammatory markers: $33.2 per test; blood biochemistry: $17.6 per test; arterial blood gas analysis: $8.3 per test; blood culture: $70.4 per test; sputum culture: $26.7 per test; pulmonary function test: $59.3 per test; Non-contrast CT scan: $32.1 per test; bronchoscopy: $61.4 per test; nursing care: $9.4 per day; bed charges: $6.9 per day; high-flow oxygen supply: $0.96 per hour; low-flow oxygen supply: $0.64 per hour; oral antibiotics: $2.2 per cycle; intravenous glucocorticoids: $7.8 per cycle; intravenous antibiotics: $637.8 per cycle; inhaled bronchodilator: $277.2 per cycle; non-invasive ventilation: $925.8 per cycle; ICU admission: $36,803.0 per cycle. Abbreviations: RILI, radiation-induced lung injury; APE, acute pulmonary exacerbation; ICU, Intensive Care Unit; MIAB, Medical Insurance Administration Bureau of Guangzhou; AE, adverse event; CT, computed tomography. ^†^ Routine laboratory tests refer to selected examinations, including full blood count, inflammatory markers, blood biochemistry, arterial blood gas analysis, blood culture, and sputum culture. Detailed descriptions of the clinical treatment practice for each health state are provided in the Supplementary Methods. ^‡^ When RILI was accompanied by APE, the corresponding clinical management included all treatment practices listed above for RILI plus those indicated for APE, with any overlapping items counted only once. Please see the Supplementary Methods for the full descriptions of the clinical treatment practice for each health state. ^§^ Includes the costs of transportation, accommodation, and additional meal during follow-up. ^¶^ Refers to wage loss related to patients’ and caregivers’ time off work. **Table B. The CHEERS 2022 checklist.** From: Husereau D, Drummond M, Augustovski F, de Bekker-Grob E, Briggs AH, Carswell C, and colleagues. Consolidated Health Economic Evaluation Reporting Standards 2022 (CHEERS 2022) statement: updated reporting guidance for health economic evaluations. BMJ. 2022 Jan 11;376:e067975. https://doi.org/10.1136/bmj-2021-067975. **Table C. Model Validation of the Markov Model-Predicted RILI Pattern Compared with the Real-world Observed RILI Pattern.** Abbreviations: RILI, radiation-induced lung injury. ^†^ The Markov model-predicted RILI pattern presents the distribution of RILI grades simulated at weeks 4, 8, 16, and 24. ^‡^ The real-world observed RILI pattern was derived from the phase II RCT cohort data at the four surveillance time points (weeks 4, 8, 16, and 24). ^§^ The *P*-value was calculated using the Chi-square test. **Table D. Distribution of Cancer Types at Baseline in the Phase II RCT (mITT Population).** Abbreviations: mITT: modified intention to treat; NSCLC, None-small cell lung cancer; SCLC, Small cell lung cancer. **Table E. AE-related clinical scenario cost-effectiveness analysis.** Abbreviations: AE, adverse event; ICER, incremental cost-effectiveness ratio; QALY, quality-adjusted life-year; NHB, net health benefit. **Table F. Summary of pirfenidone unit prices across different countries.** Note: All prices refer to pirfenidone tablets with a strength of 200 mg per tablet. **Fig A. Validation of the Markov model.** The figure demonstrates how the Markov model simulates the 24-week acute pulmonary exacerbation-free survival patterns: (A) pirfenidone plus glucocorticoids (yellow line), (B) glucocorticoids alone (red line) compared with the real-world data (blue lines). The shaded areas represent the 95% confidence intervals of the Kaplan-Meier estimates. Model-simulated survival curves were generated from the Markov model with the base-case model parameters. The P value was calculated using the Log-rank test. **Fig B. Tornado diagram of one-way sensitivity analysis.** The figure depicts the influence of varying all parameters within their predefined ranges on the ICERs between pirfenidone plus glucocorticoids and glucocorticoids alone in patients with grade 2 or 3 RILI. For presentation clarity, the parameters associated with (A) the pirfenidone plus glucocorticoids strategy and (B) the standard glucocorticoid strategy are displayed in separate panels. Blue bars and yellow bars illustrate the ICERs that are greater and less than the base-case ICERs, respectively. Red bars indicate that the ICERs go across the WTP threshold, leading to a switch of the most cost-effective strategy. Numbers on either side of the bars indicate the range of values used for each parameter in the sensitivity analysis. Solid and dashed lines represent the ICERs in the base-case analysis and the WTP threshold of $100,000 per QALY, respectively. Abbreviations: ICU, intensive care unit; APE, acute pulmonary exacerbation; APEFS, acute pulmonary exacerbation free survival; RILI, radiation-induced lung injury; ICER, incremental cost-effectiveness ratio; WTP, willingness-to-pay; QALY, quality-adjusted life-year. **Fig C. Two-way sensitivity analysis of disease progression-related parameters.** The blue area indicates parameter combinations for which pirfenidone plus glucocorticoids is the more cost-effective strategy, whereas the yellow area indicates combinations for which standard glucocorticoid therapy is the more cost-effective strategy, at the WTP threshold of $100,000 per QALY. Abbreviations: RILI, radiation-induced lung injury; APEFS, acute pulmonary exacerbation-free survival; APE, acute pulmonary exacerbation; ICU, intensive care unit. **Fig D. Scatter plot of probabilistic sensitivity analysis for AE-related clinical scenario analysis.** Black line indicates threshold of WTP of $100,000 per QALY. Each point in scatterplot corresponds to one sample of parameter values. Points above reference line indicate higher incremental cost effectiveness ratio than threshold value. The red ellipse represents the 95% confidence ellipse around the mean incremental cost and incremental effectiveness. Abbreviations: AE, adverse event; QALY, quality-adjusted life-year; WTP, willingness-to-pay. **Fig E. Scatter plot of probabilistic sensitivity analysis at the China-specific WTP threshold.** Black line indicates threshold of WTP of $27,680 per QALY. Each point in scatterplot corresponds to one sample of parameter values. Points above reference line indicate higher incremental cost effectiveness ratio than threshold value. The red ellipse represents the 95% confidence ellipse around the mean incremental cost and incremental effectiveness. Abbreviations: QALY, quality-adjusted life-year; WTP, willingness-to-pay.
(DOCX)

S2 Appendix Supplementary Methods. (DOCX)

## Abbreviations

AE: adverse events
APE: acute pulmonary exacerbation
APEFS: acute pulmonary exacerbation-free survival
GDP: gross domestic product
HRQoL: health-related quality of life
ICER: incremental cost-effectiveness ratio
IPF: idiopathic pulmonary fibrosis
ISPOR: International Society for Pharmacoeconomics and Outcomes Research
NHB: net health benefit
PSA: probabilistic sensitivity analyses
QALY: quality-adjusted life year
QoL: quality of life
RCT: randomized controlled trial
RILI: radiation-induced lung injury
WTP: willingness-to-pay

## References

[1] Guo H, Yu R, Zhang H, Wang W. Cytokine, chemokine alterations and immune cell infiltration in radiation-induced lung injury: Implications for prevention and management. Int Immunopharmacol. 2024;126:111263. doi: 10.1016/j.intimp.2023.111263 38000232

[2] Zhao X, Cui K, Bai L, Xu S, Liu W, Shang J, et al. Diagnostic value of SAT-TB in smear-negative pulmonary tuberculosis: a diagnostic accuracy study. Medicine (Baltimore). 2024;103(50):e40907. doi: 10.1097/MD.0000000000040907 39686443 PMC11651478

[3] Käsmann L, Taugner J, Nieto A, Belka C, Eze C, Manapov F. Radiation-induced lung injury: prevention, diagnostics and therapy in the era of the covid-19 pandemic. MDPI; 2022. p. 5713. 10.3390/jcm11195713 PMC9572309 36233578

[4] Ippolito E, Fiore M, Greco C, D’Angelillo RM, Ramella S. COVID-19 and radiation induced pneumonitis: overlapping clinical features of different diseases. Radiother Oncol. 2020;148:201–2. doi: 10.1016/j.radonc.2020.04.009 32342879 PMC7195260

[5] Bledsoe TJ, Nath SK, Decker RH. Radiation pneumonitis. Clin Chest Med. 2017;38(2):201–8. doi: 10.1016/j.ccm.2016.12.004 28477633

[6] Hanania AN, Mainwaring W, Ghebre YT, Hanania NA, Ludwig M. Radiation-induced lung injury: assessment and management. Chest. 2019;156(1):150–62. doi: 10.1016/j.chest.2019.03.033 30998908 PMC8097634

[7] Kumar A. Determining risks and treating of radiation pneumonitis after thoracic radiation. Complications of cancer therapy: best practices in prevention and management: Springer; 2024. p. 211–21.

[8] Yan Y, Fu J, Kowalchuk RO, Wright CM, Zhang R, Li X, et al. Exploration of radiation-induced lung injury, from mechanism to treatment: a narrative review. Transl Lung Cancer Res. 2022;11(2):307–22. doi: 10.21037/tlcr-22-108 35280316 PMC8902083

[9] Komaki R, Lee JS, Milas L, Lee HK, Fossella FV, Herbst RS, et al. Effects of amifostine on acute toxicity from concurrent chemotherapy and radiotherapy for inoperable non-small-cell lung cancer: report of a randomized comparative trial. Int J Radiat Oncol Biol Phys. 2004;58(5):1369–77. doi: 10.1016/j.ijrobp.2003.10.005 15050312

[10] Rimner A, Moore ZR, Lobaugh S, Geyer A, Gelblum DY, Abdulnour R-EE, et al. Randomized phase 2 placebo-controlled trial of nintedanib for the treatment of radiation pneumonitis. Int J Radiat Oncol Biol Phys. 2023;116(5):1091–9. doi: 10.1016/j.ijrobp.2023.02.030 36889516 PMC10751877

[11] Ozturk B, Egehan I, Atavci S, Kitapci M. Pentoxifylline in prevention of radiation-induced lung toxicity in patients with breast and lung cancer: a double-blind randomized trial. Int J Radiat Oncol Biol Phys. 2004;58(1):213–9. doi: 10.1016/s0360-3016(03)01444-5 14697441

[12] Werner-Wasik M, Scott C, Movsas B, Langer C, Sarna L, Nicolaou N, et al. Amifostine as mucosal protectant in patients with locally advanced non-small cell lung cancer (NSCLC) receiving intensive chemotherapy and thoracic radiotherapy (RT): results of the radiation therapy oncology group (RTOG) 98–01 study. Int J Radiat Oncol Biol Phys. 2003;57(2):S216. doi: 10.1016/s0360-3016(03)01029-0

[13] Mell LK, Malik R, Komaki R, Movsas B, Swann RS, Langer C, et al. Effect of amifostine on response rates in locally advanced non-small-cell lung cancer patients treated on randomized controlled trials: a meta-analysis. Int J Radiat Oncol Biol Phys. 2007;68(1):111–8. doi: 10.1016/j.ijrobp.2006.11.043 17289291

[14] Hou Z, Dong B, Yao Q, Chen H, Shao Q, Li M, et al. Pirfenidone for grade 2 and grade 3 radiation-induced lung injury: a multicentre, open-label, randomised, phase 2 trial. Lancet Oncol. 2025;26(12):1552–62. doi: 10.1016/S1470-2045(25)00515-7 41207313

[15] Takeda Y, Tsujino K, Kijima T, Kumanogoh A. Efficacy and safety of pirfenidone for idiopathic pulmonary fibrosis. Patient Prefer Adherence. 2014;8:361–70. doi: 10.2147/PPA.S37233 24711695 PMC3968083

[16] Taniguchi H, Ebina M, Kondoh Y, Ogura T, Azuma A, Suga M, et al. Pirfenidone in idiopathic pulmonary fibrosis. Eur Respir J. 2010;35(4):821–9. doi: 10.1183/09031936.00005209 19996196

[17] Djulbegovic B. Value-based cancer care and the excessive cost of drugs. JAMA Oncol. 2015;1(9):1301–2. doi: 10.1001/jamaoncol.2015.3302 26313294

[18] Tsevat J, Moriates C. Value-Based Health Care Meets Cost-effectiveness Analysis. Ann Intern Med. 2018;169(5):329–32. doi: 10.7326/M18-0342 30083766

[19] Graves PR, Siddiqui F, Anscher MS, Movsas B, editors. Radiation pulmonary toxicity: from mechanisms to management. Seminars in radiation oncology; 2010: Elsevier. 10.1016/j.semradonc.2010.01.010 20685583

[20] Sanders GD, Maciejewski ML, Basu A. Overview of cost-effectiveness analysis. JAMA. 2019;321(14):1400–1. doi: 10.1001/jama.2019.1265 30855638

[21] Ryan KJ, Nero D, Feinberg BA, Lee CH, Pimentel R, Gajra A, et al. Real-world incidence and cost of pneumonitis post-chemoradiotherapy for Stage III non-small-cell lung cancer. Future Oncol. 2020;16(1):4303–13. doi: 10.2217/fon-2019-0524 31802700

[22] U.S. Department Of Health And Human Services. National Institutes of Health National Institutes of Health. Common Terminology Criteria for Adverse Events (CTCAE) Version 5.0 Published: 2017 [cited 25 July 2025]. Available from: https://ctep.cancer.gov/protocoldevelopment/electronic_applications/docs/CTCAE_v5_Quick_Reference_8.5x11.pdf

[23] Leuschner G, Behr J. Acute exacerbation in interstitial lung disease. Front Med (Lausanne). 2017;4:176. doi: 10.3389/fmed.2017.00176 29109947 PMC5660065

[24] Qiu B, Xiong M, Luo Y, Li Q, Chen N, Chen L, et al. Hypofractionated intensity modulated radiation therapy with concurrent chemotherapy in locally advanced non-small cell lung cancer: a phase II prospective clinical trial (GASTO1011). Pract Radiat Oncol. 2021;11(5):374–83. doi: 10.1016/j.prro.2021.06.004 34157448

[25] Smith WP, Richard PJ, Zeng J, Apisarnthanarax S, Rengan R, Phillips MH. Decision analytic modeling for the economic analysis of proton radiotherapy for non-small cell lung cancer. Transl Lung Cancer Res. 2018;7(2):122–33. doi: 10.21037/tlcr.2018.03.27 29876311 PMC5960653

[26] Sanders GD, Neumann PJ, Basu A, Brock DW, Feeny D, Krahn M, et al. Recommendations for conduct, methodological practices, and reporting of cost-effectiveness analyses: second panel on cost-effectiveness in health and medicine. JAMA. 2016;316(10):1093–103. doi: 10.1001/jama.2016.12195 27623463

[27] Palma DA, Senan S, Tsujino K, Barriger RB, Rengan R, Moreno M, et al. Predicting radiation pneumonitis after chemoradiation therapy for lung cancer: an international individual patient data meta-analysis. Int J Radiat Oncol Biol Phys. 2013;85(2):444–50. doi: 10.1016/j.ijrobp.2012.04.043 22682812 PMC3448004

[28] Niska JR, Schild SE, Rule WG, Daniels TB, Jett JR. Fatal radiation pneumonitis in patients with subclinical interstitial lung disease. Clin Lung Cancer. 2018;19(4):e417–20. doi: 10.1016/j.cllc.2018.02.003 29526532

[29] Neumann PJ, Cohen JT, Weinstein MC. Updating cost-effectiveness--the curious resilience of the $50,000-per-QALY threshold. N Engl J Med. 2014;371(9):796–7. doi: 10.1056/NEJMp1405158 25162885

[30] Rao M, Walker S, Claxton K, Bland S, Ochalek J, Phillips A, et al. Guiding Health Resource Allocation: Using Population Net Health Benefit to Align Disease Burden with Cost Effectiveness for Informed Decision Making. Applied Health Economics and Health Policy. 2025:1–8. 10.1007/s40258-025-00964-x PMC12364733 40205294

[31] Wu C-F, Lin L, Mao Y-P, Deng B, Lv J-W, Zheng W-H, et al. Liquid biopsy posttreatment surveillance in endemic nasopharyngeal carcinoma: a cost-effective strategy to integrate circulating cell-free Epstein-Barr virus DNA. BMC Med. 2021;19(1):193. doi: 10.1186/s12916-021-02076-4 34433440 PMC8390246

[32] Husereau D, Drummond M, Augustovski F, de Bekker-Grob E, Briggs AH, Carswell C, et al. Consolidated Health Economic Evaluation Reporting Standards (CHEERS) 2022 explanation and elaboration: a report of the ISPOR CHEERS II good practices task force. Value Health. 2022;25(1):10–31. doi: 10.1016/j.jval.2021.10.008 35031088

[33] Pliakos EE, Andreatos N, Tansarli GS, Ziakas PD, Mylonakis E. The Cost-effectiveness of corticosteroids for the treatment of community-acquired pneumonia. Chest. 2019;155(4):787–94. doi: 10.1016/j.chest.2018.11.001 30448195

[34] Rodriguez-Martinez CE, Nino G, Castro-Rodriguez JA, Perez GF, Sossa-Briceño MP, Buendia JA. Cost-effectiveness analysis of phenotypic-guided versus guidelines-guided bronchodilator therapy in viral bronchiolitis. Pediatr Pulmonol. 2021;56(1):187–95. doi: 10.1002/ppul.25114 33049126 PMC8850934

[35] Gruis KL, Chernew ME, Brown DL. The cost-effectiveness of early noninvasive ventilation for ALS patients. BMC Health Serv Res. 2005;5:58. doi: 10.1186/1472-6963-5-58 16131401 PMC1208883

[36] Ye Y, Zhu B, Jiang L, Jiang Q, Wang M, Hua L, et al. A contemporary assessment of acute mechanical ventilation in Beijing: description, costs, and outcomes. Crit Care Med. 2017;45(7):1160–7. doi: 10.1097/CCM.0000000000002360 28422775 PMC5470868

[37] Ruysscher DD, Wauters E, Jendrossek V, Filippi AR, Revel M-P, Faivre-Finn C, et al. Diagnosis and treatment of radiation induced pneumonitis in patients with lung cancer: an ESTRO clinical practice guideline. Radiother Oncol. 2025;207:110837. doi: 10.1016/j.radonc.2025.110837 40185160

[38] Arroyo-Hernández M, Maldonado F, Lozano-Ruiz F, Muñoz-Montaño W, Nuñez-Baez M, Arrieta O. Radiation-induced lung injury: current evidence. BMC Pulm Med. 2021;21(1):9. doi: 10.1186/s12890-020-01376-4 33407290 PMC7788688

[39] Raghu G, Remy-Jardin M, Richeldi L, Thomson CC, Inoue Y, Johkoh T, et al. Idiopathic pulmonary fibrosis (an update) and progressive pulmonary fibrosis in adults: an official ats/ERS/JRS/ALAT clinical practice guideline. Am J Respir Crit Care Med. 2022;205(9):e18–47. doi: 10.1164/rccm.202202-0399ST 35486072 PMC9851481

[40] Singh J. International society for pharmacoeconomics and outcomes research. Indian J Pharmacol. 2006;38(5):376. doi: 10.4103/0253-7613.27717

[41] Surinach A, Vijapur S, Thiel E. RWD4 a real-world data landscape review of the 2023 International Society for Pharmacoeconomics and Outcomes Research (ISPOR) conference. Value in Health. 2024;27(6):S357. doi: 10.1016/j.jval.2024.03.1658

[42] Huang H-Y, Shi J-F, Guo L-W, Bai Y-N, Liao X-Z, Liu G-X, et al. Expenditure and financial burden for the diagnosis and treatment of colorectal cancer in China: a hospital-based, multicenter, cross-sectional survey. Chin J Cancer. 2017;36(1):41. doi: 10.1186/s40880-017-0209-4 28454595 PMC5410077

[43] Li Q, Jiang W, Wang Q, Shen Y, Gao J, Sato KD, et al. Non-medical financial burden in tuberculosis care: a cross-sectional survey in rural China. Infect Dis Poverty. 2016;5:5. doi: 10.1186/s40249-016-0101-5 26810394 PMC4727322

[44] Kirkland RS, Kole AJ, Batra H, Boggs DH, Spencer SA, Dobelbower MC, et al. Predictors of in-hospital death in patients with lung cancer admitted for acute radiation pneumonitis: a Healthcare Cost and Utilization Project (HCUP) analysis. Clin Lung Cancer. 2021;22(5):e716–22. doi: 10.1016/j.cllc.2021.01.016 33658160

[45] Retèl VP, Steuten LMG, Geukes Foppen MH, Mewes JC, Lindenberg MA, Haanen JBAG, et al. Early cost-effectiveness of tumor infiltrating lymphocytes (TIL) for second line treatment in advanced melanoma: a model-based economic evaluation. BMC Cancer. 2018;18(1):895. doi: 10.1186/s12885-018-4788-5 30219040 PMC6139174

[46] Doyle S, Lloyd A, Walker M. Health state utility scores in advanced non-small cell lung cancer. Lung Cancer. 2008;62(3):374–80. doi: 10.1016/j.lungcan.2008.03.019 18467000

[47] Sawada T, Kondo M, Goto M, Murakami M, Ishida T, Hiroshima Y, et al. Cost-utility analysis of proton beam therapy for locally advanced esophageal cancer in Japan. PLoS One. 2024;19(9):e0308961. doi: 10.1371/journal.pone.0308961 39331653 PMC11433116

[48] Butler JRG, McIntyre P, MacIntyre CR, Gilmour R, Howarth AL, Sander B. The cost-effectiveness of pneumococcal conjugate vaccination in Australia. Vaccine. 2004;22(9–10):1138–49. doi: 10.1016/j.vaccine.2003.09.036 15003641

[49] Loveman E, Copley VR, Colquitt JL, Scott DA, Clegg AJ, Jones J, et al. The effectiveness and cost-effectiveness of treatments for idiopathic pulmonary fibrosis: systematic review, network meta-analysis and health economic evaluation. BMC Pharmacol Toxicol. 2014;15:63. doi: 10.1186/2050-6511-15-63 25410822 PMC4247619

[50] Detsky AS, Naglie IG. A clinician’s guide to cost-effectiveness analysis. Ann Intern Med. 1990;113(2):147–54. doi: 10.7326/0003-4819-113-2-147 2113784

[51] Ricardi U, Filippi AR, Guarneri A, Giglioli FR, Ciammella P, Franco P, et al. Stereotactic body radiation therapy for early stage non-small cell lung cancer: results of a prospective trial. Lung Cancer. 2010;68(1):72–7. doi: 10.1016/j.lungcan.2009.05.007 19556022

[52] Baker R, Han G, Sarangkasiri S, DeMarco M, Turke C, Stevens CW, et al. Clinical and dosimetric predictors of radiation pneumonitis in a large series of patients treated with stereotactic body radiation therapy to the lung. Int J Radiat Oncol Biol Phys. 2013;85(1):190–5. doi: 10.1016/j.ijrobp.2012.03.041 22929858

[53] Kocak Z, Evans ES, Zhou S-M, Miller KL, Folz RJ, Shafman TD, et al. Challenges in defining radiation pneumonitis in patients with lung cancer. Int J Radiat Oncol Biol Phys. 2005;62(3):635–8. doi: 10.1016/j.ijrobp.2004.12.023 15936538

[54] Thomas R, Chen Y-H, Hatabu H, Mak RH, Nishino M. Radiographic patterns of symptomatic radiation pneumonitis in lung cancer patients: imaging predictors for clinical severity and outcome. Lung Cancer. 2020;145:132–9. doi: 10.1016/j.lungcan.2020.03.023 32447116 PMC7316126

[55] Shintani T, Kishi N, Matsuo Y, Ogura M, Mitsuyoshi T, Araki N, et al. Incidence and risk factors of symptomatic radiation pneumonitis in non-small-cell lung cancer patients treated with concurrent chemoradiotherapy and consolidation durvalumab. Clin Lung Cancer. 2021;22(5):401–10. doi: 10.1016/j.cllc.2021.01.017 33678582

[56] Guckenberger M, Baier K, Polat B, Richter A, Krieger T, Wilbert J, et al. Dose-response relationship for radiation-induced pneumonitis after pulmonary stereotactic body radiotherapy. Radiother Oncol. 2010;97(1):65–70. doi: 10.1016/j.radonc.2010.04.027 20605245

[57] Yamashita H, Kobayashi-Shibata S, Terahara A, Okuma K, Haga A, Wakui R, et al. Prescreening based on the presence of CT-scan abnormalities and biomarkers (KL-6 and SP-D) may reduce severe radiation pneumonitis after stereotactic radiotherapy. Radiat Oncol. 2010;5:32. doi: 10.1186/1748-717X-5-32 20459699 PMC2876174

[58] Pourrahmat M-M, Kim A, Kansal AR, Hux M, Pushkarna D, Fazeli MS, et al. Health state utility values by cancer stage: a systematic literature review. Eur J Health Econ. 2021;22(8):1275–88. doi: 10.1007/s10198-021-01335-8 34125315 PMC8526485

[59] Vedadi A, Shakik S, Brown MC, Lok BH, Shepherd FA, Leighl NB, et al. The impact of symptoms and comorbidity on health utility scores and health-related quality of life in small cell lung cancer using real world data. Qual Life Res. 2021;30(2):445–54. doi: 10.1007/s11136-020-02615-1 32851601

[60] Doherty MK, Leung Y, Su J, Naik H, Patel D, Eng L, et al. Health utility scores from EQ-5D and health-related quality of life in patients with esophageal cancer: a real-world cross-sectional study. Dis Esophagus. 2018;31(12):10.1093/dote/doy058. doi: 10.1093/dote/doy058 29905764

[61] Kaur MN, Yan J, Klassen AF, David JP, Pieris D, Sharma M, et al. A systematic literature review of health utility values in breast cancer. Med Decis Making. 2022;42(5):704–19. doi: 10.1177/0272989X211065471 35042379 PMC9189726

[62] Ara R, Brazier JE. Using health state utility values from the general population to approximate baselines in decision analytic models when condition-specific data are not available. Value Health. 2011;14(4):539–45. doi: 10.1016/j.jval.2010.10.029 21669378

[63] Hu W, Zhou L, Chu J, Sun N, Chen X, Liu S, et al. Estimating population norms for the health-related quality of life of adults in southern Jiangsu Province, China. Sci Rep. 2022;12(1):9906. doi: 10.1038/s41598-022-13910-x 35701516 PMC9198056

[64] Liu X, Zhou H, Wei J, Li M, Luo G, Naidoo N, et al. An occupational health survey on health utility and occupational diseases in Chinese university staff to inform cost-utility analysis. Front Public Health. 2023;10:1022344. doi: 10.3389/fpubh.2022.1022344 36703839 PMC9871467

[65] Dang J, Li G, Ma L, Diao R, Zang S, Han C, et al. Predictors of grade ≥ 2 and grade ≥ 3 radiation pneumonitis in patients with locally advanced non-small cell lung cancer treated with three-dimensional conformal radiotherapy. Acta Oncol. 2013;52(6):1175–80. doi: 10.3109/0284186X.2012.747696 23198719

[66] Shi G, Park SH, Ren H, Xue M, Lu X, Dong P, et al. Cost analysis for different sequential treatment regimens for metastatic renal cell carcinoma in China. J Med Econ. 2018;21(12):1150–8. doi: 10.1080/13696998.2018.1515769 30134758

[67] Chen J, Hu G, Chen Z, Wan X, Tan C, Zeng X, et al. Cost-effectiveness analysis of pembrolizumab plus axitinib versus sunitinib in first-line advanced renal cell carcinoma in China. Clin Drug Investig. 2019;39(10):931–8. doi: 10.1007/s40261-019-00820-6 31250401

[68] Wu J, Liu GG. Comments on the 2025 edition of China guidelines for pharmacoeconomic evaluations. Pharmacoecon Policy. 2026;2(1):1–3. doi: 10.1016/j.pharp.2026.03.003

[69] Williams JP, Johnston CJ, Finkelstein JN. Treatment for radiation-induced pulmonary late effects: spoiled for choice or looking in the wrong direction?. Curr Drug Targets. 2010;11(11):1386–94. doi: 10.2174/1389450111009011386 20583979 PMC2948640

[70] Kreuter M, Wuyts WA, Wijsenbeek M, Bajwah S, Maher TM, Stowasser S, et al. Health-related quality of life and symptoms in patients with IPF treated with nintedanib: analyses of patient-reported outcomes from the INPULSIS® trials. Respir Res. 2020;21(1):36. doi: 10.1186/s12931-020-1298-1 32000772 PMC6990488

[71] Koyama K, Sakamoto S, Isshiki T, Shimizu H, Kurosaki A, Homma S. The activities of daily living after an acute exacerbation of idiopathic pulmonary fibrosis. Intern Med. 2017;56(21):2837–43. doi: 10.2169/internalmedicine.7875-16 28943534 PMC5709624

[72] Loveman E, Copley VR, Colquitt J, Scott DA, Clegg A, Jones J, et al. The clinical effectiveness and cost-effectiveness of treatments for idiopathic pulmonary fibrosis: a systematic review and economic evaluation. Health Technol Assess. 2015;19(20):i–xxiv, 1–336. doi: 10.3310/hta19200 25760991 PMC4781002

[73] Voruganti Maddali IS, Cunningham C, McLeod L, Bahig H, Chaudhuri N, L M Chua K, et al. Optimal management of radiation pneumonitis: findings of an international Delphi consensus study. Lung Cancer. 2024;192:107822. doi: 10.1016/j.lungcan.2024.107822 38788551

[74] Rahi MS, Parekh J, Pednekar P, Parmar G, Abraham S, Nasir S, et al. Radiation-induced lung injury-current perspectives and management. Clin Pract. 2021;11(3):410–29. doi: 10.3390/clinpract11030056 34287252 PMC8293129

[75] Käsmann L, Dietrich A, Staab-Weijnitz CA, Manapov F, Behr J, Rimner A, et al. Radiation-induced lung toxicity - cellular and molecular mechanisms of pathogenesis, management, and literature review. Radiat Oncol. 2020;15(1):214. doi: 10.1186/s13014-020-01654-9 32912295 PMC7488099

[76] Einarsdottir MJ, Ekman P, Molin M, Trimpou P, Olsson DS, Johannsson G, et al. High mortality rate in oral glucocorticoid users: a population-based matched cohort study. Front Endocrinol (Lausanne). 2022;13:918356. doi: 10.3389/fendo.2022.918356 35872995 PMC9304700

[77] Wong AW, Koo J, Ryerson CJ, Sadatsafavi M, Chen W. A systematic review on the economic burden of interstitial lung disease and the cost-effectiveness of current therapies. BMC Pulm Med. 2022;22(1):148. doi: 10.1186/s12890-022-01922-2 35443657 PMC9020025

[78] [Internet]. PcPp. New York: PharmacyChecker [cited 6 March 2026]. Avaliable from: https://www.pharmacychecker.com/pirfenidone/#prices

[79] Turner HC, Rivillas-Garcia JC, Prinja S, Hung TM, Dabak SV, Asare BA, et al. An introduction to costing and the types of costs used within health economic studies: HC Turner et al. PharmacoEconomics-Open. 2025:1–20. 10.1007/s41669-025-00602-1 PMC12559562 41114877

[80] Tan SS, Bakker J, Hoogendoorn ME, Kapila A, Martin J, Pezzi A, et al. Direct cost analysis of intensive care unit stay in four European countries: applying a standardized costing methodology. Value Health. 2012;15(1):81–6. doi: 10.1016/j.jval.2011.09.007 22264975

[81] Coughlan D, Yeh ST, O’Neill C, Frick KD. Evaluating direct medical expenditures estimation methods of adults using the medical expenditure panel survey: an example focusing on head and neck cancer. Value Health. 2014;17(1):90–7. doi: 10.1016/j.jval.2013.10.004 24438722

[82] Group WS. A guide on organizing a multicenter clinical trial: the WRIST study group. Plast Reconstr Surg. 2010;126:515–23. 20375760 10.1097/PRS.0b013e3181df64fa PMC2917608

[83] Urban JA. Cost analysis of surgical site infections. Surg Infect (Larchmt). 2006;7 Suppl 1:S19-22. doi: 10.1089/sur.2006.7.s1-19 16834543

[84] Mokri H, Kvamme I, de Vries L, Versteegh M, van Baal P. Future medical and non-medical costs and their impact on the cost-effectiveness of life-prolonging interventions: a comparison of five European countries. Eur J Health Econ. 2023;24(5):701–15. doi: 10.1007/s10198-022-01501-6 35925501 PMC10198844

[85] Chvetsov AV, Yartsev S, Schwartz JL, Mayr N. Assessment of interpatient heterogeneity in tumor radiosensitivity for nonsmall cell lung cancer using tumor-volume variation data. Med Phys. 2014;41(6):064101. doi: 10.1118/1.4875686 24877843

[86] Pearce A, Haas M, Viney R. Are the true impacts of adverse events considered in economic models of antineoplastic drugs? A systematic review. Appl Health Econ Health Policy. 2013;11(6):619–37. doi: 10.1007/s40258-013-0058-5 24129649

[87] Li Y, Huang H-C, Chen L-Q, Xu L-Y, Li E-M, Zhang J-J. Predictive biomarkers for response of esophageal cancer to chemo(radio)therapy: a systematic review and meta-analysis. Surg Oncol. 2017;26(4):460–72. doi: 10.1016/j.suronc.2017.09.003 29113666

[88] Deng W, Lin SH. Advances in radiotherapy for esophageal cancer. Ann Transl Med. 2018;6(4):79. doi: 10.21037/atm.2017.11.28 29666802 PMC5890036

[89] Clay E, Cristeau O, Chafaie R, Pinta A, Mazaleyrat B, Cottin V. Cost-effectiveness of pirfenidone compared to all available strategies for the treatment of idiopathic pulmonary fibrosis in France. J Mark Access Health Policy. 2019;7(1):1626171. doi: 10.1080/20016689.2019.1626171 31275535 PMC6598518

[90] Mennini FS, Gitto L. Aapproaches to estimating indirect costs in healthcare: motivations for choice. JEE. 2022;21(1):17–45. doi: 10.35774/jee2022.01.017

[91] Ramsey S, Willke R, Briggs A, Brown R, Buxton M, Chawla A, et al. Good research practices for cost-effectiveness analysis alongside clinical trials: the ISPOR RCT-CEA Task Force report. Value Health. 2005;8(5):521–33. doi: 10.1111/j.1524-4733.2005.00045.x 16176491

[92] Ma K, Lu Y, Jiang S, Tang J, Li X, Zhang Y. The relative risk and incidence of immune checkpoint inhibitors related pneumonitis in patients with advanced cancer: a meta-analysis. Front Pharmacol. 2018;9:1430. doi: 10.3389/fphar.2018.01430 30618738 PMC6297260

[93] Brahmer JR, Lacchetti C, Schneider BJ, Atkins MB, Brassil KJ, Caterino JM, et al. Management of immune-related adverse events in patients treated with immune checkpoint inhibitor therapy: American Society of Clinical Oncology Clinical Practice Guideline. J Clin Oncol. 2018;36(17):1714–68. doi: 10.1200/JCO.2017.77.6385 29442540 PMC6481621

[94] Haanen JBAG, Carbonnel F, Robert C, Kerr KM, Peters S, Larkin J, et al. Management of toxicities from immunotherapy: ESMO Clinical Practice Guidelines for diagnosis, treatment and follow-up. Ann Oncol. 2017;28(suppl_4):iv119–42. doi: 10.1093/annonc/mdx225 28881921

[95] Rinciog C, Diamantopoulos A, Gentilini A, Bondue B, Dahlqvist C, Froidure A, et al. Cost-effectiveness analysis of nintedanib versus pirfenidone in idiopathic pulmonary fibrosis in Belgium. Pharmacoecon Open. 2020;4(3):449–58. doi: 10.1007/s41669-019-00191-w 31939146 PMC7426351

[96] Kang M, Gupta S, Tu Y-H, Raimundo K, Patel AM, Flaherty KR. Impact of pirfenidone on patient-reported outcomes in patients with idiopathic pulmonary fibrosis from the Pulmonary Fibrosis Foundation Patient Registry. CHEST Pulmonary. 2024;2(4):100082. doi: 10.1016/j.chpulm.2024.100082 42548421 PMC13417587

