# Supplementary material for: Cost-effectiveness of pirfenidone for patients with cancer and grade 2 or grade 3 radiation-induced lung injury: A modeling study
Source: PLoS Med. 2026 Aug 5;23(8):e1004817. doi: 10.1371/journal.pmed.1004817 (PMC13440887; doi:10.1371/journal.pmed.1004817)
Supplement: Supplementary file 1 [file pmed.1004817.s001.docx]

| Content | | Page |
| --- | --- | --- |
| Table A | Model Parameters | 1-6 |
| Table B | The CHEERS 2022 Checklist | 7-9 |
| Table C | Model Validation of the Markov Model-Predicted RILI Pattern Compared with the Real-world Observed RILI Pattern | 10-11 |
| Table D | Distribution of Cancer Types at Baseline in the Phase II RCT (mITT Population) | 12 |
| Table E | AE-related clinical scenario cost-effectiveness analysis | 13 |
| Table F | Summary of Pirfenidone Unit Prices Across Different Countries | 14 |
| Fig A | Validation of the Markov Model | 15 |
| Fig B | Tornado Diagram of One-Way Sensitivity Analysis | 16-17 |
| Fig C | Two-Way Sensitivity Analysis of Disease Progression-related Parameters | 18 |
| Fig D | Scatter plot of probabilistic sensitivity analysis for AE-related clinical scenario analysis. | 19 |
| Fig E | Scatter plot of probabilistic sensitivity analysis at the China-specific WTP threshold. | 20 |

**S1 Appendix. Supplementary Materials**

**Table A.** **Model Parameters**

| **Parameter** | **Pirfenidone plus glucocorticoids** | | **Glucocorticoids alone** | | **Distribution** | **Reference** |
| --- | --- | --- | --- | --- | --- | --- |
|  | **Base-Case Estimate** | **Range** | **Base-Case Estimate** | **Range** |  |  |
| **Transition probabilities (per 4 weeks)** | | | | | | |
| Probability of transition from each health state to no RILI | | | | | | |
| No RILI | 0.921 | 0.737-1.000 | 0.993 | 0.794-1.000 | Beta | Trial cohort |
| Grade 1 RILI | 0.108 | 0.087-0.130 | 0.000 | 0.000-0.200 | Beta | Trial cohort |
| Grade 2 or 3 RILI | 0.046 | 0.037-0.056 | 0.018 | 0.014-0.022 | Beta | Trial cohort |
| Grade 4 RILI | 0.000 | 0.000-0.200 | 0.000 | 0.000-0.200 | Beta | Trial cohort |
| Probability of transition from each health state to grade 1 RILI | | | | | | |
| No RILI | 0.071 | 0.057-0.085 | 0.000 | 0.000-0.200 | Beta | Trial cohort |
| Grade 1 RILI | 0.775 | 0.620-0.930 | 0.722 | 0.578-0.867 | Beta | Trial cohort |
| Grade 2 or 3 RILI | 0.306 | 0.245-0.367 | 0.193 | 0.154-0.231 | Beta | Trial cohort |
| Grade 4 RILI | 0.084 | 0.067-0.100 | 0.034 | 0.027-0.041 | Beta | Trial cohort |
| Probability of transition from each health state to grade 2 or 3 RILI | | | | | | |
| No RILI | 0.000 | 0.000-0.200 | 0.000 | 0.000-0.200 | Beta | Trial cohort |
| Grade 1 RILI | 0.108 | 0.087-0.130 | 0.271 | 0.217-0.325 | Beta | Trial cohort |
| Grade 2 or 3 RILI | 0.639 | 0.512-0.767 | 0.764 | 0.611-0.917 | Beta | Trial cohort |
| Grade 4 RILI | 0.451 | 0.361-0.541 | 0.361 | 0.289-0.433 | Beta | Trial cohort |
| Probability of transition from each health state to grade 4 RILI | | | | | | |
| No RILI | 0.000 | 0.000-0.012 | 0.000 | 0.000-0.012 | Beta | Trial cohort, [50-56] |
| Grade 1 RILI | 0.000 | 0.000-0.012 | 0.000 | 0.000-0.012 | Beta | Trial cohort, [50-56] |
| Grade 2 or 3 RILI | 0.000 | 0.000-0.012 | 0.000 | 0.000-0.012 | Beta | Trial cohort, [50-56] |
| Grade 4 RILI | 0.456 | 0.365-0.548 | 0.565 | 0.452-0.678 | Beta | Trial cohort, [50-56] |
| Probability of transition from each health state to grade 5 RILI | | | | | | |
| No RILI | 0.000 | 0.000-0.020 | 0.000 | 0.000-0.020 | Beta | Trial cohort, [26, 27] |
| Grade 1 RILI | 0.000 | 0.000-0.020 | 0.000 | 0.000-0.020 | Beta | Trial cohort, [26, 27] |
| Grade 2 or 3 RILI | 0.000 | 0.000-0.020 | 0.018 | 0.014-0.022 | Beta | Trial cohort, [26, 27] |
| Grade 4 RILI | 0.000 | 0.000-0.020 | 0.034 | 0.027-0.041 | Beta | Trial cohort, [26, 27] |
| Probability of transition from each health state to death due to other causes | | | | | | |
| Death due to other causes | 0.008 | 0.007-0.010 | 0.007 | 0.006-0.008 **Treatment strategy** \| **Total values** \| \| \| **Incremental values** \| \| \| **ICER ($/QALY)** \| **NHB (QALY)**; \| **Cost ($)** \| \| **Effectiveness (QALY)** \| **Cost ($)** \| \| **Effectiveness (QALY)** \| \|; Glucocorticoids \| 4,358 \| \| 0.242 \| — \| \| — \| — \| 0.198; Pirfenidone plus glucocorticoids \| 4,953 \| \| 0.270 \| 595 \| \| 0.028 \| 21,129 \| 0.220 | Beta | Trial cohort |
| Probability of no APE occurrence in each health state | | | | | | |
| No RILI | 1.000 | 0.800-1.000 | 1.000 | 0.800-1.000 | Beta | Trial cohort |
| Grade 1 RILI | 0.948 | 0.759-1.000 | 0.844 | 0.676-1.000 | Beta | Trial cohort |
| Grade 2 or 3 RILI | 0.935 | 0.748-1.000 | 0.894 | 0.716-1.000 | Beta | Trial cohort |
| Grade 4 RILI | 0.930 | 0.744-1.000 | 0.800 | 0.640-0.960 | Beta | Trial cohort |
| Probability of APE occurrence in each health state | | | | | | |
| No RILI | 0.000 | 0.000-0.200 | 0.000 | 0.000-0.200 | Beta | Trial cohort |
| Grade 1 RILI | 0.052 | 0.041-0.062 | 0.156 | 0.124-0.187 | Beta | Trial cohort |
| Grade 2 or 3 RILI | 0.065 | 0.052-0.077 | 0.106 | 0.084-0.127 | Beta | Trial cohort |
| Grade 4 RILI | 0.070 | 0.056-0.084 | 0.200 | 0.160-0.240 | Beta | Trial cohort |
| Probability of mild APE occurrence in each health state | | | | | | |
| No RILI | 1.000 | 0.800-1.000 | 1.000 | 0.800-1.000 | Beta | Trial cohort |
| Grade 1 RILI | 1.000 | 0.800-1.000 | 0.857 | 0.686-1.000 | Beta | Trial cohort |
| Grade 2 or 3 RILI | 0.625 | 0.500-0.750 | 0.526 | 0.421-0.632 | Beta | Trial cohort |
| Grade 4 RILI | 0.410 | 0.328-0.492 | 0.327 | 0.261-0.392 | Beta | Trial cohort |
| Probability of severe APE occurrence in each health state | | | | | | |
| No RILI | 0.000 | 0.000-0.200 | 0.000 | 0.000-0.200 | Beta | Trial cohort |
| Grade 1 RILI | 0.000 | 0.000-0.200 | 0.143 | 0.114-0.171 | Beta | Trial cohort |
| Grade 2 or 3 RILI | 0.375 | 0.300-0.450 | 0. 474 | 0.379-0.568 | Beta | Trial cohort |
| Grade 4 RILI | 0.590 | 0.472-0.708 | 0.673 | 0.539-0.808 | Beta | Trial cohort |
| Probability of ICU admission-free due to grade 2 or 3 RILI plus severe APE | | | | | | |
| Grade 2 or 3 RILI | 1.000 | 0.800-1.000 | 0.600 | 0.480-0.720 | Beta | Trial cohort |
| Probability of ICU admission due to grade 2 or 3 RILI plus severe APE | | | | | | |
| Grade 2 or 3 RILI | 0.000 | 0.000-0.200 | 0.400 | 0.320-0.480 | Beta | Trial cohort |
| Proportion of grade 3 among grade 2 or 3 RILI health state | | | | | | |
| Grade 2 or 3 RILI | 0.024 | 0.019-0.029 | 0.045 | 0.036-0.054 | Beta | Trial cohort |
| Probability of patients with mild APE | | | | | | |
| Recovery from mild APE | 0.875 | 0.700-1.000 | 0.706 | 0.565-0.847 | Beta | Trial cohort |
| Persistence of mild APE | 0.125 | 0.100-0.150 | 0.294 | 0.235-0.353 | Beta | Trial cohort |
| Death due to mild APE | 0.000 | 0.000-0.200 | 0.000 | 0.000-0.200 | Beta | Trial cohort |
| Probability of patients with severe APE | | | | | | |
| Recovery from severe APE | 0.333 | 0.267-0.400 | 0.000 | 0.000-0.200 | Beta | Trial cohort |
| Improvement from severe APE | 0.000 | 0.000-0.200 | 0.444 | 0.356-0.533 | Beta | Trial cohort |
| Persistence of severe APE | 0.333 | 0.267-0.400 | 0.556 | 0.444-0.667 | Beta | Trial cohort |
| Death due to severe APE | 0.333 | 0.267-0.400 | 0.000 | 0.000-0.200 | Beta | Trial cohort |
| Incidence of grade 1-2 AEs (pirfenidone) | 0.139 | 0.111-0.167 | — | — | Beta | Trial cohort |
| Incidence of grade 3-4 AEs (pirfenidone) | 0.017 | 0.014-0.020 | — | — | Beta | Trial cohort |
| Discount rate | 0.030 | 0.010-0.050 | 0.030 | 0.010-0.050 | Beta | [25] |
| **Costs (per 4 weeks/$)** | | | | | | |
| Costs of basic medication | | | | | | |
| Oral glucocorticoids | 0.3 | 0.3-0.4 | 0.3 | 0.3-0.4 | Gamma | MIAB |
| Pirfenidone | 606.0 | 485-727 | — | — | Gamma | MIAB |
| Costs of additional treatment practice | | | | | | |
| Grade 1 RILI | 92.0 | 74-110 | 92.0 | 74-110 | Gamma | MIAB |
| X-ray | 3.5 | 3-4 | 3.5 | 3-4 | Gamma | MIAB |
| Routine laboratory tests^†^ | 88.5 | 71-106 | 88.5 | 71-106 | Gamma | MIAB |
| Grade 2 or 3 RILI | 453.6 | 363-544 | 471.3 | 377-566 | Gamma | MIAB |
| Grade 2 RILI | 433.2 | 347-520 | 433.2 | 347-520 | Gamma | MIAB |
| Symptom treatment | 149.1 | 119-179 | 149.1 | 119-179 | Gamma | MIAB |
| Routine imaging tests | 42.5 | 34-51 | 42.5 | 34-51 | Gamma | MIAB |
| Non-contrast CT scan | 32.1 | 26-39 | 32.1 | 26-39 | Gamma | MIAB |
| X-ray | 10.4 | 8-12 | 10.4 | 8-12 | Gamma | MIAB |
| Routine laboratory tests^†^ | 120.9 | 97-145 | 120.9 | 97-145 | Gamma | MIAB |
| Pulmonary function test | 59.3 | 47-71 | 59.3 | 47-71 | Gamma | MIAB |
| Bronchoscopy | 61.4 | 49-74 | 61.4 | 49-74 | Gamma | MIAB |
| Grade 3 RILI | 1277.2 | 1022-1533 | 1277.2 | 1022-1533 | Gamma | MIAB |
| Symptom treatment | 149.1 | 119-179 | 149.1 | 119-179 | Gamma | MIAB |
| Oral antibiotics | 2.2 | 2-3 | 2.2 | 2-3 | Gamma | MIAB |
| Intravenous glucocorticoids | 7.8 | 6-9 | 7.8 | 6-9 | Gamma | MIAB |
| Low-flow oxygen therapy | 763.7 | 611-916 | 763.7 | 611-916 | Gamma | MIAB |
| Level II nursing care costs | 140.0 | 112-168 | 140.0 | 112-168 | Gamma | MIAB |
| Bed charges | 194.4 | 156-233 | 194.4 | 156-233 | Gamma | MIAB |
| Oxygen supply | 429.3 | 343-515 | 429.3 | 343-515 | Gamma | MIAB |
| Routine imaging tests | 42.5 | 34-51 | 42.5 | 34-51 | Gamma | MIAB |
| Non-contrast CT scan | 32.1 | 26-39 | 32.1 | 26-39 | Gamma | MIAB |
| X-ray | 10.4 | 8-12 | 10.4 | 8-12 | Gamma | MIAB |
| Routine laboratory tests^†^ | 191.3 | 153-230 | 191.3 | 153-230 | Gamma | MIAB |
| Pulmonary function test | 59.3 | 47-71 | 59.3 | 47-71 | Gamma | MIAB |
| Bronchoscopy | 61.4 | 49-74 | 61.4 | 49-74 | Gamma | MIAB |
| Grade 4 RILI | 2352.2 | 1882-2823 | 2352.2 | 1882-2823 | Gamma | MIAB |
| Symptom treatment | 149.1 | 119-179 | 149.1 | 119-179 | Gamma | MIAB |
| Intravenous antibiotics | 637.8 | 510-765 | 637.8 | 510-765 | Gamma | [32] |
| Intravenous glucocorticoids | 7.8 | 6-9 | 7.8 | 6-9 | Gamma | MIAB |
| Non-invasive ventilation | 925.8 | 741-1111 | 925.8 | 741-1111 | Gamma | [34] |
| Inhaled bronchodilator | 277.2 | 222-333 | 277.2 | 222-333 | Gamma | [33] |
| Routine imaging tests | 42.5 | 34-51 | 42.5 | 34-51 | Gamma | MIAB |
| Non-contrast CT scan | 32.1 | 26-39 | 32.1 | 26-39 | Gamma | MIAB |
| X-ray | 10.4 | 8-12 | 10.4 | 8-12 | Gamma | MIAB |
| Routine laboratory tests^†^ | 191.3 | 153-230 | 191.3 | 153-230 | Gamma | MIAB |
| Pulmonary function test | 59.3 | 47-71 | 59.3 | 47-71 | Gamma | MIAB |
| Bronchoscopy | 61.4 | 49-74 | 61.4 | 49-74 | Gamma | MIAB |
| ICU admission | 36803.0 | 29442-44164 | 36803.0 | 29442-44164 | Gamma | [35] |
| Mild APE^†^ | 133.6 | 107-160 | 133.6 | 107-160 | Gamma | MIAB |
| Oral antibiotics | 2.2 | 2-3 | 2.2 | 2-3 | Gamma | MIAB |
| Symptom treatment | 74.6 | 60-90 | 74.6 | 60-90 | Gamma | MIAB |
| X-ray | 3.5 | 3-4 | 3.5 | 3-4 | Gamma | MIAB |
| Routine laboratory tests^†^ | 53.4 | 43-64 | 53.4 | 43-64 | Gamma | MIAB |
| Severe APE^‡^ | 2182.7 | 1746-2619 | 2182.7 | 1746-2619 | Gamma | MIAB |
| Intravenous antibiotics | 637.8 | 510-765 | 637.8 | 510-765 | Gamma | [32] |
| Intravenous glucocorticoids | 7.8 | 6-9 | 7.8 | 6-9 | Gamma | MIAB |
| Symptom treatment | 149.1 | 119-179 | 149.1 | 119-179 | Gamma | MIAB |
| High-flow oxygen therapy | 1102.7 | 882-1323 | 1102.7 | 882-1323 | Gamma | MIAB |
| Level I nursing care costs | 264.4 | 212-317 | 264.4 | 212-317 | Gamma | MIAB |
| Bed charges | 194.4 | 156-233 | 194.4 | 156-233 | Gamma | MIAB |
| Oxygen supply | 643.9 | 515-773 | 643.9 | 515-773 | Gamma | MIAB |
| X-ray | 3.5 | 3-4 | 3.5 | 3-4 | Gamma | MIAB |
| Routine laboratory tests^†^ | 220.4 | 176-264 | 220.4 | 176-264 | Gamma | MIAB |
| Bronchoscopy | 61.4 | 49-74 | 61.4 | 49-74 | Gamma | MIAB |
| Grade 1-2 AEs (pirfenidone) | 11.2 | 9-13 | — | — | Gamma | MIAB |
| Grade 3-4 AEs (pirfenidone) | 199.9 | 160-240 | — | — | Gamma | MIAB |
| Direct non-health care cost^§^ | 37.4 | 30-45 | 37.4 | 30-45 | Gamma | [42, 43] |
| Additional nutrition for inpatients | 28.4 | 23-34 | 28.4 | 23-34 | Gamma | [43] |
| Indirect cost^¶^ | 370.8 | 297-445 | 370.8 | 297-445 | Gamma | [44] |
| **Utility** |  |  |  |  |  |  |
| Baseline utility | 0.712 | 0.600-0.981 | 0.712 | 0.600-0.981 | Beta | [46, 61-63] |
| Grade 1 RILI | 0.672 | 0.538-0.806 | 0.672 | 0.538-0.806 | Beta | [47] |
| Grade 2 or 3 RILI | 0.526 | 0.420-0.631 | 0.526 | 0.420-0.631 | Beta | [46, 48] |
| Grade 4 RILI | 0.339 | 0.271-0.407 | 0.339 | 0.271-0.407 | Beta | [46, 48] |
| Grade 1-2 AEs decrement (pirfenidone) | 0.014 | 0.011-0.017 | — | — | Beta | [66, 67] |
| Grade 3-4 AEs decrement (pirfenidone) | 0.157 | 0.126-0.188 | — | — | Beta | [66, 67] |
| Mild APE decrement | 0.048 | 0.036-0.060 | 0.048 | 0.036-0.060 | Beta | [46] |
| Severe APE decrement | 0.200 | 0.160-0.240 | 0.200 | 0.160-0.240 | Beta | [49] |

Note. All costs were inflated to year 2025 according to the medical care component of the Consumer Price Index in China and were converted into 2025 US dollars. Unit costs for treatment practice were as follows: X-ray: $3.5 per test; full blood count: $2.6 per test; inflammatory markers: $33.2 per test; blood biochemistry: $17.6 per test; arterial blood gas analysis: $8.3 per test; blood culture: $70.4 per test; sputum culture: $26.7 per test; pulmonary function test: $59.3 per test; Non-contrast CT scan: $32.1 per test; bronchoscopy: $61.4 per test; nursing care: $9.4 per day; bed charges: $6.9 per day; high-flow oxygen supply: $0.96 per hour; low-flow oxygen supply: $0.64 per hour; oral antibiotics: $2.2 per cycle; intravenous glucocorticoids: $7.8 per cycle; intravenous antibiotics: $637.8 per cycle; inhaled bronchodilator: $277.2 per cycle; non-invasive ventilation: $925.8 per cycle; ICU admission: $36,803.0 per cycle.

Abbreviations: RILI, radiation-induced lung injury; APE, acute pulmonary exacerbation; ICU, Intensive Care Unit; MIAB, Medical Insurance Administration Bureau of Guangzhou; AE, adverse event; CT, computed tomography.

^†^ Routine laboratory tests refer to selected examinations, including full blood count, inflammatory markers, blood biochemistry, arterial blood gas analysis, blood culture, and sputum culture. Detailed descriptions of the clinical treatment practice for each health state are provided in the Supplementary Methods.

^‡^ When RILI was accompanied by APE, the corresponding clinical management included all treatment practices listed above for RILI plus those indicated for APE, with any overlapping items counted only once. Please see the Supplementary Methods for the full descriptions of the clinical treatment practice for each health state.

^§^ Includes the costs of transportation, accommodation, and additional meal during follow-up.

^¶^ Refers to wage loss related to patients' and caregivers' time off work.

**Table B.** **The CHEERS 2022 Checklist**

| **Section/topic** | **Item No** | **Guidance for reporting** | **Reported in section** |
| --- | --- | --- | --- |
| **TITLE** | | | |
| Title | 1 | Identify the study as an economic evaluation and specify the interventions being compared. | Title Section |
| **ABSTRACT** | | | |
| Abstract | 2 | Provide a structured summary that highlights context, key methods, results, and alternative analyses. | Abstract Section |
| **INTRODUCTION** | | | |
| Background and objectives. | 3 | Give the context for the study, the study question, and its practical relevance for decision making in policy or practice. | Section 1, paragraph 1-3 |
| **METHODS** | | | |
| Health economic analysis plan | 4 | Indicate whether a health economic analysis plan was developed and where available. | NA |
| Study population | 5 | Describe characteristics of the study population (such as age range, demographics, socioeconomic, or clinical characteristics). | Section 2, paragraph 1 |
| Setting and location | 6 | Provide relevant contextual information that may influence findings. | Section 2, paragraph 1 |
| Comparators | 7 | Describe the interventions or strategies being compared and why chosen. | Section 2, paragraph 1-2 |
| Perspective | 8 | State the perspective(s) adopted by the study and why chosen. | Section 2, paragraph 6 |
| Time horizon | 9 | State the time horizon for the study and why appropriate. | Section 2, paragraph 1-2 |
| Discount rate | 10 | Report the discount rate(s) and reason chosen. | Section 2, paragraph 2 |
| Selection of outcomes | 11 | Describe what outcomes were used as the measure(s) of benefit(s) and harm(s). | Section 2, paragraph 3 |
| Measurement of outcomes | 12 | Describe how outcomes used to capture benefit(s) and harm(s) were measured. | Section 2, paragraph 3; Supplementary Methods |
| Valuation of outcomes | 13 | Describe the population and methods used to measure and value outcomes. | Section 2, paragraph 1, 7 |
| Measurement and valuation of resources and costs | 14 | Describe how costs were valued. | Section 2, paragraph 6;  Supplementary Methods |
| Currency, price date, and conversion | 15 | Report the dates of the estimated resource quantities and unit costs, plus the currency and year of conversion. | Section 2, paragraph 6 |
| Rationale and description of model | 16 | If modeling is used, describe in detail and why used. Report if the model is publicly available and where it can be accessed. | Section 2, paragraph 1, 2 |
| Analytics and assumptions | 17 | Describe any methods for analysing or statistically transforming data, any extrapolation methods, and approaches for validating any model used. | Section 2, paragraph 2;  Supplementary Methods |
| Characterising heterogeneity | 18 | Describe any methods used for estimating how the results of the study vary for subgroups. | NA |
| Characterising distributional effects | 19 | Describe how impacts are distributed across different individuals or adjustments made to reflect priority populations. | NA |
| Characterising uncertainty | 20 | Describe methods to characterise any sources of uncertainty in the analysis. | Section 2, paragraph 2; Table S1 |
| Approach to engagement with patients and others affected by the study | 21 | Describe any approaches to engage patients or service recipients, the general public, communities, or stakeholders (such as clinicians or payers) in the design of the study. | NA |
| **RESULTS** | | | |
| Study parameters | 22 | Report all analytic inputs (such as values, ranges, references) including uncertainty or distributional assumptions. | Section 2,  paragraph 2, 6, 7 |
| Summary of main results | 23 | Report the mean values for the main categories of costs and outcomes of interest and summarise them in the most appropriate overall measure. | Section 3, paragraph 2 |
| Effect of uncertainty | 24 | Describe how uncertainty about analytic judgments, inputs, or projections affect findings. Report the effect of choice of discount rate and time horizon, if applicable. | Section 3, paragraph 3-6 |
| Effect of engagement with patients and others affected by the study | 25 | Report on any difference patient/service recipient, general public, community, or stakeholder involvement made to the approach or findings of the study. | NA |
| **DISCUSSION** | | | |
| Study findings, limitations, generalisability, and current knowledge | 26 | Report key findings, limitations, ethical or equity considerations not captured, and how these could affect patients, policy, or practice. | Section 4,  paragraph 1, 8, 9 |
| **OTHER RELEVANT INFORMATION** | | | |
| Source of funding | 27 | Describe how the study was funded and any role of the funder in the identification, design, conduct, and reporting of the analysis. | Funding Section |
| Conflicts of interest | 28 | Report authors conflicts of interest according to journal or international Committee of Medical journal Editors requirements. | Conflict of interest Section |

From: Husereau D, Drummond M, Augustovski F, de Bekker-Grob E, Briggs AH, Carswell C, et al. Consolidated Health Economic Evaluation Reporting Standards 2022 (CHEERS 2022) statement: updated reporting guidance for health economic evaluations. BMJ. 2022 Jan 11;376:e067975. doi: 10.1136/bmj-2021-067975.

**Table C. Model Validation of the Markov Model-Predicted RILI Pattern Compared with the Real-world Observed RILI Pattern**

| **Time Point** | **RILI Pattern**  **(by CTCAE V5.0)** | **Pirfenidone Treatment Group** | | |  | **Standard Treatment Group** | | |
| --- | --- | --- | --- | --- | --- | --- | --- | --- |
|  |  | **Markov Model**  **Prediction (%) ^†^** | **Real-world**  **Observation (%) ^‡^** | ***P*-value^§^** |  | **Markov Model**  **Prediction (%) ^†^** | **Real-world**  **Observation (%) ^‡^** | ***P*-value^§^** |
| Week 4 | No RILI | 4.6 | 5.4 | 0.84 |  | 1.8 | 1.6 | 0.94 |
|  | Grade 1 RILI | 30.6 | 33.9 |  |  | 19.3 | 22.2 |  |
|  | Grade 2 or 3 RILI | 63.9 | 60.7 |  |  | 76.4 | 74.6 |  |
|  | Grade 4 RILI | 0 | 0 |  |  | 0 | 0 |  |
|  | Grade 5 RILI | 0 | 0 |  |  | 1.8 | 1.6 |  |
|  | Death of other causes | 0.8 | 0 |  |  | 0.7 | 0 |  |
| Week 8 | No RILI | 10.3 | 14.3 | 0.83 |  | 3.0 | 3.4 | 0.84 |
|  | Grade 1 RILI | 42.3 | 38.8 |  |  | 27.1 | 30.5 |  |
|  | Grade 2 or 3 RILI | 45.2 | 44.9 |  |  | 65.5 | 64.4 |  |
|  | Grade 4 RILI | 0 | 0 |  |  | 0 | 0 |  |
|  | Grade 5 RILI | 0 | 0 |  |  | 3.0 | 1.7 |  |
|  | Death of other causes | 2.2 | 2.0 |  |  | 1.3 | 0 |  |
| Week 16 | No RILI | 21.0 | 15.0 | 0.83 |  | 4.8 | 5.8 | 0.82 |
|  | Grade 1 RILI | 47.3 | 50.0 |  |  | 32.5 | 25.0 |  |
|  | Grade 2 or 3 RILI | 27.1 | 30.0 |  |  | 55.2 | 61.5 |  |
|  | Grade 4 RILI | 0 | 0 |  |  | 0 | 0 |  |
|  | Grade 5 RILI | 0 | 0 |  |  | 4.9 | 5.8 |  |
|  | Death of other causes | 4.6 | 5.0 |  |  | 2.5 | 1.9 |  |
| Week 24 | No RILI | 29.1 | 27.5 | 0.96 |  | 6.3 | 9.6 | 0.81 |
|  | Grade 1 RILI | 44.7 | 42.5 |  |  | 33.7 | 36.5 |  |
|  | Grade 2 or 3 RILI | 19.6 | 22.5 |  |  | 50.0 | 46.2 |  |
|  | Grade 4 RILI | 0 | 0 |  |  | 0 | 0 |  |
|  | Grade 5 RILI | 0 | 0 |  |  | 6.5 | 5.8 |  |
|  | Death of other causes | 6.6 | 7.5 |  |  | 3.6 | 1.9 |  |

Abbreviations: RILI, radiation-induced lung injury.
^†^ The Markov model-predicted RILI pattern presents the distribution of RILI grades simulated at weeks 4, 8, 16, and 24.
^‡^ The real-world observed RILI pattern was derived from the phase II RCT cohort data at the four surveillance time points (weeks 4, 8, 16, and 24).
^§^ The *P*-value was calculated using the Chi-square test.

**Table D. Distribution of Cancer Types at Baseline in the Phase II RCT (mITT Population)**

| Cancer type | Pirfenidone group (N=60) | Control group (N=63) |
| --- | --- | --- |
| NSCLC | 32 (53.3) | 38 (60.3) |
| SCLC | 10 (16.7) | 9 (14.3) |
| Esophageal cancer | 15 (25.0) | 10 (15.9) |
| Others | 3 (5.0) | 6 (9.5) |

Abbreviations: mITT: modified intention to treat; NSCLC, None-small cell lung cancer; SCLC, Small cell lung cancer.

**Table E. AE-related clinical scenario cost-effectiveness analysis**

| **Treatment strategy** | **Total values** | | **Incremental values** | | **ICER**  **($/QALY)** | **NHB (QALY)** |
| --- | --- | --- | --- | --- | --- | --- |
|  | **Cost ($)** | **Effectiveness (QALY)** | **Cost ($)** | **Effectiveness (QALY)** |  |  |
| Glucocorticoids | 4,438 | 0.242 | —— | —— | —— | 0.198 |
| Pirfenidone plus glucocorticoids | 5,047 | 0.269 | 609 | 0.027 | 22,715 | 0.219 |

Abbreviations: AE, adverse event; ICER, incremental cost-effectiveness ratio; QALY, quality-adjusted life-year; NHB, net health benefit

**Table F**．**Summary of Pirfenidone Unit Prices Across Different Countries**

| Country | Price ($ / tablet) | Country | Price ($ / tablet) |
| --- | --- | --- | --- |
| Australia | 0.38-3.00 | United Kingdom | 0.38-3.00 |
| Canada | 0.38-3.00 | India | 0.38-1.57 |
| Mauritius | 0.38-3.00 | United States | 0.38-1.40 |
| New Zealand | 0.38-3.00 |  |  |

Note: All prices refer to pirfenidone tablets with a strength of 200 mg per tablet.

 **Fig A. Validation of the Markov Model.** The figure demonstrates how the Markov model simulates the 24-week acute pulmonary exacerbation-free survival patterns: (A) pirfenidone plus glucocorticoids (yellow line), (B) glucocorticoids alone (red line) compared with the real-world data (blue lines). The shaded areas represent the 95% confidence intervals of the Kaplan-Meier estimates. Model-simulated survival curves were generated from the Markov model with the base-case model parameters. The P value was calculated using the Log-rank test.

**Fig B. Tornado Diagram of One-Way Sensitivity Analysis.** The figure depicts the influence of varying all parameters within their predefined ranges on the ICERs between pirfenidone plus glucocorticoids and glucocorticoids alone in patients with grade 2 or 3 RILI. For presentation clarity, the parameters associated with (A) the pirfenidone plus glucocorticoids strategy and (B) the standard glucocorticoid strategy are displayed in separate panels. Blue bars and yellow bars illustrate the ICERs that are greater and less than the base-case ICERs, respectively. Red bars indicate that the ICERs go across the WTP threshold, leading to a switch of the most cost-effective strategy. Numbers on either side of the bars indicate the range of values used for each parameter in the sensitivity analysis. Solid and dashed lines represent the ICERs in the base-case analysis and the WTP threshold of $100,000 per QALY, respectively.

Abbreviations: ICU, intensive care unit; APE, acute pulmonary exacerbation; APEFS, acute pulmonary exacerbation free survival; RILI, radiation-induced lung injury; ICER, incremental cost-effectiveness ratio; WTP, willingness-to-pay; QALY, quality-adjusted life-year.

 **Fig C. Two-Way Sensitivity Analysis of Disease Progression-related Parameters.** The blue area indicates parameter combinations for which pirfenidone plus glucocorticoids is the more cost-effective strategy, whereas the yellow area indicates combinations for which standard glucocorticoid therapy is the more cost-effective strategy, at the WTP threshold of $100,000 per QALY.

Abbreviations: RILI, radiation-induced lung injury; APEFS, acute pulmonary exacerbation-free survival; APE, acute pulmonary exacerbation; ICU, intensive care unit.

 **Fig D. Scatter plot of probabilistic sensitivity analysis for AE-related clinical scenario analysis.** Black line indicates threshold of WTP of $100,000 per QALY. Each point in scatterplot corresponds to one sample of parameter values. Points above reference line indicate higher incremental cost effectiveness ratio than threshold value. The red ellipse represents the 95% confidence ellipse around the mean incremental cost and incremental effectiveness.

Abbreviations: AE, adverse event; QALY, quality-adjusted life-year; WTP, willingness-to-pay.

 **Fig E. Scatter plot of probabilistic sensitivity analysis at the China-specific WTP threshold.** Black line indicates threshold of WTP of $27,680 per QALY. Each point in scatterplot corresponds to one sample of parameter values. Points above reference line indicate higher incremental cost effectiveness ratio than threshold value. The red ellipse represents the 95% confidence ellipse around the mean incremental cost and incremental effectiveness.

Abbreviations: QALY, quality-adjusted life-year; WTP, willingness-to-pay.
