# Supplementary material for: Cost-effectiveness of pirfenidone for patients with cancer and grade 2 or grade 3 radiation-induced lung injury: A modeling study
Source: PLoS Med. 2026 Aug 5;23(8):e1004817. doi: 10.1371/journal.pmed.1004817 (PMC13440887; doi:10.1371/journal.pmed.1004817)
Supplement: Supplementary file 2 [file pmed.1004817.s002.docx]

**S2 Appendix. Supplementary Methods**

**1. Transition Probabilities Estimation**

Transition probabilities between radiation-induced pulmonary injury (RILI) grades were derived from individual-level data collected at five scheduled follow-up visits (baseline, week 4, week 8, week 16, and week 24) in our pirfenidone randomized controlled trial (RCT). For each patient, changes in RILI grade between consecutive follow-up visits were tracked. For each grade (from no RILI to grade 4 RILI), the number of transitions to each possible subsequent grade was counted across all the cohorts and all follow-up intervals. The transition probability from an initial grade *i* to a subsequent grade *j* was then calculated as the proportion of transitions from grade *i* that resulted in grade *j* at the next surveillance, relative to the total number of patients starting at grade *i*. This approach allowed estimation of RILI grade-specific transition probabilities, which were subsequently used in our Markov model.

The transition probabilities related to acute pulmonary exacerbation (APE) encompass occurrence, severity grading, and subsequent outcomes, were derived from our RCT data. For each RILI grade, the probability of developing APE at each follow-up was calculated as the proportion of patients experiencing APE among all patients within that RILI grade. Our RCT graded APE severity according to the Common Terminology Criteria for Adverse Events version 5.0 (CTCAE v5.0[1]): grade 1 (mild), grade 2 (moderate), grade 3 (severe), grade 4 (life-threatening), and grade 5 (death). Considering the substantial differences in cost and health impact between mild and severe APE, grades 1–2 were categorized as mild APE status, and grades 3–4, typically manifested as hypoxemia, were defined as severe APE status.[2, 3] For the subsequent Markov model construction and simulation, the proportions of mild and severe APE among APE patients were calculated, enabling the refinement of transition probabilities according to APE severity. Follow-up data also provided information on APE outcomes, including symptom recovery, improvement, persistence, and death, with transition probabilities calculated as the proportion of patients experiencing each outcome among all APE cases. All these transition probabilities enabled a more comprehensive and realistic simulation of the APE module within our Markov model.

The transition probabilities for pirfenidone-related adverse events (AE) per Markov cycle were calculated based on individual-level data from our RCT. AE severity was graded according to the CTCAE v5.0[1]: grade 1 (mild), grade 2 (moderate), grade 3 (severe), grade 4 (life-threatening), and grade 5 (death). Given the substantial differences in cost and health impact across severity levels and in line with prior literature[7, 8], we combined grades 1-2 as mild AEs and grades 3-4 as severe AEs. Notably, the observed average time to resolution of AEs aligned well with the length of a Markov cycle. Therefore, we estimated the per-cycle probability of mild and severe AEs as the proportion of cycles in which AEs occurred, calculated as the total number of cycles with an event divided by the total number of cycles accrued across all patients. Accordingly, we refined the pirfenidone-related AE module within the Markov model for the clinical scenario analysis.

Additionally, two specific transition probabilities warrant further explanation. The first concerns the probabilities of mild and severe APE among patients with no RILI, for which no empirical data were available due to absence of APE events. For the pirfenidone group, considering that the probability of developing mild APE among grade 1 RILI patients was 100% and that APE severity tends to increase with worsening baseline pulmonary status,[4] we assigned a probability of 100% for mild APE and 0% for severe APE in no-RILI patients. For the glucocorticoids group, probabilities of mild and severe APE for no-RILI patients were estimated by linear extrapolation from the observed probabilities in patients with grade 1 and grades 2–3 RILI, reflecting the trend of increasing APE severity with higher RILI grades.[4] This approach allowed a reasonable estimation of APE severity distribution in patients without RILI and ensured consistency across treatment groups. The second concerns the transition probabilities related to grade 4 RILI. Due to the rarity of grade 4 RILI in clinical practice, no corresponding events were observed in our RCT cohort. To estimate the probabilities of grade 4 RILI transitioning to other RILI grades or developing APE, we applied a linear fitting and extrapolation approach. Specifically, using the observed transition data from patients with no RILI, grade 1 RILI, and grade 2–3 RILI, we performed a least-squares linear regression to obtain initial estimates for grade 4 RILI. These estimates were then normalized to ensure the transition probabilities summed to one. To account for potential uncertainty introduced by the extrapolated values mentioned above, all parameters derived from extrapolation were further tested in sensitivity analyses with ±20% variation.

**2. Health Utility Value**

The main health states in our Markov model included RILI and APE, with utility values derived from published literature. Specifically, the utility for patients with no RILI was assigned based on post-treatment data from non-small cell lung cancer (NSCLC) patients, who accounted for the largest proportion (56.9%) of participants in our RCT. Grade 1 RILI was assigned a utility value according to previously reported values.[5] Due to the scarcity of published data for grades 2/3 and grade 4 RILI, we estimated their values using those reported for moderate and severe infectious pneumonia, respectively, based on clinical similarity in symptom severity and disease burden.[6] Additionally, the assignment of utility values for APE warrants further clarification. Unlike previous studies summarizing APE-related disutility using a single value without distinguishing severity,[7] our model assigned disutility values separately according to severity. Specifically, disutility for mild APE was derived from patient-reported clinical symptoms recorded during RCT surveillance, such as cough and dyspnea, whereas disutility for severe APE was based on estimates from the published literature.[7] Collectively, the stratified assignment ensured that the model accurately reflected variations in health-related quality of life across different RILI severities and APE occurrences.

Given the scarcity of published data on health utility decrements specific to AEs stratified by severity, and the absence of quality-of-life assessments during RCT follow-up, we derived utility decrements for mild and severe AEs from previously published studies whose AE profiles closely resembled that of our trial. To address potential bias inherent in relying on external data sources, we systematically evaluated the parameters through one-way sensitivity analysis to examine how its variation might affect the robustness of our cost-effectiveness conclusions.

**3. Treatment Regimen**

All patients received medication consistent with our RCT protocol, with the pirfenidone group treated with pirfenidone plus prednisone and the glucocorticoids group treated with prednisone alone. Other treatment for each 4-week cycle was defined according to the severity of RILI and the presence of APE based on relevant international clinical guidelines for the management of RILI and APE,[8-10] as detailed below:

For RILI without APE, patients with no RILI or grade 1 RILI required no additional treatment. Grade 2 cases received symptomatic treatment only. Grade 3 RILI was managed with symptomatic treatment in combination with oral antibiotics, intravenous glucocorticoids, and low-flow oxygen therapy. Patients with grade 4 RILI required more intensive measures, including symptomatic treatment combined with intravenous antibiotics, intravenous glucocorticoids, mechanical ventilation, and bronchodilators.

For RILI with mild APE, patients with no RILI, grade 1, or grade 2 RILI were managed with symptomatic treatment and oral antibiotics. Grade 3 cases received symptomatic treatment plus oral antibiotics, intravenous glucocorticoids, low-flow oxygen therapy, and bronchodilators. Grade 4 RILI accompanied by mild APE was treated comprehensively, including symptomatic treatment combined with intravenous antibiotics, intravenous glucocorticoids, mechanical ventilation, and bronchodilators.

For RILI with severe APE, patients with no RILI, grade 1 and grade 2 RILI received symptomatic treatment with intravenous antibiotics, intravenous glucocorticoids, and high-flow oxygen therapy. Grade 3 received the same treatment with the addition of bronchodilators, Grade 3 patients with unrelieved severe symptoms, as well as grade 4 cases, were admitted to the intensive care unit (ICU) for closer monitoring and advanced respiratory support.

**4. Clinical Examination**

In accordance with international clinical guidelines for the management of RILI and APE, the routine diagnostic and monitoring tests were standardized across different RILI grades and APE severities.[10-12]

For RILI without APE, patients with no RILI did not require routine examinations. Those with grade 1 RILI underwent a full blood count (FBC), sputum culture, and pulmonary function test (PFT) at initial presentation. Grade 2 RILI patients received baseline evaluations consisting of non-contrast chest CT, FBC, arterial blood gas (ABG) analysis, inflammatory biomarkers, blood biochemistry, sputum culture, bronchoscopy, and PFT, followed by weekly blood tests and chest X-rays. Grades 3 and 4 RILI underwent the same tests as grade 2, with the addition of blood culture.

For RILI accompanied by mild APE, patients with no RILI were evaluated with chest X-ray, FBC, blood biochemistry, and inflammatory biomarkers. Grade 1 RILI required additional sputum culture and PFT. For grade 2, grade 3, and grade 4 RILI, the routine follow-up examinations were consistent between patients with or without mild APE at the same grade, respectively.

For RILI accompanied by severe APE, patients with no RILI underwent baseline assessments including chest X-ray, FBC, blood biochemistry, inflammatory biomarkers, ABG analysis, sputum and blood cultures, and bronchoscopy, followed by reassessment of hematologic and inflammatory parameters once per model cycle. Grade 1 RILI patients underwent the same tests, with the addition of PFT. For grades 2 and 3 RILI, initial evaluations consisted of non-contrast chest CT, FBC, ABG, inflammatory biomarkers, blood biochemistry, sputum and blood cultures, bronchoscopy, and PFT, with Inflammatory parameters reassessed once per cycle, and weekly blood tests and chest X-rays performed. Grade 3 RILI patients with unrelieved severe symptoms, as well as all grade 4 cases, were admitted to ICU for advanced management.

**5. Literature Search**

It is worth noting that certain parameters were obtained from previously published studies. Specifically, these parameters included the sensitivity analysis ranges for rare clinical scenarios such as grades 4 and 5 RILI, as well as parameters that are difficult to estimate in real-world settings, including ICU costs, direct non-health care costs, indirect costs, and utility estimates. To identify relevant data, we conducted a comprehensive search of PubMed and EMBASE covering the period from January 1990 to October 2025, without any language restrictions. Evidence from systematic reviews or meta-analyses was prioritized as the highest-quality source, followed by data from randomized controlled trials and retrospective studies. When multiple eligible references were available for the same parameter, studies with larger sample sizes were considered more reliable.

6. **Reference**

1.	U.S. DEPARTMENT OF HEALTH AND HUMAN SERVICES. National Institutes of Health National Institutes of Health. Common Terminology Criteria for Adverse Events (CTCAE) Version 5.0 Published: 2017. ;<https://ctep.cancer.gov/protocoldevelopment/electronic_applications/docs/CTCAE_v5_Quick_Reference_8.5x11.pdf> (Accessed on July 25, 2025).

2.	Kobayashi H, Naito T, Omae K, Omori S, Nakashima K, Wakuda K, et al. Impact of interstitial lung disease classification on the development of acute exacerbation of interstitial lung disease and prognosis in patients with stage III non-small-cell lung cancer and interstitial lung disease treated with chemoradiotherapy. Journal of Cancer. 2018;9(11):2054.

3.	Loveman E, Copley VR, Copley V, Colquitt J, Scott DA, Scott D, et al. The clinical and cost effectiveness of treatments for idiopathic pulmonary fibrosis: a systematic review and economic evaluation. Health Technology Assessment. 2015;19(20):1-336.

4.	Kreuter M, Wuyts WA, Wijsenbeek M, Bajwah S, Maher TM, Stowasser S, et al. Health-related quality of life and symptoms in patients with IPF treated with nintedanib: analyses of patient-reported outcomes from the INPULSIS® trials. Respiratory research. 2020;21(1):36.

5.	Sawada T, Kondo M, Goto M, Murakami M, Ishida T, Hiroshima Y, et al. Cost-utility analysis of proton beam therapy for locally advanced esophageal cancer in Japan. PloS one. 2024;19(9):e0308961.

6.	Butler JR, McIntyre P, MacIntyre CR, Gilmour R, Howarth AL, Sander B. The cost-effectiveness of pneumococcal conjugate vaccination in Australia. Vaccine. 2004;22(9-10):1138-49.

7.	Loveman E, Copley VR, Colquitt JL, Scott DA, Clegg AJ, Jones J, et al. The effectiveness and cost-effectiveness of treatments for idiopathic pulmonary fibrosis: systematic review, network meta-analysis and health economic evaluation. BMC Pharmacology and Toxicology. 2014;15(1):63.

8.	De Ruysscher D, Wauters E, Jendrossek V, Filippi AR, Revel M-P, Faivre-Finn C, et al. Diagnosis and treatment of radiation induced pneumonitis in patients with lung cancer: An ESTRO clinical practice guideline. Radiotherapy and Oncology. 2025;207:110837.

9.	Arroyo-Hernández M, Maldonado F, Lozano-Ruiz F, Muñoz-Montaño W, Nuñez-Baez M, Arrieta O. Radiation-induced lung injury: current evidence. BMC pulmonary medicine. 2021;21(1):9.

10.	Raghu G, Remy-Jardin M, Richeldi L, Thomson CC, Inoue Y, Johkoh T, et al. Idiopathic pulmonary fibrosis (an update) and progressive pulmonary fibrosis in adults: an official ATS/ERS/JRS/ALAT clinical practice guideline. American journal of respiratory and critical care medicine. 2022;205(9):e18-e47.

11.	National Institute for Health and Care Excellence. Pneumonia: diagnosis and management (NICE guideline NG250). 2025;<https://www.nice.org.uk/guidance/ng250> (Accessed on October 12, 2025).

12.	Hozumi H, Hasegawa H, Miyashita K, Yasui H, Suzuki Y, Kono M, et al. Efficacy of corticosteroid and intravenous cyclophosphamide in acute exacerbation of idiopathic pulmonary fibrosis: A propensity score‐matched analysis. Respirology. 2019;24(8):792-8.
