# Supplementary material for: Cost-effectiveness of pirfenidone for patients with cancer and grade 2 or grade 3 radiation-induced lung injury: A modeling study
Source: PLoS Med. 2026 Aug 5;23(8):e1004817. doi: 10.1371/journal.pmed.1004817 (PMC13440887; doi:10.1371/journal.pmed.1004817)
Supplement: Supplementary file 4 [file pmed.1004817.s006.docx]

Dear professor Tosun,

Thank you for the opportunity to revise our manuscript “***Cost-Effectiveness Analysis of Pirfenidone for Cancer Patients with Grade 2 and Grade 3 Radiation-Induced Lung Injury* (PMEDICINE-D-25-03853R1)**”. We sincerely thank the reviewers for their time and insightful comments, which have significantly strengthened this work. Our point-by-point responses are provided below. Text has been added/modified, and all changes are indicated in the manuscript, with corresponding locations provided.

We thank the editorial office for the insightful comments and overall positive feedback on our work. We sincerely hope the revised manuscript, which is strengthened by the additional analyses, is now suitable for publication in ***PLOS Medicine*** and look forward to hearing from you.

Yours Sincerely,

Ming Chen, M.D.

Department of Radiation Oncology, Sun Yat-sen University Cancer Center, State Key Laboratory of Oncology in South China, Collaborative Innovation Center of Cancer Medicine. No.651, Dongfeng East Road, Guangzhou, Guangdong, 510060, P.R. China

Tel: 86-20-87340540

E-mail: [chenming@sysucc.org.cn](mailto:chenming@sysucc.org.cn)

**POINT-TO-POINT RESPONSE**

**Reviewer #1**

**Thanks authors for their great effort to improve the manuscript. The authors have addressed my comments well in a professional way. I am satisfied with the response and revision. No further issues needing attention.**

**Response:** We greatly appreciate your encouraging and supportive comments. It is our honor to receive your recognition of our work. Thank you very much for your time and expertise in reviewing our manuscript.

**Reviewer #2**

**I thank the authors for their careful responses to the previous comments and for the substantial revisions made to the manuscript. The revised version has improved in terms of model transparency, discussion of generalizability, and the wording of the conclusions. Overall, this study addresses an important clinical and health economic question by evaluating the economic value of adding pirfenidone to glucocorticoids for the treatment of grade 2 or 3 radiation-induced lung injury (RILI), based on a phase II randomized controlled trial. However, several methodological and reporting issues still require further clarification.**

**Comment 1. The estimation of utility values and the simplification of health states require further discussion. Because the number of patients with grade 3 radiation pneumonitis (RP) was limited, the authors combined grade 2 and grade 3 RP into a single health state to improve the statistical robustness of the model parameters. This approach is understandable from a statistical perspective. However, from clinical and economic perspectives, grade 2 and grade 3 RP may differ substantially in respiratory support, medications for infection, healthcare resource utilization, and utility decrement. For example, patients with grade 2 RP may usually receive low-flow oxygen support and empirical antibiotics when infection is suspected, whereas patients with grade 3 RP may require high-flow oxygen therapy or non-invasive ventilation, as well as more intensive anti-infective and anti-inflammatory management. Therefore, combining these two grades may obscure clinically meaningful heterogeneity
and potentially underestimate the cost and utility burden among patients with grade 3 RP. The authors should discuss the potential impact of this assumption and, if feasible, perform a clinical scenario analysis assigning higher costs and larger utility decrements to grade 3 RP.**

**Response:** We sincerely appreciate the reviewer’s insightful comments regarding the potential impact of clinical and economic differences between grade 2 and grade 3 radiation-induced lung injury (RILI) on our cost-effectiveness conclusions. We accounted for these differences in healthcare resource use and utility decrements during parameter estimation, and we would like to provide the following clarifications.

Regarding healthcare resource use, we strictly followed RILI clinical guidelines to estimate the per-cycle treatment and follow-up costs for grade 2 and grade 3 RILI patients, respectively (***please see Additional file: Supplementary Methods for detailed protocols***). We then calculated the weighted cost for the combined “grade 2 or 3 RILI” health state based on the actual proportions of grade 2 and grade 3 patients observed in our randomized controlled trial (RCT), rather than simply applying the cost of either grade alone. To further address the reviewer’s concern, we performed a one-way sensitivity analysis on the proportion of grade 3 RILI, given its potential variation in clinical practice. The results showed that when the proportion of grade 3 RILI in the pirfenidone group varied between 1.9% and 2.9%, the ICER ranged from $20,534 per QALY to $21,378 per QALY. When the proportion of grade 3 RILI in the glucocorticoid group varied between 3.6% and 5.4%, the ICER decreased from $22,252 per QALY to $19,661 per QALY. Notably, pirfenidone would only lose cost-effectiveness when the proportion of grade 3 RILI in its group reached 93.2%. For the glucocorticoids group, the ICER never exceeded $27,435 per QALY regardless of the proportion. Given that the actual incidence of grade 3 RILI does not exceed 16% in clinical practice[1], these findings indicate that real-world differences in the proportions of grade 2 and grade 3 RILI do not pose a substantial challenge to the cost-effectiveness conclusion.

Regarding health utility values, specific utility values for grade 2 and grade 3 RILI are limited in current literature. Therefore, it is not feasible to quantify utility decrements separately for each grade. To address the reviewer’s concern, we performed a one-way sensitivity analysis on the utility decrements of the combined “grade 2 or 3 RILI” health state to explore whether variations in utility driven by different proportions of grade 3 RILI could influence our cost-effectiveness conclusion. The results showed that when this utility decrement varied between 0.024 and 0.032, the ICER decreased from $24,144 to $18,512 per QALY. Notably, even under the extreme condition where this utility decrement was set to 0, pirfenidone’s ICER consistently remained below $61,659 per QALY. This indicates that variations in the utility decrement of “grade 2 or 3 RILI” health state are unlikely to change the cost-effectiveness conclusions of pirfenidone.

Taken together, we confirm that the combination of grade 2 and grade 3 RILI into a single health state is unlikely to reverse our cost-effectiveness conclusions. Moreover, we agree with the reviewer that it is necessary to emphasize the clinical and economic differences between grade 2 and grade 3 RILI. We have added the above content to a **Clinical Scenario Analysis** of our manuscript ***(please see the Results section, line 386-407 on page 20-21).***

**In addition, treatment-related toxicity was not incorporated into the model because the incidence of grade 3 or higher adverse events was comparable between groups. However, QALY estimates may also be affected by lower-grade adverse events. Pirfenidone-related gastrointestinal discomfort, rash, and other adverse events may be classified as low-grade but may persist over time and affect patients' quality of life and treatment adherence. The authors should further clarify why only grade 3 or higher adverse events were considered relevant sources of treatment-related disutility and consider incorporating treatment-related disutility in sensitivity or scenario analyses to evaluate the impact of this assumption on QALY and ICER results.**

**Response:** We also thank the reviewer for this helpful comments. Regarding why only grade 3 or higher adverse events were considered relevant sources of treatment-related disutility, we would like to provide the following explanations.

First, published studies have shown that the incorporation of treatment-related adverse events in health economic analysis is primarily guided by the severity of the events[2]. Grade 1-2 adverse events tend to be excluded as clinically insignificant events[3]. Some published reviews have recommended focusing on grade 3 or higher adverse events while excluding grade 1-2 events in health economic analyses[2, 4, 5]. Therefore, fewer than 11% of the health economic studies have incorporated utility decrements for grade 1-2 adverse events[2, 3]. Second, enrolled patients of our RCT were thoracic cancer patients with RILI, whose health utility values are significantly lower than those of the general population. Compared with the great utility decrements caused by RILI and tumor burden, the utility decrement caused by grade 1-2 adverse events is limited[2]. Moreover, clinical guidelines define grade 1-2 adverse events as non-hospitalized events requiring only medication or observation, with consequently lower treatment costs. Therefore, the impact of a single grade 1-2 adverse event on cost-effectiveness is minimal. Third, whether to incorporate treatment-related adverse events also depends on the between-group difference in adverse event incidence[2]. In our RCT, the incidence of adverse events was remarkably similar between two treatment arms: the incidence of grade 1-2 events was 66% in the pirfenidone group versus 61% in the control group, and that of grade 3-5 events was 15% versus 14%, respectively. Notably, the distribution of adverse event incidence across severity grades was comparable between the two groups as well[6]. Therefore, the identical AE-related model framework and similar transition probabilities between the two groups render the impact on the overall cost-effectiveness minimal. Fourth, our RCT results showed that only four patients (approximately 6.0%) discontinued pirfenidone due to treatment-related adverse events (including grade 1-4 events) during the follow-up, including two temporary interruptions and two permanent withdrawals). This suggests that adverse events caused by pirfenidone regimen did not substantially affect treatment adherence.

Therefore, we did not incorporate any treatment-related toxicities, neither grade 1-2 nor grade 3-4 adverse effects, into our cost effectiveness model.

However, we fully acknowledge the thoughtfulness of the reviewer’s comment. Given that our RCT and our cost-effectiveness analysis focused adding pirfenidone into the standard glucocorticoids regimen, explicitly accounting for the potential impact of pirfenidone-related adverse events (including both grade 1-2 and grade 3 or higher events) on cost-effectiveness would strengthen the robustness of our conclusions. Based on individual-level data of our RCT, 26 patients experienced pirfenidone-related grade 1-2 adverse events (42 events in total), and 4 patients suffered from grade 3 pirfenidone-related adverse events (5 events in total). No drug-related grade 4 or 5 events were observed. Grade 1-2 adverse events included abnormal liver function, hypoalbuminemia, nausea, fatigue, decreased appetite, dizziness, anemia, weight loss, pulmonary embolism, abdominal pain, abdominal distension, lower limb edema, lower limb pain, throat pain, rash, thrombocytopenia, gastroesophageal reflux, and hand‑foot syndrome. Grade 3 adverse events included abnormal liver function, rash, and pneumonia.

Considering lack of quality-of-life assessment during the RCT follow-up, we derived utility decrements from published literature reporting similar adverse event profiles to our RCT: a disutility value of 0.014 for grade 1-2 events and 0.157 for grade 3-4 events[7, 8]. Treatment costs were calculated as mean values based on actual treatment regimens: $11.2 per cycle for grade 1-2 events and $199.9 per cycle for grade 3-4 events. Transition probabilities were estimated as the proportion of patients experiencing adverse events per Markov cycle: 13.9% for grade 1-2 events and 1.7% for grade 3-4 events. All relevant parameters have been added to **Additional file: Table S1 *(please see the Table of AE-related parameters below).***

The base-case analysis showed that the pirfenidone plus glucocorticoids regimen yielded an additional 0.027 QALYs at an incremental cost of $609, resulting in an ICER of $22,715 per QALY. This falls well below the $100,000 per QALY WTP threshold, indicating favorable cost-effectiveness ***(The results have been added to Additional file: Table S5, please see the Table of AE-related clinical scenario cost-effectiveness analysis below).*** Further one-way sensitivity analysis on all adverse event-related parameters demonstrated that pirfenidone regimen consistently remained cost-effective across a ±20% variation in these parameters. Specifically, the highest ICER for pirfenidone of $22,943 per QALY occurred when the per-cycle incidence of grade 3-4 adverse events reached 2.0%. Moreover, PSA similarly indicated that pirfenidone was cost-effective in approximately 99.94% of simulations, with 56.87% of simulated samples falling in the northeast quadrant and 43.13% in the southeast quadrant. **The results have been added to Additional file: Fig S4 *(please see the Scatter plot of probabilistic sensitivity analysis for AE-related clinical scenario analysis).***

Taken together, these results suggest that pirfenidone-related adverse events have a minimal impact on the cost-effectiveness of pirfenidone, insufficient to challenge our conclusions. Above analyses have been added to the **Clinical Scenario Analysis section** ***(please see the Results section, line 408-427 on page 21-22).*** We thank the reviewer again for this valuable suggestion.

Table of AE-related parameters

| **Parameter** | **Pirfenidone plus glucocorticoids** | | **Glucocorticoids alone** | | **Distribution** | **Reference** |
| --- | --- | --- | --- | --- | --- | --- |
|  | **Base-Case Estimate** | **Range** | **Base-Case Estimate** | **Range** |  |  |
| Costs for grade 1-2 AEs | 11.2 | 9-13 | — | — | Gamma | MIAB |
| Costs for grade 3-4 AEs  s | 199.9 | 160-240 | — | — | Gamma | MIAB |
| Per-cycle incidence of grade 1-2 AEs | 0.139 | 0.11-0.17 | — | — | Beta | Trial cohort |
| Per-cycle incidence of grade 3-4 AEs | 0.017 | 0.014-0.020 | — | — | Beta | Trial cohort |
| Disutility of grade 1-2 AEs | 0.014 | 0.011-0.017 | — | — | Beta | [7, 8] |
| Disutility of grade 3-4 AEs | 0.157 | 0.13-0.19 | — | — | Beta | [7, 8] |

Abbreviations: AE, adverse event; MIAB, Medical Insurance Administration Bureau of Guangzhou

**Table of AE-related clinical scenario cost-effectiveness analysis**

| **Treatment strategy** | **Total values** | | **Incremental values** | | **ICER ($/QALY)** | **NHB (QALY)** |
| --- | --- | --- | --- | --- | --- | --- |
|  | **Cost ($)** | **Effectiveness (QALY)** | **Cost ($)** | **Effectiveness (QALY)** |  |  |
| Glucocorticoids | 4,438 | 0.242 | —— | —— | —— | 0.198 |
| Pirfenidone plus glucocorticoids | 5,047 | 0.269 | 609 | 0.027 | 22,715 | 0.219 |

Abbreviations: ICER, incremental cost-effectiveness ratio; QALY, quality-adjusted life-year; NHB, net health benefit

 **Scatter plot of probabilistic sensitivity analysis for AE-related clinical scenario analysis.** Black line indicates threshold of WTP of $100,000 per QALY. Each point in scatterplot corresponds to one sample of parameter values. Points above reference line indicate higher incremental cost effectiveness ratio than threshold value. The red ellipse represents the 95% confidence ellipse around the mean incremental cost and incremental effectiveness.

Abbreviations: QALY, quality-adjusted life-year; WTP, willingness-to-pay.

**Comment 2. I appreciate the authors' response to the previous comment regarding the international reference prices of pirfenidone and its generalizability. However, a related issue still requires further clarification. The willingness-to-pay threshold is closely associated with the local economic context, income level, reimbursement system, and healthcare affordability. Therefore, although the supplemented international price comparison helps address the generalizability of drug costs, it does not fully justify the use of $100,000 per QALY as the main decision threshold for a China-based cost-effectiveness analysis.
Moreover, in the Results section, the interpretation of cost-effectiveness remains largely centered on the $100,000 per QALY threshold. Although the cost-effectiveness acceptability curve suggests that pirfenidone becomes more likely to be cost-effective when the WTP threshold exceeds $6,681 per QALY, the authors do not systematically report the probability of being cost-effective under China-relevant WTP thresholds. The authors should provide additional scenario analyses using China-specific thresholds. This would allow readers to better assess whether pirfenidone plus glucocorticoids remains cost-effective within the healthcare and economic context from which the cost data were derived.**

**Response:** We sincerely thank the reviewer for the thoughtful comments regarding the selection of the willingness-to-pay (WTP) threshold. We would like to clarify two points. First, the WTP threshold of $100,000 per QALY is widely accepted in cost-effectiveness analyses[9], including several China-based studies[10, 11]. Second, a unified international WTP threshold facilitates meaningful cross-study comparisons and enhances global translatability of findings. This is why we chose it as our standard threshold.

However, we fully concur with the reviewer that China’s distinctive economic and healthcare environment demands a more context-specific WTP threshold to evaluate whether pirfenidone plus glucocorticoids remains cost-effective. Therefore, we conducted an additional clinical scenario analysis using a China-specific threshold. Following the World Health Organization’s recommendation[12, 13], we selected three times the gross domestic product (GDP) per capita as the decision threshold[14], equivalent to $33,521 per QALY[15]. Notably, the base-case ICER for pirfenidone was $20,956 per QALY, which remained below this China-specific threshold, indicating favorable cost-effectiveness. Furthermore, the probabilistic sensitivity analysis (PSA) based on 10,000 Monte Carlo simulations further reinforced the conclusions, showing that 81.17% of simulated samples fell below the WTP threshold, with 43.24% representing a dominant strategy (more effective and less costly). Taken together, this scenario analysis clearly demonstrates that even under a WTP threshold tailored to the Chinese context, the pirfenidone plus glucocorticoids strategy retains a clear economic advantage over glucocorticoids alone.

We have integrated these findings into the **Clinical Scenario Analysis section** ***(please see the Results section, line 428-442 on page 22-23)***, and presented the PSA results in **Additional File: Fig S5** ***(please see Additional File: Fig S5 below).***

 **Fig S5. Scatter plot of probabilistic sensitivity analysis at a WTP threshold of $33,521 per QALY.** Black line indicates threshold of WTP of $33,521 per QALY. Each point in scatterplot corresponds to one sample of parameter values. Points above reference line indicate higher incremental cost effectiveness ratio than threshold value.

Abbreviations: QALY, quality-adjusted life-year; WTP, willingness-to-pay.

**Comment 3. The Abstract reports that pirfenidone plus glucocorticoids increased costs by $590 compared with glucocorticoids alone, whereas the Base-Case Analysis section reports an incremental cost of $595. The authors should check these values and clarify whether this discrepancy is due to a typographical error or rounding. I also suggest that the authors review the entire manuscript and tables to ensure that there are no similar inconsistencies or misreported data.**

**Response:** We sincerely thank the reviewer for this meticulous review. We acknowledge that in the Base-Case Analysis section, we inadvertently retained an outdated version of the incremental cost data, which led to the inconsistency with the value reported in the Abstract section.

We have now corrected this issue by revising the incremental cost in the Base-Case Analysis section to $590 ***(please see the Results section, line 277 on page 15).*** Furthermore, we have conducted a thorough review of the entire manuscript to confirm that there are no other data inconsistencies or misreported values.

**Comment 4.** **The authors should clarify how immunotherapy exposure and immune-related pneumonitis were handled. Some patients may have received immunotherapy before, during, or after radiotherapy. Immune-related pneumonitis may overlap with RP in both clinical presentation and imaging findings, and may also influence glucocorticoid treatment strategies and related treatment costs. The manuscript does not clearly report immunotherapy use among patients enrolled in the phase II RCT. The authors should report the proportion and balance of immunotherapy exposure between the two treatment groups and explain how RP was distinguished from immune-related pneumonitis. If such data are unavailable, this should be discussed as a limitation, particularly regarding the potential impact of immunotherapy exposure on model results and generalizability.**

**Response:** We greatly appreciate the reviewer for the thoughtful and detailed comments. Regarding the potential impact of immunotherapy exposure on the model results, we have fully considered this issue and believe that CIP would not introduce substantial bias into the results of our cost-effectiveness analysis based on the above considerations.

- First, the RCT on which our cost-effectiveness analysis was based began in 2021, when neoadjuvant or concurrent immunotherapy was neither widely used for thoracic tumors nor supported by clinical guidelines. As a result, only a limited proportion of enrolled patients (approximately 33% in both treatment arms) had received immunotherapy.
- Second, the incidence of checkpoint inhibitor pneumonitis (CIP) is typically below 5% among thoracic carcinoma patients treated with immunotherapy[16]. Given that the proportion of patients receiving immunotherapy was relatively limited, the number of potential CIP cases is negligible.
- Third, the PACIFIC study did not distinct between radiation pneumonitis with or without CIP[17]. Therefore, our RCT chose to remain consistent with the Pacific study in this regard.
- Fourth, according to the treatment guidelines for radiation pneumonitis and CIP[18, 19], the standard treatment regimens and glucocorticoids doses during the inflammatory phase are similar, both primarily involving glucocorticoid-based symptomatic supportive care.

However, given that we have now entered the immunotherapy era, it is necessary to further emphasize the potential impact of immunotherapy exposure on model results. Notably, our ongoing phase III RCT (NCT07388680) has explicitly distinguished between immune-related pneumonitis and radiation pneumonitis for future analyses. We have also further clarified this issue in the **Discussion section** of our manuscript. ***(please see the Discussion section, line 588-594 on page 30).***

**Reviewer #3**

**Comment 1. Regarding Reviewer 3's Comment 5, upon reading the author's more detailed description of the process for comparing model outputs to data, it is not clear that this process involved calibration. It appears no model parameters were tuned to bring model outputs closer to observed data. If the authors agree, they can revert the change that was made. They do not need to refer to this process as "model calibration" as this reviewed had previously, seemingly erroneously suggested.
All other comments were thoroughly addressed. Thank you, it was a pleasure to review this work.**

**Response:** We sincerely thank the reviewer for the clear and insightful distinction between model calibration and validation, as well as for the rigorous review of the methodological description in our manuscript. We would like to clarify that no parameter tuning was performed during the process of comparing model outputs with observed data. Therefore, we fully agree that “model validation” is a more appropriate term to summarize this process.

In our analysis, the transition probability parameters were primarily derived directly from individual-level data collected in the RCT. No subsequent parameter calibration was performed. On this basis, we conducted a direct comparison between the model-simulated outputs and the observed outcomes from the RCT. The purpose of the comparison was to assess the model’s ability to reproduce the RCT results, rather than to calibrate model parameters to bring the output closer to the observed RCT data. This approach is entirely consistent with the reviewer’s observation that no parameter calibration was undertaken.

Therefore, we have revised the manuscript accordingly following the reviewer’s suggestion. Specifically, we have renamed the subsections previously titled “Model Calibration” in the **Methods and Results sections** to “Model Validation” to more accurately reflect the actual procedures performed in this study. ***(please see the Methods section******, line 199-210 on page 11-12; Results section, line 262-270 on page 15).***

**General editorial requests**

**Comment 1. At this stage, we ask that you include a short, non-technical Author Summary of your research to make findings accessible to a wide audience that includes both scientists and non-scientists. The Author Summary should immediately follow the Abstract in your revised manuscript. This text is subject to editorial change and should be distinct from the scientific abstract. Ideally each sub-heading should contain 2-3 single sentence, concise bullet points containing the most salient points from your study. In the final bullet point of ‘What Do These Findings Mean?’ Please include the main limitations of the study in non-technical language.**

**Response:** We thank the editorial office for the guidance regarding the Author Summary. We have prepared a concise, non-technical Author Summary in accordance with PLOS Medicine guidelines. The Author Summary has been inserted immediately following the Abstract in the revised manuscript.

**Comment 2.** **Please confirm that your title complies with PLOS Medicine's style. Your title must be nondeclarative and not a question. It should begin with main concept if possible. "Effect of" should be used only if causality can be inferred, i.e., for an RCT. Please place the study design ("A randomized controlled trial," "A retrospective study," "A modelling study," etc.) in the subtitle (ie, after a colon).**

**Response:** We thank the editorial office for the detailed instructions regarding the journal’s title requirements. We have revised our title to “**Cost-Effectiveness of Pirfenidone for Cancer Patients with Grade 2 and Grade 3 Radiation-Induced Lung Injury: A Modelling Study**” to comply with PLOS Medicine’s style.

**Comment 3. Please confirm that your abstract complies with our requirements, including format (three sections: Background, Methods and Findings, and Conclusions) and providing all the information relevant to this study type. https://journals.plos.org/plosmedicine/s/submission-guidelines#loc-abstract**

**Response:** We sincerely thank the editor for this suggestion. We have revised the abstract to follow the PLOS Medicine structured format, using the headings Background, Methods and Findings, and Conclusions.

**Comment 4. Please ensure that the Introduction ends with a clear description of the study question or hypothesis.**

**Response:** We thank the editor for the suggestion. We confirm that the Introduction ends with a clear description of the study question.

**Comment 5. Please ensure that all abbreviations are defined at first use throughout the text.**

**Response:** We sincerely thank the editor for the reminder. We confirm that all abbreviations of our manuscript are defined at first use throughout the text.

**Comment 6. Please confirm that all numbers presented in the abstract are present and identical to numbers presented in the main manuscript text.**

**Response:** We sincerely thank the editor for this suggestion. We confirm that all numbers presented in the abstract are present and identical to numbers presented in the main manuscript text.

**Comment 7. The funding statement should include: specific grant numbers, initials of authors who received each award, URLs to sponsors’ websites. Also, please state whether any sponsors or funders (other than the named authors) played any role in study design, data collection and analysis, the decision to publish, or preparation of the manuscript. If they had no role in the research, include this sentence: “The funders had no role in study design, data collection and analysis, decision to publish, or preparation of the manuscript.”**

**Response:** We thank the editorial office for the guidance regarding the funding statement. We confirm that our funding statement has been prepared in compliance with the journal's requirements.

**Comment 8. All authors must declare their relevant competing interests per the PLOS policy, which can be seen here: https://journals.plos.org/plosmedicine/s/competing-interests For authors with ties to industry, please indicate whether any of the interests has a financial stake in the results of the current study.**

**Response:** We thank the reviewer for this helpful reminder. We confirm that all other authors have declared that no competing interests exist.

**Comment 9. Please amend your competing interests statement. Funding information should go to the relevant section and include URLs.**

**Response:** We thank the editor for this reminder. We have amended our competing interests statement as requested. Funding information has been moved to the Funding section and includes the required URLs.

**Comment 10. Please review your text for claims of novelty or primacy (e.g. 'for the first time') and remove this language. In addition, please check that any use of statistical terms (such as trend or significant) are supported by the data, and if not please remove them.**

**Response:** We thank the editor for this helpful suggestion. We have reviewed our manuscript and removed all claims of novelty or primacy. We have also checked all statistical terms (such as "trend" or "significant") and confirmed that their use is fully supported by the data.

**Comment 11. Please remove the 'conclusions' subheading from the discussion. Please also remove any other subheadings from the discussion.**

**Response:** We thank the editor for this valuable reminder. We have removed the 'conclusions' subheading and any other subheadings from the discussion.

**Comment 12. Statistical reporting: Please revise throughout the manuscript, including tables and figures.
- Please report statistical information as follows to improve clarity for the reader ""22% (95% CI [13,28]; p</=)"".
- Please separate upper and lower bounds with commas instead of hyphens as the latter can be confused with reporting of negative values.
- Please repeat statistical definitions (HR, CI etc.) for each set of parentheses."**

**Response:** We thank the reviewer for this helpful suggestion. However, this item is not applicable to our study, as the data presented do not require additional 95% confidence intervals or p-value reporting beyond what has already been provided.

**Comment 13. In the last sentence of the Abstract Methods and Findings section, please describe the main limitation(s) of the study's methodology.**

**Response:** We thank the editor for this valuable suggestion. We have added the main limitation of the study's methodology in the last sentence of the Abstract Methods and Findings section.

**Comment 14. In the abstract, please include the important dependent variables that are adjusted for in the analyses.**

**Response:** We thank the editor for this guidance. However, this item is not applicable to our study, as no dependent variables were adjusted for in the analyses.

**Comment 15. In the author summary, please revise formatting and ensure you use bullet points.**

**Response:** We thank the editor for the reminder and have revised the formatting of our author summary and ensured that bullet points are used as requested.

**Comment 16. In the author summary, in the final bullet point of 'What Do These Findings Mean?', please include the main limitations of the study in non-technical language.**

**Response:** We thank the editor for the clarification and have included the main limitations of the study in the final bullet point of 'What Do These Findings Mean?'.

**Comment 17. PLOS Medicine requires that the de-identified data underlying the specific results in a published article be made available, without restrictions on access, in a public repository or as Supporting Information at the time of article publication, provided it is legal and ethical to do so. Please see the policy at
http://journals.plos.org/plosmedicine/s/data-availability
and FAQs at
http://journals.plos.org/plosmedicine/s/data-availability#loc-faqs-for-data-policy "**

**Response:** We thank the editor for the guidance on data availability. We confirm that we have reviewed PLOS Medicine's Data Availability policy. The de-identified data underlying the results of our study will be made available without restrictions on access in a public repository or as Supporting Information at the time of publication, as required. Our Data Availability Statement has been included in the manuscript.

**Comment 18. PLOS defines the “minimal data set” to consist of the data set used to reach the conclusions drawn in the manuscript with related metadata and methods, and any additional data required to replicate the reported study findings in their entirety. Authors do not need to submit their entire data set, or the raw data collected during an investigation. Please submit the following data:

The values behind the means, standard deviations and other measures reported;
The values used to build graphs;
The points extracted from images for analysis."**

**Please ensure that journal name abbreviations match those found in the National Center for Biotechnology Information (NCBI) databases (http://www.ncbi.nlm.nih.gov/nlmcatalog/journals), and are appropriately formatted and capitalised.**

**Response:** We thank the editor for the reminder and have updated the relevant raw data to the Research Data Deposit platform ([www.research.org.cn](http://www.research.org.cn)), with approval number: RDDA2026867164. All raw data can be viewed via the platform.

**Comment 19. Please provide the full name(s) of the institutional review board(s) that provided ethical approval.**

**Response:** We thank the editor for the guidance and have provided the full name of the ethical committee (the ethical committee of Sun Yat-sen University Cancer Center).

**Comment 20. Figures cannot be reproduced from other sources that are not CC-BY. Please provide a different figure.**

**Response:** We thank the editor for this reminder. However, this requirement is not applicable to our manuscript, since all figures presented in our manuscript are original and produced by us.

**Comment 21. Please provide titles and legends for all figures and tables (including those in Supporting Information files). Please define all acronyms used in each figure or table in its corresponding legend.**

**Response:** We thank the editor for the helpful reminder and we have checked all figures and tables to confirm that all titles and legends have been provided, and all acronyms used in figures or tables have been defined in corresponding legends.

**Comment 22. Please ensure that where relevant figures include 95% CIs.**

**Response:** We thank the editor for this reminder. We have ensured that relevant figures include 95% Cis, and we have provided supplementary explanations in the corresponding legends.

**Comment 23. Please show graph axes beginning at zero. If this is not possible, please show a break in the axis.**

**Response:** We thank the editor for the careful reminder. However, this requirement is not applicable to some of our figures, including the two-way sensitivity analysis plots, the scatter plot of probabilistic sensitivity analysis, and the tornado diagram from the one-way sensitivity analysis.

**Comment 24. When a p value is given, please specify the statistical test used to determine it in the legend.**

**Response:** We thank the editor for this reminder. We have specified the statistical test used to calculate the P values in the corresponding legend.

**Comment 25. Please provide the unadjusted comparisons as well as the adjusted comparisons in all relevant Tables.**

**Response:** We thank the editor for this suggestion. However, this requirement is not applicable to our manuscript and tables.

**Comment 26. Please specify the variables controlled for in all relevant Tables.**

**Response:** We thank the editor for this suggestion. However, this requirement is not applicable to our manuscript and tables.

**Comment 27. Please consider if moving some of the figures shared in the supplementary information into the main text would aid the reader.**

**Response:** We thank the editor for this careful suggestion. We have carefully considered and determined that no more figures need to be moved from the supplementary information to the main text to aid reader understanding.

**Comment 28. Please include an Acknowledgments section in your manuscript.**

**Response:** We thank the editor for this reminder, and we have added an Acknowledgments section in our manuscript.

**Comment 29. Please include an acknowledgment of study participants.**

**Response:** We thank the editor for this reminder, and we have added an acknowledgment of study participants in our manuscript.

**Comment 30. Please ensure that the study is reported according to the CHEERS guideline (available from: https://www.equatornetwork.org/reporting-guidelines/cheers) and include the completed checklist as Supporting Information. Please add the following statement, or similar, to the Methods: "This study is reported as per the
Strengthening the Consolidated Health Economic Evaluation Reporting Standards 2022 (CHEERS 2022) Statement (S1 Checklist)." When completing the checklist, please use section and paragraph numbers, rather than page numbers.**

**Response:** We thank the editor for the reminder. We have added the required statement to the Methods section and included the completed CHEERS checklist in our Supplementary Information (**please see the Supplementary Table S2**). Moreover, we have used section and paragraph numbers in the checklist, as requested.

**REFERENCE**

1.	Dang J, Li G, Ma L, Diao R, Zang S, Han C, et al. Predictors of grade≥ 2 and grade≥ 3 radiation pneumonitis in patients with locally advanced non-small cell lung cancer treated with three-dimensional conformal radiotherapy. Acta Oncologica. 2013;52(6):1175-80.

2.	Lu Y, Dai Z, Chang F, Wang L, He J, Shi P, et al. Whether and how disutilities of adverse events were used in the economic evaluation of drug therapy for cancer treatment. Pharmacoeconomics. 2023;41(3):295-306.

3.	Zhao M, Shao T, Yin Y, Fang H, Shao H, Tang W. Adverse event costs and cost-effectiveness analyses of anticancer drugs: a systematic review. JAMA network open. 2025;8(5):e2512455.

4.	Ara R, Wailoo A. Using health state utility values in models exploring the cost-effectiveness of health technologies. Value in Health. 2012;15(6):971-4.

5.	Wolowacz SE, Briggs A, Belozeroff V, Clarke P, Doward L, Goeree R, et al. Estimating health-state utility for economic models in clinical studies: an ISPOR good research practices task force report. Value in Health. 2016;19(6):704-19.

6.	Hou Z, Dong B, Yao Q, Chen H, Shao Q, Li M, et al. Pirfenidone for grade 2 and grade 3 radiation-induced lung injury: a multicentre, open-label, randomised, phase 2 trial. The Lancet Oncology. 2025;26(12):1552-62.

7.	Shi G, Park SH, Ren H, Xue M, Lu X, Dong P, et al. Cost analysis for different sequential treatment regimens for metastatic renal cell carcinoma in China. Journal of Medical Economics. 2018;21(12):1150-8. doi: 10.1080/13696998.2018.1515769.

8.	Chen J, Hu G, Chen Z, Wan X, Tan C, Zeng X, et al. Cost-effectiveness Analysis of Pembrolizumab Plus Axitinib Versus Sunitinib in First-line Advanced Renal Cell Carcinoma in China. Clin Drug Investig. 2019;39(10):931-8. Epub 2019/06/30. doi: 10.1007/s40261-019-00820-6. PubMed PMID: 31250401.

9.	Neumann PJ, Cohen JT, Weinstein MC. Updating cost-effectiveness—the curious resilience of the $50,000-per-QALY threshold. N Engl J Med. 2014;371(9):796-7.

10.	Wu C-F, Lin L, Mao Y-P, Deng B, Lv J-W, Zheng W-H, et al. Liquid biopsy posttreatment surveillance in endemic nasopharyngeal carcinoma: a cost-effective strategy to integrate circulating cell-free Epstein-Barr virus DNA. BMC medicine. 2021;19(1):193.

11.	Han Z, Xi Y, Hu J, Wang Y, Ni H. Cost-effectiveness analysis of eight first-line treatments for metastatic hormone-sensitive prostate cancer in China. Frontiers in Pharmacology. 2025;16:1684966.

12.	Hutubessy R, Chisholm D, Edejer TT-T, Who-choice. Generalized cost-effectiveness analysis for national-level priority-setting in the health sector. Cost effectiveness and resource allocation. 2003;1(1):8.

13.	Bertram MY, Lauer JA, De Joncheere K, Edejer T, Hutubessy R, Kieny M-P, et al. Cost–effectiveness thresholds: pros and cons. Bulletin of the World Health Organization. 2016;94(12):925.

14.	Liu G, Hu S, Wu J, Wu J, Dong Z, Li H. China guidelines for pharmacoeconomic evaluations. China market. 2020.

15.	Zhao M, Chi Z, Pan X, Yin Y, Tang W. Economic evaluation of nemonoxacin, moxifloxacin and levofloxacin in the treatment of early community-acquired pneumonia with possible pulmonary tuberculosis. International Journal of Environmental Research and Public Health. 2022;19(8):4816.

16.	Ma K, Lu Y, Jiang S, Tang J, Li X, Zhang Y. The relative risk and incidence of immune checkpoint inhibitors related pneumonitis in patients with advanced cancer: a meta-analysis. Frontiers in pharmacology. 2018;9:1430.

17.	Antonia SJ, Villegas A, Daniel D, Vicente D, Murakami S, Hui R, et al. Durvalumab after chemoradiotherapy in stage III non–small-cell lung cancer. New England Journal of Medicine. 2017;377(20):1919-29.

18.	Brahmer JR, Lacchetti C, Schneider BJ, Atkins MB, Brassil KJ, Caterino JM, et al. Management of immune-related adverse events in patients treated with immune checkpoint inhibitor therapy: American Society of Clinical Oncology Clinical Practice Guideline. Journal of Clinical Oncology. 2018;36(17):1714-68.

19.	Haanen J, Carbonnel F, Robert C, Kerr KM, Peters S, Larkin J, et al. Management of toxicities from immunotherapy: ESMO Clinical Practice Guidelines for diagnosis, treatment and follow-up. Annals of oncology. 2017;28:iv119-iv42.
