# Supplementary material for: Cost-effectiveness of pirfenidone for patients with cancer and grade 2 or grade 3 radiation-induced lung injury: A modeling study
Source: PLoS Med. 2026 Aug 5;23(8):e1004817. doi: 10.1371/journal.pmed.1004817 (PMC13440887; doi:10.1371/journal.pmed.1004817)
Supplement: Supplementary file 5 [file pmed.1004817.s005.docx]

Dear professor Tosun,

Thank you for the opportunity to revise our manuscript “***Cost-Effectiveness Analysis of Pirfenidone for Cancer Patients with Grade 2 and Grade 3 Radiation-Induced Lung Injury* (PMEDICINE-D-25-03853R1)**”. We sincerely thank the reviewers for their time and insightful comments, which have significantly strengthened this work. Our point-by-point responses are provided below. Text has been added/modified, and all changes are indicated in the manuscript, with corresponding locations provided.

We thank the editorial office for the insightful comments and overall positive feedback on our work. We sincerely hope the revised manuscript, which is strengthened by the additional analyses, is now suitable for publication in ***PLOS Medicine*** and look forward to hearing from you.

Yours Sincerely,

Ming Chen, M.D.

Department of Radiation Oncology, Sun Yat-sen University Cancer Center, State Key Laboratory of Oncology in South China, Collaborative Innovation Center of Cancer Medicine. No.651, Dongfeng East Road, Guangzhou, Guangdong, 510060, P.R. China

Tel: 86-20-87340540

E-mail: [chenming@sysucc.org.cn](mailto:chenming@sysucc.org.cn)

**POINT-TO-POINT RESPONSE**

**Reviewer #1**

**This is an interesting Cost-Effectiveness Analysis on Pirfenidone for Cancer Patients with Grade 2 and Grade 3 Radiation-Induced Lung Injury. However, there are some major issues needing attention.**

**Comment 1. There is no clear justification why Markov simulations were used for analyses in the first place. Why not direct costing analysis? It's well known that Markov simulation Analysis is a dynamic model that simulates transitions between discrete health states over many cycles, often representing lifetime horizons. However, in this study the follow up time is only 24 weeks, not that long. Plus, there are issues with Markov model approach such as very complex, data-intensive, lower transparency and difficult to follow.**

**Response:** We sincerely thank the reviewer for raising this important methodological concern regarding the use of a Markov model instead of a direct costing analysis. We have clarified the rationale in the ***Methods section*** of the revised manuscript ***(please see the Methods section, lines 138-144 on page 8)***. First, Markov models enable the dynamic integration of patient distributions across multiple health states during follow-up, together with associated clinical events, costs, and utility changes[1]. In our study, this model captures transitions among states such as no RP, RP plus severe acute pulmonary exacerbation (APE), and death, while incorporating event-driven costs like hospitalization for severe APE. This structured framework allows estimation of total costs and health outcomes while tracking their accumulation over time and quantifying the contribution of different disease states to overall cost-effectiveness[2]. In contrast, direct costing analysis provides only static estimates of total costs and cannot account for how transitions between health states influence economic results[3]. Second, Markov models allow repeated simulation of potential disease progression pathways using techniques such as Monte Carlo simulation[4, 5]. This approach reflects the stochastic and heterogeneous nature of clinical events observed in practice[6]. Consequently, the results represent expected outcomes across multiple potential trajectories rather than a single observed pathway. Direct costing analyses are limited to the single observed pathway and may not adequately capture variability in disease progression or healthcare settings, thus limiting the generalizability of the findings. For example, a single severe APE event can substantially affect total costs, but it occurs randomly. Repeated Markov simulations smooth such stochastic fluctuations, yielding more stable estimates of cost-effectiveness. Third, Markov models support comprehensive sensitivity analyses, allowing quantification of parameter uncertainty and its impact on cost-effectiveness conclusions[7, 8]. By adjusting key parameters, the model identifies major drivers of economic outcomes and can be readily extended to alternative follow-up horizons or healthcare contexts. For instance, cost parameters can be adjusted to evaluate the impact of pirfenidone price fluctuations under different insurance or procurement policies, thereby improving the robustness and external applicability of our analysis. Direct costing analysis, in contrast, cannot adequately quantify parameter uncertainty or explore alternative scenarios, resulting in conclusions restricted to a specific cohort.

Regarding the 24-week time horizon, this choice was made for several reasons. First, our clinical trial primarily focused on the acute inflammatory phase of RILI. During the treatment period, both pirfenidone and glucocorticoids mainly exert anti-inflammatory effects; therefore, the analysis intentionally focused on the acute inflammatory phase of RILI, which typically occurs within several weeks to approximately six months after radiotherapy[9, 10]. A 24-week horizon adequately captures this clinically relevant period. Second, the model structure, key parameters, and follow-up duration closely align with those reported in our phase II RCT, whose follow-up horizon is 24 weeks as well. This consistency helps ensure the reliability of model inputs, and strengthens the internal validity of the model outcomes. Third, previous studies have demonstrated the feasibility of applying Markov models to short- and medium-term disease processes, with time horizons ranging from one month to six months[11, 12]. Particularly, this method can capture key disease phases while avoiding the dilution of short-term intervention effects associated with longer time horizons[11]. In our study, this approach helps minimize the potential confounding influence of subsequent antifibrotic effects on the treatment differences between the two groups, thus allowing a more accurate assessment of the true effectiveness of the pirfenidone regimen during the early inflammatory phase. We thank the reviewer for raising this important point, which helped us clarify the rationale for the selected time horizon. We have supplemented the rationale for this approach in the ***Methods section*** of the revised manuscript ***(please see the Methods section, lines 148-154 on page 8)***.

Finally, we acknowledge that Markov models are inherently more complex and data-intensive than direct costing approaches. To ensure transparency and interpretability, we undertook several measures. First, the model was developed following the CHEERS 2022 reporting guidelines to ensure methodological clarity ***(please see the Supplementary Table S2)***. Second, detailed descriptions of the model structure, parameter derivation, and assumptions are provided in ***the Methods and Supplementary Materials*** ***(please see the Methods section and Supplementary Materials).*** Third, we also included a visual representation of the model structure, a comprehensive table summarizing all parameters, and multiple sensitivity analyses. Key parameters such as drug costs and APEFS rates were further explored through threshold analyses. Fourth, model outcomes were visualized using tornado plots, scatter plots, cost-effectiveness acceptability curves, and contour plots to facilitate interpretation. Fifth, the published design and results of the corresponding RCT provide transparent and verifiable sources for the model structure and parameters.

We have clarified the revised manuscript to better explain the rationale for selecting the Markov modeling approach. We sincerely thank the reviewer for raising this important comment, which has helped us improve the clarity and rigor of our methods.

**Comment 2. Could authors consider combining both direct costing and Markov simulation in the study, so that one can get more credible cost estimates, improved transparency, making the Markov model more realistic, addressing both short-term and long-term perspectives in one paper.**

**Response:** We sincerely thank the reviewer for the valuable suggestion and fully agree that combining direct costing analysis with the Markov model can enhance the credibility of the results. In line with this suggestion, we have added a direct costing analysis based on individual patient-level data from our RCT, providing real-world cost estimates to complement the Markov model and have incorporated the direct costing analysis into the ***Discussion section*** ***(please see the Discussion section, lines 455-476 on page 23-24).***

Healthcare resource utilization during follow-up included investigational drug use, follow-up examinations, laboratory tests, hospitalizations, and outpatient visits[13, 14]. Total costs per patient were calculated by multiplying resource use by corresponding unit prices and summing across categories. All costs were converted to U.S. dollars using the exchange rate on August 5, 2025 (1 USD = 7.2011 CNY). Investigational drug costs were calculated based on individualized dosing and treatment duration, accounting for real-world treatment modifications such as dose adjustments, discontinuation, and occasional self-escalation. Based on individual-level treatment records, the average investigational drug cost was $2,550 per patient in the pirfenidone group and $1.97 per patient in the glucocorticoid group.

According to the study protocol, participants completed five scheduled follow-up visits, each including pulmonary function testing and chest CT, together with routine laboratory assessments (complete blood count, blood biochemistry, and urinalysis). Patients who developed varying degrees of radiation-induced lung injury (RILI) underwent additional examinations according to clinical guidelines for radiation pneumonitis[15, 16], including chest X-ray, non-contrast CT, pulmonary function testing, and bronchoscopy and corresponding laboratory tests (complete blood count, blood biochemistry, inflammatory markers, arterial blood gas analysis, sputum culture, and blood culture). Unit costs were derived from the official pricing standards of Medical Insurance Administration Bureau of Guangzhou and were consistent with those listed in ***Supplementary Table S1 (please see the Supplementary Table S1).*** Detailed follow-up schedules and examination protocols are described in the ***Supplementary Methods (please see the Supplementary Methods).*** To maintain methodological consistency, empirically increased follow-up frequencies adopted by a small number of centers were not considered in this costing analysis, and standard follow-up schedules were applied. After accounting for an approximate 23.3% loss to follow-up, the average follow-up and laboratory testing costs were $1,458.81 per patient in the pirfenidone group and $1,995.02 per patient in the glucocorticoid group.

Finally, healthcare utilization related to clinical events was assessed using patients’ medical records, including hospitalizations for severe APE or serious treatment-related adverse events, ICU admissions, and outpatient visits for mild APE or mild adverse events. Because only the occurrence and treatment of severe APE or treatment-related adverse events were recorded across participating centers and historical cost data were unavailable for few sites, hospitalization costs were calculated using the average of available records. The mean hospitalization cost for severe APE or severe adverse events was $3,421. During the study period, two patients required ICU admission, with an average cost of $35,672 per case. The average outpatient visit cost, calculated based on recorded medications and treatment duration with drug prices obtained from the Medical Insurance Administration Bureau of Guangzhou, was $26. Based on the observed utilization rates, the average hospitalization- and outpatient-related costs were $855.60 per patient in the pirfenidone group and $1,882.52 per patient in the glucocorticoid group.

After aggregating all cost components, the mean total cost per patient over the 24-week follow-up period was estimated at $4,864 in the pirfenidone plus glucocorticoids group and $3,880 in the standard glucocorticoid group.

We fully agree with the reviewer that combining both direct costing and Markov simulation in the study can improve the credibility and transparency of economic evaluations. Therefore, we further compared the observed 24-week per-patient costs with the costs predicted by the Markov model to assess its internal validity. Using the Markov model-estimated costs as the benchmark, the model-estimated costs showed deviations of 3.3% ($4,864 vs. $5,029) in the pirfenidone plus glucocorticoids group and 12.6% ($3,880 vs. $4,438) in the standard glucocorticoid group, indicating good agreement between the model estimates and the direct costing data. It should be noted that unlike our model, which incorporates complete indirect cost parameters, our RCT did not capture specific indirect costs, such as transportation, lodging, or productivity losses, which partially explains why the model-predicted costs were slightly higher than the observed costs[17, 18]. In particular, the higher incidence of APE increased related indirect costs in the standard glucocorticoid group resulted in larger deviations compared with the pirfenidone group. This explainable difference further supports the rationality of the Markov model structure and the validity of the data, providing more comprehensive evidence for the cost-effectiveness analysis.

In response to this valuable suggestion, we have incorporated a direct costing analysis based on individual patient-level data from our RCT to complement the Markov model. The contents have been added to the ***Discussion section (please see the Discussion section, lines 455-476 on page 23-24).***

**Reviewer #2**

**This is a well-designed and clinically significant cost-effectiveness analysis. The study evaluates the economic value of adding pirfenidone to standard glucocorticoids for grade 2-3 radiation-induced lung injury, based on the authors' own Phase II RCT data. The results demonstrate a significant cost-effectiveness advantage for the combination strategy. The overall quality of the manuscript is high, and it provides valuable insights for clinical practice and healthcare policy. I have the following comments:**

**Comment 1. According to the official methodological guidelines of the International Society for Pharmacoeconomics and Outcomes Research (ISPOR) regarding the definition of a "societal perspective," the manuscript currently only mentions transportation expenses and wage loss due to work absenteeism. However, other relevant societal costs, such as caregiver services, special diets, and other indirect costs, are not addressed. Further clarification on the comprehensiveness of the societal cost calculation is needed.**

**Response:** We sincerely thank the reviewer for the valuable comment regarding the comprehensiveness of the societal cost assessment. In the original manuscript, we included transportation expenses and wage loss due to work absenteeism as societal costs[19, 20]. Additional societal costs during follow-up, such as extra dietary expenses, were not incorporated in the preliminary analysis.

Considering that the guidelines of the International Society for Pharmacoeconomics and Outcomes Research (ISPOR) recommend that cost-effectiveness analyses conducted from a societal perspective should include all relevant costs associated with societal resource consumption[21, 22], we further supplemented parameters related to non-medical costs and indirect costs in addition to direct medical costs. Specifically, in the revised manuscript, we incorporated patients’ meal, accommodation, and transportation expenses during follow-up into the model. For hospitalized patients, productivity loss and supplementary nutrition costs were also included. Furthermore, to enhance the clarity and transparency of the cost parameters, we explicitly specified hospitalization-related costs, including bed charges and nursing care expenses ***(please see the Table of additional cost parameters below).*** All relevant parameter adjustments have been updated and supplemented in ***Supplementary Table S1***, and corresponding revisions have also been made in the ***Methods section*** of the revised manuscript ***(please see the Supplementary Table S1; please see the Methods section, line 218-225 on page 11-12).***

Due to the parameter refinements, the ICER of pirfenidone for patients with grade 2 or grade 3 RILI decreased slightly from $21,129 per QALY to $20,956 per QALY. The updated baseline results are presented in revised ***Table 1 (please see Table 1).*** The inclusion of non-medical costs does not alter the cost-effectiveness of the pirfenidone strategy. Instead, with the addition of these non-medical costs, the economic advantage of the pirfenidone strategy becomes even more pronounced.

We sincerely thank the reviewer for this constructive suggestion.

Table of additional cost parameters

| **Parameter** | **Pirfenidone plus glucocorticoids** | | **Glucocorticoids alone** | | **Distribution** | **Reference** |
| --- | --- | --- | --- | --- | --- | --- |
|  | **Base-Case Estimate** | **Range** | **Base-Case Estimate** | **Range** |  |  |
| Level I nursing care costs | 264.4 | 212-317 | 264.4 | 212-317 | Gamma | MIAB |
| Level II nursing care costs | 140.0 | 112-168 | 140.0 | 112-168 | Gamma | MIAB |
| Bed charges | 194.4 | 156-233 | 194.4 | 156-233 | Gamma | MIAB |
| Direct non-health care cost^†^ | 37.4 | 30-45 | 37.4 | 30-45 | Gamma | [23, 24] |
| Additional nutrition for inpatients | 28.4 | 23-34 | 28.4 | 23-34 | Gamma | [24] |
| Indirect cost^‡^ | 370.8 | 297-445 | 370.8 | 297-445 | Gamma | [25] |

Abbreviations: MIAB, Medical Insurance Administration Bureau of Guangzhou

^†^ Includes the costs of transportation, accommodation, and additional meal during follow-up.

^‡^ Refers to wage loss related to patients' and caregivers' time off work.

**Comment 2. The price of pirfenidone is based on the 2025 Guangzhou medical insurance negotiation price, yet the conclusion suggests "global applicability." To strengthen the persuasiveness of this argument, I recommend supplementing the analysis with international reference prices or explicitly discussing this as a limitation regarding the generalizability of the findings.**

**Response:** We sincerely thank the reviewer for this insightful comment. We fully agree that variations in drug pricing across countries and regions may influence the generalizability of the study findings. To ensure consistency with our RCT, the unit price of pirfenidone in the base-case analysis of this study was derived from the 2025 Guangzhou medical insurance negotiated price. To further strengthen the robustness of our findings, we collected international reference prices for pirfenidone from the online pharmacy platform[26], covering multiple healthcare settings including the United States, Canada, the United Kingdom, and India. The summarized pricing data are provided in the ***Supplementary*** ***Table S5 (please see the Table S5 below)***, showing that the cost per 200-mg tablet generally fluctuates below $3.00.

In the one-way sensitivity analysis in our study, the pirfenidone strategy remained cost-effective as long as the per-cycle cost of pirfenidone did not exceed $992.7. Translating this into tablet-unit pricing, pirfenidone would be cost-effective with its price lower than $5.1 per 200-mg tablet. Therefore, under the healthcare contexts of multiple countries, pirfenidone is likely to remain cost-effective, which further strengthens the robustness of our conclusions. These additional analyses have now been incorporated into the ***Discussion section*** of the revised manuscript, with the pharmacy platforms cited as references ***(please see the Discussion section, line 444-454 on page 22-23).***

Nevertheless, we strongly agree with the reviewer that differences in national pricing systems, healthcare structures, and reimbursement policies may still limit the external applicability of the results. Therefore, we have revised the Discussion section to adopt a more cautious interpretation, emphasizing that pirfenidone may have the potential for broader clinical applicability across different healthcare settings, without implying universal global applicability ***(please see the Discussion section, line 438-444 on page 22).***

**Table S5．**Summary of pirfenidone unit prices across different countries

| Country | Price ($ / tablet) |
| --- | --- |
| Australia | 0.38-3.00 |
| Canada | 0.38-3.00 |
| Mauritius | 0.38-3.00 |
| New Zealand | 0.38-3.00 |
| United Kingdom | 0.38-3.00 |
| India | 0.38-1.57 |
| United States | 0.38-1.40 |

Note: All prices refer to pirfenidone tablets with a strength of 200 mg per tablet.

**Comment 3. The baseline health utility value of 0.712 is sourced from a 2008 study involving a predominantly White, UK-based Western population with advanced NSCLC. Applying this value directly to the current study set in China raises questions about potential differences due to ethnicity or cross-cultural health preferences. The authors should address and justify this choice.**

**Response:** We sincerely thank the reviewer for raising this important and thoughtful concern. The baseline health utility value of 0.712 was selected based on the following considerations. First, non-small cell lung cancer (NSCLC) represented the most prevalent subgroup (56.9%) among the patients enrolled in our RCT ***(please see the Supplementary Materials: Table S4).*** Moreover, the eligibility criteria of our RCT enrolled patients with no tumor progression after treatment. Therefore, the health utility value of NSCLC patients with no disease progression was considered as the baseline health state in our model. However, large-scale studies reporting health utility values for this population in Chinese patients are currently limited. Consequently, we adopted a health utility value of 0.712 derived from an observation study in which most participants were White (69.3%).

We fully agree with the reviewer that applying estimates derived from a predominantly White population to a Chinese population may introduce uncertainty, particularly due to potential ethnic differences and cross-cultural variations in health preferences. To address this concern, we performed comprehensive sensitivity analyses to further assess the robustness of our findings. Specifically, our one-way and probability sensitivity analyses were both based on a relatively wide range for the baseline health utility value (0.600-0.981).This range was determined based on the assumption that the health utility value for patients without tumor progression should be lower than that of the general population but higher than that of patients with progressive disease[27]. Accordingly, the upper bound (0.981) was obtained from studies reporting health utility values for the general population in China[28, 29], representing the highest plausible health utility level in our analysis. Under this assumption, the ICER was $13,941.541 per QALY. The lower bound (0.600) was derived from the studies reporting utility values for small cell lung cancer (SCLC) patients with progressive disease[30]. This value was selected because it is lower than the reported utility values for patients with progressive NSCLC, esophageal cancer, and breast cancer[31, 32], thereby allowing the analysis to cover a broader and more convincing range of potential variation. Under this scenario, the corresponding ICER was $26,509.995 per QALY. Based on these analyses, potential variations in the baseline health utility value are unlikely to substantially affect the overall cost-effectiveness conclusions of our model.

Following the reviewer’s suggestion, we have clarified the source, rationale, and applicability of the health utility values in the ***Clinical Scenario Analysis*** section of the revised manuscript ***(please see the Clinical Scenario Analysis section, line 352-374 on page 18-19).***

**Comment 4. The model's time horizon is set at 24 weeks, covering only the acute inflammatory phase of RILI. However, the core disabling and life-threatening risks of RILI stem from its chronic, progressive fibrotic phase, which can last for years. As an anti-fibrotic agent, pirfenidone's primary clinical and economic value likely lies in modifying this long-term process. Although the authors have mentioned this as a limitation, even in the absence of long-term RCT data, constructing a simple extrapolation model that includes states for long-term fibrosis progression and conducting scenario analyses would be valuable. It is recommended to add a paragraph in the "Discussion" section elaborating on how future analyses could be improved to address this.**

**Response:** We sincerely thank the reviewer for this constructive comment. The 24-week time horizon adopted in our base-case model was selected for several reasons. First, the model structure, key parameters, and follow-up duration were closely aligned with those reported in our phase II randomized controlled trial (RCT). This alignment helps ensure the reliability of model inputs and enhances the internal validity and credibility of the model outcomes. Second, during the treatment period, both pirfenidone and glucocorticoids primarily exerted anti-inflammatory effects. Therefore, the analysis was intentionally focused on the acute inflammatory stage of RILI. Third, the long-term anti-fibrotic effects of pirfenidone in the context of RILI remain to be further confirmed in future phase III RCTs (NCT07388680). Currently, long-term follow-up data were still limited to support the development of a fully evidence-based long-term model.

Nevertheless, we fully agree with the reviewer that the 24-week time horizon used in our model primarily captures the acute inflammatory phase of RILI and therefore cannot fully reflect the long-term clinical consequences associated with the chronic and progressive fibrotic phase, which may persist for years. In this context, exploring the potential long-term value of pirfenidone as an anti-fibrotic agent is indeed highly important.

To explore the potential anti-fibrotic benefits of pirfenidone, we perform an extrapolation analysis to evaluate its potential long-term economic impact. All transition probability parameters in the extrapolated model were derived from the real-world follow-up data collected from our study cohort, whereas health utility values and relevant treatment costs were obtained from the published literature. Based on the follow-up schedule, the cycle length of the Markov model was set at 4 weeks, and an extended model with a 15-year time horizon[33] ***(please see the Table of parameters for extrapolation model below).*** Specifically, transition probabilities were estimated by calculating the proportion of transitions between pulmonary fibrosis grades observed across two consecutive follow-up visits. Health utility values were estimated by first calculating the mean forced vital capacity percentage (FVC%) for patients within each fibrosis grade and subsequently mapping these values to corresponding utility estimates according to relevant literature sources[34, 35].

The results suggested that, compared with glucocorticoids alone, pirfenidone plus glucocorticoids could generate an additional 2.348 QALYs at an incremental cost of $52,724, resulting in an incremental cost-effectiveness ratio (ICER) of $22,453 per QALY gained. This ICER remained well below the commonly accepted willingness-to-pay (WTP) threshold of $100,000 per QALY, suggesting that pirfenidone may still demonstrate favorable cost-effectiveness when its potential long-term benefits are taken into consideration ***(please see the Table of cost-effectiveness analysis of extrapolation model below).***

Notably, although this extrapolated model was constructed based on the real-world follow-up data obtained in our RCT, several important limitations should be acknowledged. First, our Markov cycle was limited to 4 weeks due to the follow-up interval, and the total follow-up duration was around half-a-year, which restricts our model to fully capture the true long-term anti-fibrotic effects of pirfenidone and should be interpreted primarily as illustrating potential trends rather than providing definitive long-term estimates. Second, the cohort size was relatively small with the total sample size fewer than 100. Third, the observed health stated only included no fibrosis, grade 1 fibrosis, grade 2 fibrosis, death due to pulmonary fibrosis, and the occurrence of mild or moderate APE. Since no cases of grade 3 or grade 4 fibrosis were observed during follow-up and no relevant data from published literature, parameters corresponding to grade 3 and grade 4 fibrosis were unavailable in the model. Consequently, the credibility of extrapolated analyses might be limited due to this absence of data for grade 3 or grade 4 fibrosis.

Considering the limitations in follow-up data and cohort size, we do not present the results of this extrapolation model in the Results section. Instead, we incorporated a discussion of these exploratory analyses in the ***Discussion section*** of our revised manuscript ***(please see the Discussion section, line 529-549 on page 26-27).*** We believe that this approach better ensures the rigor of this study.

**Table of parameters for extrapolation model**

| **Parameter** | **Pirfenidone plus glucocorticoids** | | **Glucocorticoids alone** | | **Reference** |
| --- | --- | --- | --- | --- | --- |
|  | **Base-Case Estimate** | | **Base-Case Estimate** | |  |
| **Transition probabilities (per cycle)** | | | | | |
| Probability of transition from no fibrosis to each health state below | | | | | |
| no fibrosis | 0.75 | | 0.60 | | Trial cohort |
| grade 1 fibrosis | 0.25 | | 0.40 | | Trial cohort |
| grade 2 fibrosis | 0.00 | | 0.00 | | Trial cohort |
| grade 3 fibrosis | 0.00 | | 0.00 | | Trial cohort |
| grade 4 fibrosis | 0.00 | | 0.00 | | Trial cohort |
| grade 5 fibrosis | 0.00 | | 0.00 | | Trial cohort |
| Probability of transition from grade 1 fibrosis to each health state below | | | | | |
| no fibrosis | 0.07 | | 0.02 | | Trial cohort |
| grade 1 fibrosis | 0.92 | | 0.96 | | Trial cohort |
| grade 2 fibrosis | 0.01 | | 0.01 | | Trial cohort |
| grade 3 fibrosis | 0.00 | | 0.00 | | Trial cohort |
| grade 4 fibrosis | 0.00 | | 0.00 | | Trial cohort |
| grade 5 fibrosis | 0.00 | | 0.02 | | Trial cohort |
| Probability of transition from grade 2 fibrosis to each health state below | | | | | |
| no fibrosis | 0.00 | | 0.00 | | Trial cohort |
| grade 1 fibrosis | 0.78 | | 0.25 | | Trial cohort |
| grade 2 fibrosis | 0.22 | | 0.75 | | Trial cohort |
| grade 3 fibrosis | 0.00 | | 0.00 | | Trial cohort |
| grade 4 fibrosis | 0.00 | | 0.00 | | Trial cohort |
| grade 5 fibrosis | 0.00 | | 0.00 | | Trial cohort |
| Probability of APE occurrence in each health state | | | | | |
| no fibrosis | 0.00 | | 0.10 | | Trial cohort |
| grade 1 fibrosis | 0.06  0.100-0.150 | | 0.17 | | Trial cohort |
| grade 2 fibrosis | 0.22 | | 0.13 | | Trial cohort |
| Probability of mild APE occurrence in each health state | | | | | |
| no fibrosis | 1.00 | | 1.00 | | Trial cohort |
| grade 1 fibrosis | 0.78 | | 0.61 | | Trial cohort |
| grade 2 fibrosis | 0.50 | | 0.70 | | Trial cohort |
| **Costs ($ per cycle)** | | | | | |
| Oral glucocorticoids (first 6 cycles) | 0.3 | | 0.3 | | MIAB |
| Pirfenidone (first 6 cycles) | 606.0 | | — | | MIAB |
| Patient monitoring | 18.7 | | 18.7 | | [35] |
| Oxygen supplementation | 300.2 | | 300.2 | | [35] |
| Palliative care | 1464.0 | | 1464.0 | | [35] |
| **Utility** |  |  |  |  |  |
| no fibrosis | 0.712 | | 0.712 | | [36] |
| grade 1 fibrosis | 0.685 | | 0.685 | | [34, 36, 37] |
| grade 2 fibrosis | 0.654 | | 0.654 | | [34, 36, 37] |
| grade 3 fibrosis | 0.640 | | 0.640 | | [34, 36, 37] |
| grade 4 fibrosis | 0.613 | | 0.613 | | [34, 36, 37] |
| Mild APE decrement | 0.048 | | 0.048 | | [36] |
| Severe APE decrement | 0.200 | | 0.200 | | [38] |

**Table of cost-effectiveness analysis of extrapolation model**

| **Treatment strategy** | **Total values** | | **Incremental values** | | **ICER ($/QALY)** | **NHB (QALY)** |
| --- | --- | --- | --- | --- | --- | --- |
|  | **Cost ($)** | **Effectiveness (QALY)** | **Cost ($)** | **Effectiveness (QALY)** |  |  |
| Glucocorticoids | 27,741 | 3.470 | —— | —— | —— | 3.193 |
| Pirfenidone plus glucocorticoids | 80,465 | 5.818 | 52,724 | 2.348 | 22,453 | 5.013 |

Abbreviations: ICER, incremental cost-effectiveness ratio; QALY, quality-adjusted life-year; NHB, net health benefit

**Comment 5. Nintedanib, like pirfenidone, is an anti-fibrotic drug approved for idiopathic pulmonary fibrosis and progressive fibrosing interstitial lung diseases. While the conclusion that "pirfenidone plus glucocorticoids is cost-effective" is valid within the framework of this study, the assertion that it "should become the standard treatment" is too strong and insufficiently supported by the evidence. It is recommended to add a discussion of this limitation in the "Discussion" section and modify the wording in the conclusion accordingly.**

**Response:** We sincerely thank the reviewer for this valuable comment. We agree with the reviewer that, although our analysis suggests that pirfenidone plus glucocorticoids is cost-effective within the framework of the present study, stating that it “should become the standard treatment” may be too strong and may overstate the level of evidence currently available. As the reviewer appropriately pointed out, other anti-fibrotic agents, such as nintedanib, are also approved for idiopathic pulmonary fibrosis (IPF) and progressive fibrosing interstitial lung diseases, and previous economic evaluations in IPF have suggested that the relative cost-effectiveness of pirfenidone versus nintedanib remains uncertain, with different studies reporting varying results [33, 35]. Therefore, presenting pirfenidone plus glucocorticoids as a definitive standard treatment could potentially lead to misinterpretation of our findings.

In response to the reviewer’s suggestion, we have carefully revised the ***Conclusion*** ***section*** to make it more cautious and appropriate ***(please see the Conclusion section, line 554-557 on page 28).*** Specifically, instead of stating that pirfenidone plus glucocorticoids should become the standard treatment, we now describe it as a promising therapeutic option for RILI, which may provide useful inform for future clinical practice and healthcare decision-making. In addition, we have incorporated a brief discussion in **the *Discussion section*** to acknowledge this limitation and to emphasize that further research, including comparative studies involving other anti-fibrotic therapies such as nintedanib, will be necessary to better clarify the relative clinical and economic value of different treatment strategies ***(please see the Discussion section, line 525-528 on page 26).***

**Comment 6. While cost-effectiveness analyses typically employ QALYs, clinical decision-making often incorporates other "values," such as a treatment's impact on a patient's ability to return to work, caregiver burden, or the influence of complications. The discussion could briefly acknowledge that the present study did not capture these dimensions, which could be a direction for future research.**

**Response:** We sincerely thank the reviewer for this valuable and insightful comment. We agree that although QALYs are commonly used as the primary outcome measure in cost-effectiveness analyses, clinical decision-making often involves additional value dimensions beyond health-related quality of life[39]. These may include factors such as the impact of treatment on patients’ ability to return to work, caregiver burden, and the potential effects of treatment-related complications on patients’ overall well-being[40, 41].

Following the reviewer’s helpful suggestion, we have added a statement in the ***Discussion section*** of the revised manuscript to acknowledge this limitation. Furthermore, we have noted that future clinical studies and real-world research could incorporate these additional value considerations, which may provide more comprehensive health economic evidence to better inform clinical decision-making ***(please see the Discussion section, line 520-525 on page 26).***

**Comment 7. In Supplementary Materials, the title of Table S3 is positioned below the table. Please adjust the formatting accordingly.**

**Response:** We sincerely thank the reviewer for this helpful suggestion. We have moved the title of ***Supplementary Table S3*** above the table. In addition, due to the inclusion of a newly added table in the Supplementary Materials, this table has now been renumbered as ***Supplementary Table S4 (please see the Supplementary Table S4).***

**Reviewer #3**

**Thank you for the opportunity to review this cost-effectiveness analysis of pirfenidone for radiation-induced lung injury (RILI).
This study used robust methodology including a mechanistic Markov model, univariate sensitivity analysis, multivariate PSA, and a CEAC, all of which strengthen the conclusion of very probable cost-effectiveness of the pirfenidone + glucocorticoids strategy. My suggestions are relatively minor.**

**Comment 1. Net health benefit (NHB) and incremental cost-effectiveness are co-primary outcomes, but the Discussion focuses only on ICER. Please also discuss the NHB outcome and whether the result concurred with the hypothesis of the study. For example, NHB could be contextualized by comparing it to NHB benchmarks for other health interventions.**

**Response:** We sincerely thank the reviewer for this insightful comment. We agree with the reviewer that the NHB findings were not sufficiently elaborated in the Discussion section. Following the reviewer’s helpful suggestion, we have now expanded the ***Discussion section*** to incorporate a more explicit interpretation of the NHB results. When discussing the key parameters identified in the sensitivity analysis, we calculated the corresponding NHB values in addition to the ICER values, further supporting the cost-effectiveness conclusions of our study ***(please see the Discussion section******, line 394-396 on page 20 and line 438-441 on page 22).***

For example, when the acute pulmonary exacerbation-free survival (APEFS) rate in the glucocorticoid group was 71.6%, the corresponding ICER was −$72,155 per QALY, with an incremental NHB of 0.061 QALY. As the rate increased to 100%, the ICER rose to $127,505 per QALY, while the incremental NHB decreased to −0.006 QALY, showing consistent trends between NHB and ICER. Similarly, for pirfenidone, a per-cycle cost of $484.8 yielded an ICER of −$3,831 per QALY and incremental NHB of 0.029 QALY; when the cost increased to $727.2, the ICER rose to $45,744 per QALY, while the incremental NHB decreased to 0.015 QALY. Despite the decline in NHB with higher ICERs, it remained positive across the evaluated range, indicating that the pirfenidone strategy maintained cost-effectiveness across the evaluated range.

Overall, jointly interpreting the NHB and ICER results provides a clearer and more comprehensive interpretation of the economic evaluation, with NHB further corroborating the ICER-based conclusions.

**Comment 2. In the supplementary sensitivity analysis, it appears the investigators separately/independently altered disease progression parameters in the pirfenidone group vs. the glucocorticoids group (Figure S2 panel A vs. B). This approach appears unjustified, given that the trial randomly assigned pirfenidone, and the study conceptualizes pirfenidone + glucocorticoids as an alternative scenario to glucocorticoids alone in the same population. If a disease progression parameter were different from the base case (e.g., a progression rate or utility were overestimated), it would differ by the same amount in both the pirfenidone glucocorticoids and the glucocorticoids groups. These parameters should be changed by the same amount in both groups before calculating the resulting ICER.**

**Response:** We sincerely thank the reviewer for this insightful comment. We would like to clarify that all baseline parameters for both treatment strategies were derived from our RCT data or from clinically reasonable estimates, as detailed in the ***Supplementary Methods.*** In the one-way sensitivity analysis, the ranges for parameter variation were either based on published literature or set as ±20% of the base-case values, and the magnitude of variation was applied consistently across both treatment arms. Complete baseline values and corresponding ranges for all parameters are provided in ***Table S1*** to ensure transparency and reproducibility of the results. In ***Figure S2***, the tornado diagram was presented in separate panels (A for the pirfenidone plus glucocorticoids strategy and B for the standard glucocorticoid strategy) solely to improve visual clarity given the large number of parameters included in the analysis ***(please see the Supplementary Table S1 and* *Figure S2).*** This panel separation does not imply that parameters were varied independently for the two treatment groups. Rather, each parameter was varied individually while recalculating the ICER for the comparison between pirfenidone plus glucocorticoids and glucocorticoids alone. The combined impact of simultaneous variation in multiple parameters was assessed and reported through probabilistic sensitivity analysis. We have also revised **the caption of *Figure S2*** to clearly explain this presentation, in order to avoid potential misunderstanding ***(please see the Supplementary Figure S2).***

In addition, we fully agree with the reviewer that the effect of varying a set of disease progression parameters simultaneously in both treatment arms was not explicitly shown. To address this, we conducted two-way sensitivity analyses for each set of disease progression–related parameters. These analyses further support the conclusions of our cost-effectiveness study, showing that, at a WTP threshold of $100,000 per QALY, pirfenidone remains cost-effective in the vast majority of scenarios. Notably, only under the extreme condition where the APEFS rate in the glucocorticoids group exceeds 99.76% would pirfenidone plus glucocorticoids fail to be cost-effective, regardless of the APEFS rate in the pirfenidone therapy group. This analysis has been added to ***Figure 3 and Supplementary Figure S3*** ***(please see the Figure 3 and Supplementary Figure S3 below)***, and the corresponding descriptions have been incorporated into the ***Methods and Results sections*** of the manuscript ***(please see the Methods sections, line 248-249 on page 13; please see the Results sections, line 316-328 on page 16-17).***

**Fig 3. The two-way sensitivity analyses of the Per-cycle non-response rate** **(left) and the APEFS rate (right) in grade 2 or 3 RILI patients.** The blue area indicates parameter combinations for which pirfenidone plus glucocorticoids is the more cost-effective strategy, whereas the yellow area indicates combinations for which standard glucocorticoid therapy is the more cost-effective strategy.

Abbreviations: RILI, radiation-induced lung injury; APEFS, acute pulmonary exacerbation-free survival.

**Fig S3.** **Two-way sensitivity analysis of disease progression-related parameters.** The blue area indicates parameter combinations for which pirfenidone plus glucocorticoids is the more cost-effective strategy, whereas the yellow area indicates combinations for which standard glucocorticoid therapy is the more cost-effective strategy, at the WTP threshold of $100,000 per QALY.

Abbreviations: RILI, radiation-induced lung injury; APEFS, acute pulmonary exacerbation-free survival; APE, acute pulmonary exacerbation; ICU, intensive care unit.

**Comment 3. Quite a few cost items related to RILI healthcare do not have a source/reference provided. Where were these values obtained? Also, for transparency, please disaggregate cost line items into unit cost and number of units, if possible.**

**Response:** We sincerely thank the reviewer for this thoughtful comment. We fully agree that clearly documenting the sources of cost inputs and transparently presenting cost components are essential for ensuring the credibility and reproducibility of health economic analyses.

Regarding the sources of the cost parameters related to RILI healthcare, we would like to clarify that these values were obtained from the Medical Insurance Administration Bureau of Guangzhou (MIAB). To maintain the conciseness of the cost table, we only labeled MIAB in the row reporting the total costs for each health state, while the individual cost components derived from MIAB were not repeatedly labeled. And we only cited references for those cost items obtained directly from published literature. We acknowledge that this presentation may have reduced the transparency of the data sources. We have now revised the ***Table S1*** to clearly indicate MIAB as the data source for all relevant cost items, ensuring that the origin of each cost parameter is explicitly documented. In addition, to prevent similar situation that might mislead readers, we have clearly indicated the range of each cost sub-item parameter in ***Table S1 (please see the Supplementary Table S1).***

In addition, we fully agree with the reviewer that disaggregating cost items into “unit cost × quantity of use” can further enhance the transparency of the analysis. To balance the readability and conciseness of the cost table while providing clearer information on unit costs, we have now reported the unit costs of all treatment practices obtained from MIAB **in the footnote of Table S1**, expressed on a per-procedure or per-use basis. For treatment practices derived from the literature, the costs are reported per Markov cycle (4 weeks) as presented in the original studies. These revisions aim to make the cost structure more transparent and easier for readers to interpret. It should also be noted that the treatment components and their corresponding utilization frequencies were strictly defined according to the specifications described in the ***Supplementary Methods*** ***(please see the footnote of Supplementary Table S1).***

**Comment 4. For utility values, the references listed do not appear to be the source studies that performed time trade-off or other experiments to estimate utility of RILI grades or of the other conditions used to benchmark RILI symptom (dis)utility. Please revise the reference list to indicate the sources for the assumed utility values.**

**Response:** We sincerely thank the reviewer for highlighting the important issue regarding the sources of the utility values. We fully agree that accurate and transparent reporting of the origin of health utility data is essential for methodological rigor and reproducibility in health economic research.

To address this, we traced back to the original studies and clearly indicated the sources of the utility values in ***Table S1***, so that readers can readily verify the data and understand the basis for their use ***(please see the Supplementary Table S1).*** It is worth noting that the utility decrement for mild acute pulmonary exacerbation (APE) was derived by averaging the utility losses associated with respiratory symptoms such as cough and dyspnea[36], and the utility decrements for grade 2-4 RP were based on the utility losses associated with infectious pneumonitis[42].

**Comment 5. The model-generated APEFS rates in the pirfenidone and control groups are impressively close to the RCT data. Can the investigators clarify if the model parameters were tuned (calibrated) to fit these same data? If so, this should be framed as calibration rather than validation.**

**Response:** We sincerely thank the reviewer for this insightful comment and for highlighting the important distinction between model calibration and validation. We apologize if the description in the Methods section was not sufficiently clear and may have caused misunderstanding and we fully agree with the reviewer’s point.

In our analysis, the transition probability parameters were primarily derived directly from our RCT individual-level data. Therefore, the comparison between the model-generated survival data and the observed RCT survival data was not intended as an independent model validation. Rather, it is more appropriately interpreted as a model calibration, demonstrating that the model can reproduce the outcome observed in the RCT. Following the reviewer’s suggestion, we have revised the manuscript accordingly and have renamed the subsection as ‘Model Calibration’ in ***Methods and Results sections (please see the Methods section, line 193-201 on page10; please see the Results section, line 256-264 on page 14).***

Below we provide a more detailed description of the model calibration process. The specific calculation procedures of the baseline transition probabilities were conducted exactly as described in the Supplementary Methods, and these baseline parameters were not further tuned or fitted to reproduce the observed APEFS outcomes. After obtaining the baseline transition probabilities, we evaluated the performance of the Markov model from two aspects. First, we compared the model-simulated distribution of radiation pneumonitis (RP) grades with the observed distribution in the real-world follow-up data at four time points (week 4, week 8, week 16, and week 24). The simulated distributions were generated using the baseline transition parameters within the Markov model, while the observed distributions were directly calculated from the proportions of each RP grade in the real follow-up cohort. Second, we compared the model-simulated acute pulmonary exacerbation (APE) outcomes with those observed in the real-world cohort. In the model simulation, APE events occurring in the Monte Carlo–simulated virtual patient cohort were recorded, and the corresponding Kaplan–Meier curve for APEFS was generated. This simulated curve was then compared with the Kaplan–Meier curve derived directly from the observed APEFS data in the real-world cohort.

Based on the procedures described above, we assessed the consistency between the model outputs and observed outcomes. Therefore, this process represents **model calibration**. Following the reviewer’s suggestion, we have further clarified this process in the revised manuscript to avoid potential misunderstanding.

**Comment 6. Please define the difference between the dashed vs. solid arrows in the Markov model diagram (Figure 1).**

**Response:** We sincerely thank the reviewer for carefully examining the diagram of the Markov model. Considering that both the dashed and solid arrows in ***Figure 1*** represent transitions between health states, we have now standardized all arrows to solid lines in the revised figure. This change ensures a clearer and more uniform representation of the health state transitions and avoids any potential confusion for readers ***(please see the Figure 1 below).***

 **Fig 1. Diagram of Markov model.** Grade 2 or 3 RP patients treated with either pirfenidone plus glucocorticoids or glucocorticoids would enter the Markov model, where patients move between the health states based on prespecified 4-week transition probabilities. During the treatment period, patients may transition between RP states and are at risk of APE.

Abbreviations: RP, radiation pneumonitis; APE, acute pulmonary exacerbation.

**Comment 7. Please append the CHEERS Checklist.**

**Response:** We sincerely thank the reviewer for this helpful reminder. Following the reviewer’s suggestion, we have now appended the Consolidated Health Economic Evaluation Reporting Standards (CHEERS) Checklist in the ***Supplementary Materials*** to ensure full compliance with CHEERS reporting guidelines. Its inclusion further improves the transparency and completeness of our economic evaluation reporting ***(please see the Table S2 in the Supplementary Materials).***

**Comment 8. It appears the appended Lancet Oncology paper is now out. Congratulations on the publication! Please update the reference list to include it.**

**Response:** We sincerely thank the reviewer for this kind reminder. We have now updated the reference list to include the officially published version of the Lancet Oncology article ***(please see the Introduction section, line 110 on page 6).***

**Comment 9. Minor: In Table S1, there is an undefined acronym "MIAB" listed as the source for some of the CEA inputs. Please define this (perhaps Medial Insurance Administration Bureau of Guangzhou?).**

**Response:** We sincerely thank the reviewer for carefully identifying this issue. MIAB refers to the Medical Insurance Administration Bureau of Guangzhou, which serves as the source of several cost inputs used in the cost-effectiveness analysis. We have carefully revised both the supplementary table and the corresponding text to clearly define the acronym ***(please see Table S1).***

**General editorial requests:**

**Comment 1. We ask every co-author listed on the manuscript to fill in a contributing author statement, making sure to declare all competing interests. If any of the co-authors have not filled in the statement, we will remind them to do so when the paper is revised. If all statements are not completed in a timely fashion this could hold up the re-review process. If new competing interests are declared later in the revision process, this may also hold up the submission. Should there be a problem getting one of your co-authors to fill in a statement we will be in contact. Please do not add or remove authors without first discussing this with the handling editor. You can see our competing interests policy here:** [**http://journals.plos.org/plosmedicine/s/competing-interests**](http://journals.plos.org/plosmedicine/s/competing-interests)**.**

**Response:** We thank the editor for this important reminder. We confirm that all co-authors have completed the contributing author statements. We further declare that none of the authors has any competing interests to disclose. We also confirm that no authors have been added or removed during the revision process.

**Comment 2. Please upload any figures associated with your paper as individual TIF or EPS files with 300dpi resolution at resubmission; please read our figure guidelines for more information on our requirements: http://journals.plos.org/plosmedicine/s/figures. While revising your submission, we strongly recommend that you use PLOS's NAAS tool (https://ngplosjournals.pagemajik.ai/artanalysis) to test your figure files. NAAS can convert your figure files to the TIFF file type and meet basic requirements (such as print size, resolution), or provide you with a report on issues that do not meet our requirements and that NAAS cannot fix.
After uploading your figures to PLOS's NAAS tool - https://ngplosjournals.pagemajik.ai/artanalysis, NAAS will process the files provided and display the results in the "Uploaded Files" section of the page as the processing is complete.
If the uploaded figures meet our requirements (or NAAS is able to fix the files to meet our requirements), the figure will be marked as "fixed" above. If NAAS is unable to fix the files, a red "failed" label will appear above.
When NAAS has confirmed that the figure files meet our requirements, please download the file via the download option, and include these NAAS processed figure files when submitting your revised manuscript.**

**Response:** We thank the editorial office for the detailed instructions regarding figure submission and the use of the NAAS tool. We will ensure that all figures associated with our manuscript are uploaded as individual TIF or EPS files at 300 dpi resolution, in accordance with the journal’s figure guidelines. Prior to resubmission, we will process all figures using the PLOS NAAS tool to confirm compliance, and we will include the NAAS-processed files when submitting our revised manuscript.

**Comment 3. Please ensure that the paper adheres to the PLOS Data Availability Policy (see http://journals.plos.org/plosmedicine/s/data-availability), which requires that all data underlying the study's findings be provided in a repository or as Supporting Information. For data residing with a third party, authors are required to provide instructions with contact information (web or email address) for obtaining the data. Please note that a study author cannot be the contact person for the data. PLOS journals do not allow statements supported by "data not shown" or "unpublished results." For such statements, authors must provide supporting data or cite public sources that include it.**

**Response:** We thank the editor for the guidance. We confirm that the manuscript fully complies with the PLOS Data Availability Policy. All data underlying the findings are provided. We have ensured that all statements in the manuscript are supported by available data or public sources, and no references to “data not shown” or “unpublished results” remain.

**Comment 4. We expect all researchers with submissions to PLOS in which author-generated code underpins the findings in the manuscript to make all author-generated code available without restrictions upon publication of the work. In cases where code is central to the manuscript, we may require the code to be made available as a condition of publication. Authors are responsible for ensuring that the code is reusable and well documented. Please make any custom code available, either as part of your data deposition or as a supplementary file. Please add a sentence to your data availability statement regarding any code used in the study, e.g. "The code used in the analysis is available from Github [URL] and archived in Zenodo [DOI link]" Please review our guidelines at https://journals.plos.org/plosmedicine/s/materials-software-and-code-sharing and ensure that your code is shared in a way that follows best practice and facilitates reproducibility and reuse. Because Github depositions can
be readily changed or deleted, we encourage you to make a permanent DOI'd copy (e.g. in Zenodo) and provide the URL.**

**Response:** We thank the editor for the clarification. We confirm that this item is not applicable, as no author-generated code was used in the analyses reported in this manuscript.

**Comment 5. Please include the name and approval number from the ethics committee that approved your study.**

**Response:** We sincerely thank the editor for the reminder. We confirm that our study was approved by the institutional ethics committee of Sun Yat-sen University Cancer Center with the approval number (SL-B2021-336-02).

**Comment 6. Abstract: Please structure your abstract using the PLOS Medicine headings (Background, Methods and Findings, Conclusions). Please combine the Methods and Findings sections into one section.**

**Response:** We sincerely thank the editor for this suggestion. We have revised the abstract to follow the PLOS Medicine structured format, using the headings Background, Methods and Findings, and Conclusions.

**Comment 7. At this stage, we ask that you include a short, non-technical Author Summary of your research to make findings accessible to a wide audience that includes both scientists and non-scientists. The Author Summary should immediately follow the Abstract in your revised manuscript. This text is subject to editorial change and should be distinct from the scientific abstract. Ideally each sub-heading should contain 2-3 single sentence, concise bullet points containing the most salient points from your study. In the final bullet point of 'What Do These Findings Mean?', please include the main limitations of the study in non-technical language. Please see our author guidelines for more information:** [**https://journals.plos.org/plosmedicine/s/revising-your-manuscript#loc-author-summary**](https://journals.plos.org/plosmedicine/s/revising-your-manuscript#loc-author-summary)**.**

**Response:** We thank the editorial office for the guidance regarding the Author Summary. We have prepared a concise, non-technical Author Summary in accordance with PLOS Medicine guidelines. The Author Summary has been inserted immediately following the Abstract in the revised manuscript.

**Comment 8. Please express the main results with 95% CIs as well as p values. When reporting p values please report as p<0.001 and where higher as the exact p value p=0.002, for example. Throughout, suggest reporting statistical information as follows to improve clarity for the reader "22% (95% CI [13%,28%]; p</=)". Please be sure to define all numerical values at first use.**

**Response:** We thank the reviewer for this helpful suggestion. However, this item is not applicable to our study, as the data presented do not require additional 95% confidence intervals or p-value reporting beyond what has already been provided.

**Comment 9. Please include page numbers and line numbers in the manuscript file. Use continuous line numbers (do not restart the numbering on each page).**

**Response:** We thank the editor for this helpful suggestion. We have added continuous page and line numbers throughout the revised manuscript to facilitate review.

**Comment 10. Please cite the reference numbers in square brackets. Citations should precede punctuation.**

**Response:** We thank the editor for this helpful suggestion. We have revised the manuscript to cite all references in square brackets, with citations placed before punctuation throughout.

**Comment 11. Please provide titles and legends for all figures and tables (including those in Supporting Information files).**

**Response:** We thank the editor for this valuable suggestion. We have ensured that all figures and tables, including those in the Supporting Information files, now include complete titles and legends.

**Comment 12. Please define all abbreviations used in each figure/table (including those in Supporting Information files).**

**Response:** We thank the editor for this helpful suggestion. We have defined all abbreviations used in each figure and table, including those in the Supporting Information files.

**Comment 13. Please consider avoiding the use of red and green in order to make your figure more accessible to those with color blindness.**

**Response:** We thank the editor for this valuable suggestion. We have revised the figures to avoid the use of red and green, improving accessibility for readers with color vision deficiencies.

**Comment 14. Please note that supplementary material will be posted as supplied by the authors. Therefore, please amend it according to the relevant comments outlined here.**

**Response:** We thank the editor for this guidance. We have carefully revised the supplementary material according to the relevant comments to ensure it aligns with the manuscript and meets the journal’s requirements.

**Comment 15. Please note that supplementary material will be posted as supplied by the authors. Therefore, please amend it according to the relevant comments outlined here.**

**Response:** We thank the editor for the reminder and have updated our manuscript to cite Supporting Information files according to the journal’s guidelines.

**Comment 16. PLOS uses the numbered citation (citation-sequence) method and first six authors, et al.**

**Response:** We thank the editor for the clarification and have updated all references in the manuscript to follow PLOS’s numbered citation format, using “first six authors, et al.” where applicable.

**Comment 17. Please ensure that journal name abbreviations match those found in the National Center for Biotechnology Information (NCBI) databases (http://www.ncbi.nlm.nih.gov/nlmcatalog/journals), and are appropriately formatted and capitalised.**

**Response:** We thank the editor for the guidance and have carefully checked all journal name abbreviations in the manuscript. They have been updated to match the NCBI database, with correct formatting and capitalization.

**Comment 18. Please ensure that journal name abbreviations match those found in the National Center for Biotechnology Information (NCBI) databases (http://www.ncbi.nlm.nih.gov/nlmcatalog/journals), and are appropriately formatted and capitalised.**

**Response:** We thank the editor for the reminder and have updated all website citations in the manuscript to include the complete URLs along with the dates of access.

**Comment 19. Please ensure that journal name abbreviations match those found in the National Center for Biotechnology Information (NCBI) databases (http://www.ncbi.nlm.nih.gov/nlmcatalog/journals), and are appropriately formatted and capitalised.**

**Response:** We thank the editor for the guidance and have reviewed the reference formatting in accordance with the PLOS Medicine submission guidelines. All references have been updated accordingly.

**Comment 20. Please ensure that the study is reported according to the CHEERS guideline (available from: https://www.equator-network.org/reporting-guidelines/cheers) and include the completed checklist as Supporting Information. Please add the following statement, or similar, to the Methods: "This study is reported as per the Strengthening the Consolidated Health Economic Evaluation Reporting Standards 2022 (CHEERS 2022) Statement (S1 Checklist)." When completing the checklist, please use section and paragraph numbers, rather than page numbers.**

**Response:** We thank the editor for this helpful reminder. We have ensured that the study is reported in accordance with the CHEERS 2022 guidelines and have included the completed checklist as Supporting Information ***(please see the Supplementary Table S2).*** The following statement has been added to the Methods section: “This study is reported as per the Strengthening the Consolidated Health Economic Evaluation Reporting Standards 2022 (CHEERS 2022) Statement (S1 Checklist)” ***(please see the Methods section, line 191 on page 10).***

**REFERENCE**

1.	Komorowski M, Raffa J. Markov models and cost effectiveness analysis: applications in medical research. Secondary analysis of electronic health records. 2016:351-67.

2.	Pereira H, Fonseca RJ, Mouriño H. Application of Markov Models to Cost-Effectiveness Analysis in the Selection of Patients for Liver Transplantation. Mathematics. 2025;13(22):3683.

3.	Neilsen O. Direct Costing-The Case" For". The Accounting Review. 1954;29(1):89-93.

4.	Sonnenberg FA, Beck JR. Markov models in medical decision making: a practical guide. Medical decision making. 1993;13(4):322-38.

5.	Velikova T, Mileva N, Naseva E. Method “Monte Carlo” in healthcare. World Journal of Methodology. 2024;14(3):93930.

6.	Qaryan M, Kafian-Attari I, Afara IO. Monte Carlo Simulations of Light-Skin Interactions: Implications for Therapeutic and Oncological Applications. Photodiagnosis and Photodynamic Therapy. 2025:105251.

7.	Briggs AH. Handling uncertainty in cost-effectiveness models. Pharmacoeconomics. 2000;17(5):479-500.

8.	Briggs A, Sculpher M. An introduction to Markov modelling for economic evaluation. Pharmacoeconomics. 1998;13(4):397-409.

9.	Guo H, Yu R, Zhang H, Wang W. Cytokine, chemokine alterations and immune cell infiltration in Radiation-induced lung injury: implications for prevention and management. International Immunopharmacology. 2024;126:111263.

10.	Ryan KJ, Nero D, Feinberg BA, Lee CH, Pimentel R, Gajra A, et al. Real-world incidence and cost of pneumonitis post-chemoradiotherapy for stage III non-small-cell lung cancer. Future Oncology. 2019;16(1):4303-13.

11.	Macario A, Chow JL, Dexter F. A Markov computer simulation model of the economics of neuromuscular blockade in patients with acute respiratory distress syndrome. BMC Medical Informatics and Decision Making. 2006;6(1):15.

12.	Maunoury F, Motrunich A, Palka-Santini M, Bernatchez SF, Ruckly S, Timsit J-F. Cost-effectiveness analysis of a transparent antimicrobial dressing for managing central venous and arterial catheters in intensive care units. PLoS One. 2015;10(6):e0130439.

13.	Urban JA. Cost analysis of surgical site infections. Surgical infections. 2006;7(1_suppl):s19-s22.

14.	Polsky D, Glick H. Costing and cost analysis in randomized controlled trials: caveat emptor. Pharmacoeconomics. 2009;27(3):179-88.

15.	De Ruysscher D, Wauters E, Jendrossek V, Filippi AR, Revel M-P, Faivre-Finn C, et al. Diagnosis and treatment of radiation induced pneumonitis in patients with lung cancer: An ESTRO clinical practice guideline. Radiotherapy and Oncology. 2025;207:110837.

16.	Arroyo-Hernández M, Maldonado F, Lozano-Ruiz F, Muñoz-Montaño W, Nuñez-Baez M, Arrieta O. Radiation-induced lung injury: current evidence. BMC pulmonary medicine. 2021;21(1):9.

17.	Tan SS, Bakker J, Hoogendoorn ME, Kapila A, Martin J, Pezzi A, et al. Direct cost analysis of intensive care unit stay in four European countries: applying a standardized costing methodology. Value in Health. 2012;15(1):81-6.

18.	Neumann PJ. Costing and perspective in published cost-effectiveness analysis. Medical care. 2009;47(7_Supplement_1):S28-S32.

19.	Hu H, Zhou T, Gao J, Ou Y, Ma A, Wang P. Economic burden and influence factors among hospitalized children with bronchiolitis or pneumonia: a multiregional study in China. Frontiers in Public Health. 2024;12:1364854.

20.	Gao J, Fan J, Zhou H, Jit M, Wang P. Health-related quality of life and economic burden of childhood pneumonia in China: a multiregion study. BMJ Paediatrics Open. 2023;7(1):e002031.

21.	Malone DC, Berg NS, Claxton K, Garrison LP, IJzerman M, Marsh K, et al. International society for pharmacoeconomics and outcomes research comments on the American Society of Clinical Oncology value framework. Journal of Clinical Oncology. 2016;34(24):2936-7.

22.	Singh J. International society for pharmacoeconomics and outcomes research. Indian Journal of Pharmacology. 2006;38(5):376-7.

23.	Huang HY, Shi JF, Guo LW, Bai YN, Liao XZ, Liu GX, et al. Expenditure and financial burden for the diagnosis and treatment of colorectal cancer in China: a hospital‐based, multicenter, cross‐sectional survey. Cancer Communications. 2017;36(1):1-15.

24.	Li Q, Jiang W, Wang Q, Shen Y, Gao J, Sato KD, et al. Non-medical financial burden in tuberculosis care: a cross-sectional survey in rural China. Infectious diseases of poverty. 2016;5(1):5.

25.	Kirkland RS, Kole AJ, Batra H, Boggs DH, Spencer SA, Dobelbower MC, et al. Predictors of in-hospital death in patients with lung cancer admitted for acute radiation pneumonitis: A healthcare cost and utilization project (HCUP) analysis. Clinical Lung Cancer. 2021;22(5):e716-e22.

26.	[Internet]. PcPp. New York: PharmacyChecker; [cited 6 March 2026]. Avaliable from: <https://www.pharmacychecker.com/pirfenidone/#prices>.

27.	Pourrahmat M-M, Kim A, Kansal AR, Hux M, Pushkarna D, Fazeli MS, et al. Health state utility values by cancer stage: a systematic literature review. The European Journal of Health Economics. 2021;22(8):1275-88.

28.	Hu W, Zhou L, Chu J, Sun N, Chen X, Liu S, et al. Estimating population norms for the health-related quality of life of adults in southern Jiangsu Province, China. Scientific Reports. 2022;12(1):9906.

29.	Liu X, Zhou H, Wei J, Li M, Luo G, Naidoo N, et al. An occupational health survey on health utility and occupational diseases in Chinese university staff to inform cost-utility analysis. Frontiers in Public Health. 2023;10:1022344.

30.	Vedadi A, Shakik S, Brown MC, Lok BH, Shepherd FA, Leighl NB, et al. The impact of symptoms and comorbidity on health utility scores and health-related quality of life in small cell lung cancer using real world data. Quality of Life Research. 2021;30(2):445-54.

31.	Doherty M, Leung Y, Su J, Naik H, Patel D, Eng L, et al. Health utility scores from EQ-5D and health-related quality of life in patients with esophageal cancer: a real-world cross-sectional study. Diseases of the Esophagus. 2018;31(12):doy058.

32.	Kaur MN, Yan J, Klassen AF, David JP, Pieris D, Sharma M, et al. A systematic literature review of health utility values in breast cancer. Medical Decision Making. 2022;42(5):704-19.

33.	Clay E, Cristeau O, Chafaie R, Pinta A, Mazaleyrat B, Cottin V. Cost-effectiveness of pirfenidone compared to all available strategies for the treatment of idiopathic pulmonary fibrosis in France. Journal of market access & health policy. 2019;7(1):1626171.

34.	Richeldi L, Du Bois RM, Raghu G, Azuma A, Brown KK, Costabel U, et al. Efficacy and safety of nintedanib in idiopathic pulmonary fibrosis. New England Journal of Medicine. 2014;370(22):2071-82.

35.	Rinciog C, Diamantopoulos A, Gentilini A, Bondue B, Dahlqvist C, Froidure A, et al. Cost-effectiveness analysis of nintedanib versus pirfenidone in idiopathic pulmonary fibrosis in Belgium. PharmacoEconomics-open. 2020;4(3):449-58.

36.	Doyle S, Lloyd A, Walker M. Health state utility scores in advanced non-small cell lung cancer. Lung Cancer. 2008;62(3):374-80.

37.	Rinciog C, Watkins M, Chang S, Maher T, LeReun C, Esser D, et al. A cost-effectiveness analysis of nintedanib in idiopathic pulmonary fibrosis in the UK. Pharmacoeconomics. 2017;35(4):479-91.

38.	Loveman E, Copley VR, Colquitt JL, Scott DA, Clegg AJ, Jones J, et al. The effectiveness and cost-effectiveness of treatments for idiopathic pulmonary fibrosis: systematic review, network meta-analysis and health economic evaluation. BMC Pharmacology and Toxicology. 2014;15(1):63.

39.	Casali P, Licitra L, Constantini M, Santoro A, Viterbori P, Bajetta E, et al. Quality of life assessment and clinical decision-making. Annals of oncology. 1997;8(12):1207-11.

40.	Okai A, Elter B, Williams M. Understanding Diverse Caregiver Experiences and Their Impact on Caregiver Burden in Multiple Sclerosis. International Journal of MS Care. 2025;27(Q2):125-33.

41.	Masic I. Medical decision making-an overview. Acta Informatica Medica. 2022;30(3):230.

42.	Butler JR, McIntyre P, MacIntyre CR, Gilmour R, Howarth AL, Sander B. The cost-effectiveness of pneumococcal conjugate vaccination in Australia. Vaccine. 2004;22(9-10):1138-49.
