# Supplementary material for: Cost-effectiveness of pirfenidone for patients with cancer and grade 2 or grade 3 radiation-induced lung injury: A modeling study
Source: PLoS Med. 2026 Aug 5;23(8):e1004817. doi: 10.1371/journal.pmed.1004817 (PMC13440887; doi:10.1371/journal.pmed.1004817)
Supplement: Supplementary file 6 [file pmed.1004817.s007.docx]

Dear Dr. Starowicz,

Thank you for the opportunity to revise our manuscript “***Cost-Effectiveness of Pirfenidone for Cancer Patients with Grade 2 and Grade 3 Radiation-Induced Lung Injury: A Modelling Study* (PMEDICINE-D-25-03853R1)**”. We sincerely thank the reviewers for their time and insightful comments, which have significantly strengthened this work. Our point-by-point responses are provided below. Text has been added/modified, and all changes are indicated in the manuscript, with corresponding locations provided.

We thank the editorial office for the insightful comments and overall positive feedback on our work. We sincerely hope the revised manuscript, which is strengthened by the additional analyses, is now suitable for publication in ***PLOS Medicine*** and look forward to hearing from you.

Yours Sincerely,

Ming Chen, M.D.

Department of Radiation Oncology, Sun Yat-sen University Cancer Center, State Key Laboratory of Oncology in South China, Collaborative Innovation Center of Cancer Medicine. No.651, Dongfeng East Road, Guangzhou, Guangdong, 510060, P.R. China

Tel: 86-20-87340540

E-mail: [chenming@sysucc.org.cn](mailto:chenming@sysucc.org.cn)

**POINT-TO-POINT RESPONSE**

**Reviewer comments**

**RESPONSE ACCEPTABLE: I feel that the authors have done a reasonable job of addressing the low number of Grade 3 RILI events, which necessitated pooling Grade 2 and Grade 3 RILI. The authors' one-way sensitivity analyses did not take into account the correlation of cost and disutility, which increase together with greater disease severity, but I would imagine this would only strengthen the study's conclusion that the therapy is cost-effective.
RESPONSE ACCEPTABLE: Regarding the utility of lower-grade adverse events, I did not find the authors' initial response compelling (that other studies have ignored these, and they can be ignored for patients with severe illness). However, the additional analyses presented, benchmarked on Grade 1-2 AE disutility from literature, do appear convincing, as evidence that the cost-effectiveness conclusion is robust to AE (dis)utility. As a minor point, it might be useful to highlight in the Limitations that there was no direct quality-of-life measurement in the study, and therefore the AE disutility was benchmarked from literature not necessarily generalizable to the study population. However, they authors could point out that this is mitigated by the sensitivity analyses.**

**Response:** We sincerely appreciate the reviewer’s rigorous comments. We strongly agree that the source of AE disutility values should be explicitly acknowledged despite the validation of our cost-effectiveness conclusions through sensitivity analyses. Therefore, we have revised the **Discussion section** to adopt a more cautious interpretation, emphasizing that our AE disutility values were derived from published literature rather than patient-reported HRQoL data collected in our clinical study. (***please see the Discussion section, line 575-580 on page 30***).

**NEEDS REVISION: Regarding the WTP threshold, the World Health Organization has explicitly withdrawn its guidelines on using 3x the GDP. This was not an appropriate choice for benchmarking the China-specific WTP threshold and should be revised using modern WTP literature (e.g., benchmarked on the marginal productivity of the healthcare system or individuals' WTP for health as a proportion of income).**

**Response:** We sincerely thank the reviewer for the thoughtful comments regarding the selection of the willingness-to-pay (WTP) threshold. We acknowledge that the threshold of 3× GDP per capita has recently drawn warnings from the World Health Organization (WHO) staff, as it tends to yield a higher proportion of interventions deemed cost-effective and may lead to a loss of would‑be health gains from resources deployed for health[1-4]. In light of these concerns, the 2025 edition of the China guidelines for pharmacoeconomic evaluations has revised this WTP threshold[5]. It now tightens the upper bound of the WTP threshold to no more than 2× GDP per capita, drawing on published local empirical evidence and considering the Chinese population's health preferences and affordability. This revision better aligns the threshold with the realities of China's healthcare system and socioeconomic development.

Based on the updated China Guidelines for Pharmacoeconomic Evaluations, we selected 2× GDP per capita as the WTP threshold for each QALY gained, equivalent $27,680 per QALY. Notably, the base-case ICER for the pirfenidone regimen was $20,956 per QALY, falling below this China-specific WTP threshold, indicating favorable cost-effectiveness. Furthermore, the PSA based on 10,000 Monte Carlo simulations reinforced the findings, showing that 73.93% of the simulated samples had an ICER below the WTP threshold, with 43.24% representing a dominant strategy. Overall, the pirfenidone regimen maintained a clear economic advantage over glucocorticoids alone under this locally-relevant threshold.

We have integrated these findings into the **Clinical Scenario Analysis section** ***(please see the Results section, line 432-443 on page 22-23)***, and presented the PSA results in **Additional File: Fig S5** ***(please see Additional File: Fig S5 below).***

 **Fig S5. Scatter plot of probabilistic sensitivity analysis at at the China-specific WTP threshold.** Black line indicates threshold of WTP of $27,680 per QALY. Each point in scatterplot corresponds to one sample of parameter values. Points above reference line indicate higher incremental cost effectiveness ratio than threshold value. The red ellipse represents the 95% confidence ellipse around the mean incremental cost and incremental effectiveness.

Abbreviations: QALY, quality-adjusted life-year; WTP, willingness-to-pay.

section.

**General editorial requests**

**Comment 1. Please ensure that the paper adheres to the PLOS Data Availability Policy (see http://journals.plos.org/plosmedicine/s/data-availability), which requires that all data underlying the study's findings be provided in a repository or as Supporting Information. For data residing with a third party, authors are required to provide instructions with contact information for obtaining the data. PLOS journals do not allow statements supported by "data not shown" or "unpublished results." For such statements, authors must provide supporting data or cite public sources that include it.**

**Response:** We thank the editorial office for the guidance regarding the Data Availability. We have addressed this requirement by adding a dedicated "Availability of data and materials" section in the revised manuscript, which clearly describes how the data underlying this study can be accessed. (***please see the* Availability of data and materials *section, line 635-638 on page 33***)

**REFERENCE**

1.	Loganathan T, Ng C-W, Lee W-S, Hutubessy RC, Verguet S, Jit M. Thresholds for decision-making: informing the cost-effectiveness and affordability of rotavirus vaccines in Malaysia. Health policy and planning. 2018;33(2):204-14.

2.	Bertram MY, Lauer JA, De Joncheere K, Edejer T, Hutubessy R, Kieny M-P, et al. Cost–effectiveness thresholds: pros and cons. Bulletin of the World Health Organization. 2016;94(12):925.

3.	Woods B, Revill P, Sculpher M, Claxton K. Country-level cost-effectiveness thresholds: initial estimates and the need for further research. Value in health. 2016;19(8):929-35.

4.	Robinson LA, Hammitt JK, Chang AY, Resch S. Understanding and improving the one and three times GDP per capita cost-effectiveness thresholds. Health Policy and Planning. 2017;32(1):141-5.

5.	Wu J, Liu GG. Comments on the 2025 Edition of China Guidelines for Pharmacoeconomic Evaluations. Pharmacoeconomics and Policy. 2026;2(1):1-3.
