# Supplementary material for: Cost-effectiveness of pirfenidone for patients with cancer and grade 2 or grade 3 radiation-induced lung injury: A modeling study
Source: PLoS Med. 2026 Aug 5;23(8):e1004817. doi: 10.1371/journal.pmed.1004817 (PMC13440887; doi:10.1371/journal.pmed.1004817)
Supplement: Peer review history

# Editor report: Decision Letter 0

**DOI:** 10.1371/journal.pmed.1004817.r001

**Authors:** Alexandra Tosun

3 Nov 2025

Dear Dr Chen,

Thank you for submitting your manuscript entitled "Cost-Effectiveness Analysis of Pirfenidone for Cancer Patients with Grade 2 and Grade 3 Radiation-Induced Lung Injury" for consideration by PLOS Medicine.

Your manuscript has now been evaluated by the PLOS Medicine editorial staff, and I am writing to let you know that we would like to send your submission out for external peer review.

However, before we can send your manuscript to reviewers, we need you to complete your submission by providing the metadata that is required for full assessment. To this end, please login to Editorial Manager where you will find the paper in the 'Submissions Needing Revisions' folder on your homepage. Please click 'Revise Submission' from the Action Links and complete all additional questions in the submission questionnaire.

For clinical studies, please upload a copy of your trial study protocol as a supporting information file. The study protocol should be the version submitted for approval to the institutional review board or ethics committee, should include any amendments to the study protocol, as well as the date of their approval by the institutional review or ethics committee. Please also detail any deviations from the study protocol in the Methods section of your manuscript. The editors will consider the protocol and study conduct prior to a final decision for external review.

Please re-submit your manuscript within two working days, i.e. by Nov 05 2025.

Login to Editorial Manager here: https://www.editorialmanager.com/pmedicine

Once your full submission is complete, your paper will undergo a series of checks in preparation for peer review. Once your manuscript has passed all checks it will be sent out for review.

Feel free to email me at atosun@plos.org or us at plosmedicine@plos.org if you have any queries relating to your submission.

Kind regards,

Alexandra Tosun, PhD

Senior Editor

PLOS Medicine

# Peer review: Decision Letter 1

**DOI:** 10.1371/journal.pmed.1004817.r002

**Authors:** Alexandra Tosun

24 Feb 2026

Dear Dr Chen,

Many thanks for submitting your manuscript "Cost-Effectiveness Analysis of Pirfenidone for Cancer Patients with Grade 2 and Grade 3 Radiation-Induced Lung Injury" (PMEDICINE-D-25-03853R1) to PLOS Medicine. The paper has been reviewed by subject experts and a statistician; their comments are included below and can also be accessed here: [LINK]

As you will see, the reviewers are mostly supportive of your manuscript but have raised several concerns. For example, they have questioned the sources of the included values and the fact that the model's time horizon is set at 24 weeks. After discussing the paper with the editorial team and an academic editor with relevant expertise, I'm pleased to invite you to revise the paper in response to the reviewers' comments. We plan to send the revised paper to some or all of the original reviewers, and we cannot provide any guarantees at this stage regarding publication.

In addition to these revisions, you may need to complete some formatting changes, which you will receive in a follow up email. A member of our team will be in touch with a set of requests shortly. If you do not receive a separate email within a few days, please assume that checks have been completed, and no additional changes are required.

When you upload your revision, please include a point-by-point response that addresses all of the reviewer and editorial points, indicating the changes made in the manuscript and either an excerpt of the revised text or the location (eg: page and line number) where each change can be found. Please also be sure to check the general editorial comments at the end of this letter and include these in your point-by-point response. When you resubmit your paper, please include a clean version of the paper as the main article file and a version with changes tracked as a marked-up manuscript. It may also be helpful to check the guidelines for revised papers at http://journals.plos.org/plosmedicine/s/revising-your-manuscript for any that apply to your paper.

We ask that you submit your revision by Mar 17 2026. However, if this deadline is not feasible, please contact me by email, and we can discuss a suitable alternative.

Don't hesitate to contact me directly with any questions (atosun@plos.org).

Best regards,

Alexandra

Alexandra Tosun, PhD

Senior Editor

PLOS Medicine

atosun@plos.org

-----------------------------------------------------------

Comments from the reviewers:

Reviewer #1: This is an interesting Cost-Effectiveness Analysis on Pirfenidone for Cancer Patients with Grade 2 and Grade 3 Radiation-Induced Lung Injury. However, there are some major issues needing attention.

1. There is no clear justification why Markov simulations were used for analyses in the first place. Why not direct costing analysis? It's well known that Markov simulation Analysis is a dynamic model that simulates transitions between discrete health states over many cycles, often representing lifetime horizons. However, in this study the follow up time is only 24 weeks, not that long. Plus, there are issues with Markov model approach such as very complex, data-intensive, lower transparency and difficult to follow.

2. Could authors consider to combine both direct costing and Markov simulation in the study, so that one can get more credible cost estimates, improved transparency, making the Markov model more realistic, addressing both short-term and long-term perspectives in one paper.

Reviewer #2: This is a well-designed and clinically significant cost-effectiveness analysis. The study evaluates the economic value of adding pirfenidone to standard glucocorticoids for grade 2-3 radiation-induced lung injury, based on the authors' own Phase II RCT data. The results demonstrate a significant cost-effectiveness advantage for the combination strategy. The overall quality of the manuscript is high, and it provides valuable insights for clinical practice and healthcare policy. I have the following comments:

1. According to the official methodological guidelines of the International Society for Pharmacoeconomics and Outcomes Research (ISPOR) regarding the definition of a "societal perspective," the manuscript currently only mentions transportation expenses and wage loss due to work absenteeism. However, other relevant societal costs, such as caregiver services, special diets, and other indirect costs, are not addressed. Further clarification on the comprehensiveness of the societal cost calculation is needed.

2. The price of pirfenidone is based on the 2025 Guangzhou medical insurance negotiation price, yet the conclusion suggests "global applicability." To strengthen the persuasiveness of this argument, I recommend supplementing the analysis with international reference prices or explicitly discussing this as a limitation regarding the generalizability of the findings.

3. The baseline health utility value of 0.712 is sourced from a 2008 study involving a predominantly White, UK-based Western population with advanced NSCLC. Applying this value directly to the current study set in China raises questions about potential differences due to ethnicity or cross-cultural health preferences. The authors should address and justify this choice.

4. The model's time horizon is set at 24 weeks, covering only the acute inflammatory phase of RILI. However, the core disabling and life-threatening risks of RILI stem from its chronic, progressive fibrotic phase, which can last for years. As an anti-fibrotic agent, pirfenidone's primary clinical and economic value likely lies in modifying this long-term process. Although the authors have mentioned this as a limitation, even in the absence of long-term RCT data, constructing a simple extrapolation model that includes states for long-term fibrosis progression and conducting scenario analyses would be valuable. It is recommended to add a paragraph in the "Discussion" section elaborating on how future analyses could be improved to address this.

5. Nintedanib, like pirfenidone, is an anti-fibrotic drug approved for idiopathic pulmonary fibrosis and progressive fibrosing interstitial lung diseases. While the conclusion that "pirfenidone plus glucocorticoids is cost-effective" is valid within the framework of this study, the assertion that it "should become the standard treatment" is too strong and insufficiently supported by the evidence. It is recommended to add a discussion of this limitation in the "Discussion" section and modify the wording in the conclusion accordingly.

6. While cost-effectiveness analyses typically employ QALYs, clinical decision-making often incorporates other "values," such as a treatment's impact on a patient's ability to return to work, caregiver burden, or the influence of complications. The discussion could briefly acknowledge that the present study did not capture these dimensions, which could be a direction for future research.

7. In Supplementary Materials, the title of Table S3 is positioned below the table. Please adjust the formatting accordingly.

Reviewer #3:

Thank you for the opportunity to review this cost-effectiveness analysis of pirfenidone for radiation-induced lung injury (RILI).

This study used robust methodology including a mechanistic Markov model, univariate sensitivity analysis, multivariate PSA, and a CEAC, all of which strengthen the conclusion of very probable cost-effectiveness of the pirfenidone+glucocordicoids strategy. My suggestions are relatively minor.

Net health benefit (NHB) and incremental cost-effectiveness are co-primary outcomes, but the Discussion focuses only on ICER. Please also discuss the NHB outcome and whether the result concurred with the hypothesis of the study. For example, NHB could be contextualized by comparing it to NHB benchmarks for other health interventions.

In the supplementary ensitivity analysis, it appears the investigators separately/independently altered disease progression parameters in the pirfenidone group vs. the glucocorticoids group (Figure S2 panel A vs. B). This approach appears unjustified, given that the trial randomly assigned pirfenidone, and the study conceptualizes pirfenidone + glucocordicoids as an alternative scenario to glucocordicoids alone in the same population. If a disease progression parameter were different from the base case (e.g., a progression rate or utility were overestimated), it would differ by the same amount in both the pirfenidone+glucocordicoids and the glucocordicoids groups. These parameters should be changed by the same amount in both groups before calculating the resulting ICER.

Quite a few cost items related to RILI healthcare do not have a source/reference provided. Where were these values obtained? Also, for transparency, please disaggregate cost line items into unit cost and number of units, if possible.

For utility values, the references listed do not appear to be the source studies that performed time trade-off or other experiments to estimate utility of RILI grades or of the other conditions used to benchmark RILI symptom (dis)utility. Please revise the reference list to indicate the sources for the assumed utility values.

The model-generated APEFS rates in the pirfenidone and control groups are impressively close to the RCT data. Can the investigators clarify if the model parameters were tuned (calibrated) to fit these same data? If so, this should be framed as calibration rather than validation.

Please define the difference between the dashed vs. solid arrows in the Markov model diagram (Figure 1).

Please append the CHEERS Checklist.

It appears the appended Lancet Oncology paper is now out. Congratulations on the publication! Please update the reference list to include it.

Minor: In Table S1, there is an undefined acronym "MIAB" listed as the source for some of the CEA inputs. Please define this (perhaps Medial Insurance Administration Bureau of Guangzhou?).

--------------------------------------------------------- ---

General editorial requests:

(Note: not all will apply to your paper, but please check each item carefully)

*We ask every co-author listed on the manuscript to fill in a contributing author statement, making sure to declare all competing interests. If any of the co-authors have not filled in the statement, we will remind them to do so when the paper is revised. If all statements are not completed in a timely fashion this could hold up the re-review process. If new competing interests are declared later in the revision process, this may also hold up the submission. Should there be a problem getting one of your co-authors to fill in a statement we will be in contact. Please do not add or remove authors without first discussing this with the handling editor. You can see our competing interests policy here: http://journals.plos.org/plosmedicine/s/competing-interests.

*Please upload any figures associated with your paper as individual TIF or EPS files with 300dpi resolution at resubmission; please read our figure guidelines for more information on our requirements: http://journals.plos.org/plosmedicine/s/figures. While revising your submission, we strongly recommend that you use PLOS's NAAS tool (https://ngplosjournals.pagemajik.ai/artanalysis) to test your figure files. NAAS can convert your figure files to the TIFF file type and meet basic requirements (such as print size, resolution), or provide you with a report on issues that do not meet our requirements and that NAAS cannot fix.

After uploading your figures to PLOS's NAAS tool - https://ngplosjournals.pagemajik.ai/artanalysis, NAAS will process the files provided and display the results in the "Uploaded Files" section of the page as the processing is complete.

If the uploaded figures meet our requirements (or NAAS is able to fix the files to meet our requirements), the figure will be marked as "fixed" above. If NAAS is unable to fix the files, a red "failed" label will appear above.

When NAAS has confirmed that the figure files meet our requirements, please download the file via the download option, and include these NAAS processed figure files when submitting your revised manuscript.

*Please ensure that the paper adheres to the PLOS Data Availability Policy (see http://journals.plos.org/plosmedicine/s/data-availability), which requires that all data underlying the study's findings be provided in a repository or as Supporting Information. For data residing with a third party, authors are required to provide instructions with contact information (web or email address) for obtaining the data. Please note that a study author cannot be the contact person for the data. PLOS journals do not allow statements supported by "data not shown" or "unpublished results." For such statements, authors must provide supporting data or cite public sources that include it.

*We expect all researchers with submissions to PLOS in which author-generated code underpins the findings in the manuscript to make all author-generated code available without restrictions upon publication of the work. In cases where code is central to the manuscript, we may require the code to be made available as a condition of publication. Authors are responsible for ensuring that the code is reusable and well documented. Please make any custom code available, either as part of your data deposition or as a supplementary file. Please add a sentence to your data availability statement regarding any code used in the study, e.g. "The code used in the analysis is available from Github [URL] and archived in Zenodo [DOI link]" Please review our guidelines at https://journals.plos.org/plosmedicine/s/materials-software-and-code-sharing and ensure that your code is shared in a way that follows best practice and facilitates reproducibility and reuse. Because Github depositions can be readily changed or deleted, we encourage you to make a permanent DOI'd copy (e.g. in Zenodo) and provide the URL.

*Please include the name and approval number from the ethics committee that approved your study.

FORMATTING - GENERAL

*Abstract: Please structure your abstract using the PLOS Medicine headings (Background, Methods and Findings, Conclusions). Please combine the Methods and Findings sections into one section.

*At this stage, we ask that you include a short, non-technical Author Summary of your research to make findings accessible to a wide audience that includes both scientists and non-scientists. The Author Summary should immediately follow the Abstract in your revised manuscript. This text is subject to editorial change and should be distinct from the scientific abstract. Ideally each sub-heading should contain 2-3 single sentence, concise bullet points containing the most salient points from your study. In the final bullet point of 'What Do These Findings Mean?', please include the main limitations of the study in non-technical language. Please see our author guidelines for more information: https://journals.plos.org/plosmedicine/s/revising-your-manuscript#loc-author-summary.

*Please express the main results with 95% CIs as well as p values. When reporting p values please report as p<0.001 and where higher as the exact p value p=0.002, for example. Throughout, suggest reporting statistical information as follows to improve clarity for the reader "22% (95% CI [13%,28%]; p</=)". Please be sure to define all numerical values at first use.

*Please include page numbers and line numbers in the manuscript file. Use continuous line numbers (do not restart the numbering on each page).

*Please cite the reference numbers in square brackets. Citations should precede punctuation.

FIGURES AND TABLES

*Please provide titles and legends for all figures and tables (including those in Supporting Information files).

*Please define all abbreviations used in each figure/table (including those in Supporting Information files).

*Please consider avoiding the use of red and green in order to make your figure more accessible to those with color blindness.

SUPPLEMENTARY MATERIAL

*Please note that supplementary material will be posted as supplied by the authors. Therefore, please amend it according to the relevant comments outlined here.

*Please cite your Supporting Information as outlined here: https://journals.plos.org/plosmedicine/s/supporting-information

REFERENCES

*PLOS uses the numbered citation (citation-sequence) method and first six authors, et al.

*Please ensure that journal name abbreviations match those found in the National Center for Biotechnology Information (NCBI) databases (http://www.ncbi.nlm.nih.gov/nlmcatalog/journals), and are appropriately formatted and capitalised.

*Where website addresses are cited, please include the complete URL and specify the date of access (e.g. [accessed: 12/06/2023]).

*Please also see https://journals.plos.org/plosmedicine/s/submission-guidelines#loc-references for further details on reference formatting.

STUDY TYPE-SPECIFIC REQUESTS

*Please ensure that the study is reported according to the CHEERS guideline (available from: https://www.equator-network.org/reporting-guidelines/cheers) and include the completed checklist as Supporting Information. Please add the following statement, or similar, to the Methods: "This study is reported as per the Strengthening the Consolidated Health Economic Evaluation Reporting Standards 2022 (CHEERS 2022) Statement (S1 Checklist)." When completing the checklist, please use section and paragraph numbers, rather than page numbers.

# Author comment: Author response to Decision Letter 2

**DOI:** 10.1371/journal.pmed.1004817.r003

17 Mar 2026

Attachment Submitted filename: Response to reviewers.docx

# Peer review: Decision Letter 2

**DOI:** 10.1371/journal.pmed.1004817.r004

**Authors:** Katarzyna Starowicz

13 May 2026

Dear Dr. Chen,

Thank you very much for re-submitting your manuscript "Cost-Effectiveness Analysis of Pirfenidone for Cancer Patients with Grade 2 and Grade 3 Radiation-Induced Lung Injury" (PMEDICINE-D-25-03853R2) for review by PLOS Medicine.

I have discussed the paper with my colleagues and the academic editor and it was also seen again by three reviewers. There are still remaining concerns from reviewers that need to be addressed in full, they are listed at the end of this email. I have also included editorial requests, you must provide point-by-point response to all of them. Some of them might not be applicable to your study, in that case please respond "not applicable". Not providing the response to editorial requests will significantly delay your manuscript. If any of them have been already addressed in previous revision please indicate that in your response.

Any accompanying reviewer attachments can be seen via the link below. Please take these into account before resubmitting your manuscript:

[LINK]

***Please note while forming your response, if your article is accepted, you may have the opportunity to make the peer review history publicly available. The record will include editor decision letters (with reviews) and your responses to reviewer comments. If eligible, we will contact you to opt in or out.***

In revising the manuscript for further consideration here, please ensure you address the specific points made by each reviewer and the editors. In your rebuttal letter you should indicate your response to the reviewers' and editors' comments and the changes you have made in the manuscript. Please submit a clean version of the paper as the main article file. A version with changes marked must also be uploaded as a marked up manuscript file.

Please also check the guidelines for revised papers at http://journals.plos.org/plosmedicine/s/revising-your-manuscript for any that apply to your paper. If you haven't already, we ask that you provide a short, non-technical Author Summary of your research to make findings accessible to a wide audience that includes both scientists and non-scientists. The Author Summary should immediately follow the Abstract in your revised manuscript. This text is subject to editorial change and should be distinct from the scientific abstract.

In addition to these revisions, you may need to complete some formatting changes, which you will receive in a follow up email. A member of our team will be in touch with a set of requests shortly. If you do not receive a separate email within a few days, please assume that checks have been completed, and no additional changes are required.

We expect to receive your revised manuscript within 1 week. Please email us (plosmedicine@plos.org) if you have any questions or concerns.

We ask every co-author listed on the manuscript to fill in a contributing author statement. If any of the co-authors have not filled in the statement, we will remind them to do so when the paper is revised. If all statements are not completed in a timely fashion this could hold up the re-review process. Should there be a problem getting one of your co-authors to fill in a statement we will be in contact. YOU MUST NOT ADD OR REMOVE AUTHORS UNLESS YOU HAVE ALERTED THE EDITOR HANDLING THE MANUSCRIPT TO THE CHANGE AND THEY SPECIFICALLY HAVE AGREED TO IT.

Please ensure that the paper adheres to the PLOS Data Availability Policy (see http://journals.plos.org/plosmedicine/s/data-availability), which requires that all data underlying the study's findings be provided in a repository or as Supporting Information. For data residing with a third party, authors are required to provide instructions with contact information for obtaining the data. PLOS journals do not allow statements supported by "data not shown" or "unpublished results." For such statements, authors must provide supporting data or cite public sources that include it.

To enhance the reproducibility of your results, we recommend that you deposit your laboratory protocols in protocols.io, where a protocol can be assigned its own identifier (DOI) such that it can be cited independently in the future. Additionally, PLOS ONE offers an option to publish peer-reviewed clinical study protocols. Read more information on sharing protocols at https://plos.org/protocols?utm_medium=editorial-email&utm_source=authorletters&utm_campaign=protocols

Please review your reference list to ensure that it is complete and correct. If you have cited papers that have been retracted, please include the rationale for doing so in the manuscript text, or remove these references and replace them with relevant current references. Any changes to the reference list should be mentioned in the rebuttal letter that accompanies your revised manuscript.

Please note, when your manuscript is accepted, an uncorrected proof of your manuscript will be published online ahead of the final version, unless you've already opted out via the online submission form. If, for any reason, you do not want an earlier version of your manuscript published online or are unsure if you have already indicated as such, please let the journal staff know immediately at plosmedicine@plos.org.

If you have any questions in the meantime, please contact me or the journal staff on plosmedicine@plos.org.

We look forward to receiving the revised manuscript by May 22 2026 11:59PM.

Sincerely,

Katarzyna Starowicz, PhD

Associate Editor

PLOS Medicine

kstarowicz@plos.org

------------------------------------------------------------

Requests from Editors:

* At this stage, we ask that you include a short, non-technical Author Summary of your research to make findings accessible to a wide audience that includes both scientists and non-scientists. The Author Summary should immediately follow the Abstract in your revised manuscript. This text is subject to editorial change and should be distinct from the scientific abstract. Ideally each sub-heading should contain 2-3 single sentence, concise bullet points containing the most salient points from your study. In the final bullet point of ‘What Do These Findings Mean?’ Please include the main limitations of the study in non-technical language.

Please see our author guidelines for more information: https://journals.plos.org/plosmedicine/s/revising-your-manuscript#loc-author-summary."

* Please confirm that your title complies with PLOS Medicine's style. Your title must be nondeclarative and not a question. It should begin with main concept if possible. "Effect of" should be used only if causality can be inferred, i.e., for an RCT. Please place the study design ("A randomized controlled trial," "A retrospective study," "A modelling study," etc.) in the subtitle (ie, after a colon).

* Please confirm that your abstract complies with our requirements, including format (three sections: Background, Methods and Findings, and Conclusions) and providing all the information relevant to this study type https://journals.plos.org/plosmedicine/s/submission-guidelines#loc-abstract

* Please ensure that the Introduction ends with a clear description of the study question or hypothesis.

* Please ensure that all abbreviations are defined at first use throughout the text.

* Please confirm that all numbers presented in the abstract are present and identical to numbers presented in the main manuscript text.

* The funding statement should include: specific grant numbers, initials of authors who received each award, URLs to sponsors’ websites. Also, please state whether any sponsors or funders (other than the named authors) played any role in study design, data collection and analysis, the decision to publish, or preparation of the manuscript. If they had no role in the research, include this sentence: “The funders had no role in study design, data collection and analysis, decision to publish, or preparation of the manuscript.”

* All authors must declare their relevant competing interests per the PLOS policy, which can be seen here: https://journals.plos.org/plosmedicine/s/competing-interests For authors with ties to industry, please indicate whether any of the interests has a financial stake in the results of the current study.

*Please amend your competing interests statement. Funding information should go to the relevant section and include URLs.

* Please review your text for claims of novelty or primacy (e.g. 'for the first time') and remove this language. In addition, please check that any use of statistical terms (such as trend or significant) are supported by the data, and if not please remove them.

* Please remove the 'conclusions' subheading from the discussion. Please also remove any other subheadings from the discussion.

* Statistical reporting: Please revise throughout the manuscript, including tables and figures.

- Please report statistical information as follows to improve clarity for the reader ""22% (95% CI [13,28]; p</=)"".

- Please separate upper and lower bounds with commas instead of hyphens as the latter can be confused with reporting of negative values.

- Please repeat statistical definitions (HR, CI etc.) for each set of parentheses."

* In the last sentence of the Abstract Methods and Findings section, please describe the main limitation(s) of the study's methodology.

* In the abstract, please include the important dependent variables that are adjusted for in the analyses.

* In the author summary, please revise formatting and ensure you use bullet points.

* In the author summary, in the final bullet point of 'What Do These Findings Mean?', please include the main limitations of the study in non-technical language.

* PLOS Medicine requires that the de-identified data underlying the specific results in a published article be made available, without restrictions on access, in a public repository or as Supporting Information at the time of article publication, provided it is legal and ethical to do so. Please see the policy at

http://journals.plos.org/plosmedicine/s/data-availability

and FAQs at

http://journals.plos.org/plosmedicine/s/data-availability#loc-faqs-for-data-policy "

* PLOS defines the “minimal data set” to consist of the data set used to reach the conclusions drawn in the manuscript with related metadata and methods, and any additional data required to replicate the reported study findings in their entirety. Authors do not need to submit their entire data set, or the raw data collected during an investigation. Please submit the following data:

The values behind the means, standard deviations and other measures reported;

The values used to build graphs;

The points extracted from images for analysis."

* Please provide the full name(s) of the institutional review board(s) that provided ethical approval.

* Figures cannot be reproduced from other sources that are not CC-BY. Please provide a different figure.

* Please provide titles and legends for all figures and tables (including those in Supporting Information files). Please define all acronyms used in each figure or table in its corresponding legend.

* Please ensure that where relevant figures include 95% CIs.

* Please show graph axes beginning at zero. If this is not possible, please show a break in the axis.

* When a p value is given, please specify the statistical test used to determine it in the legend.

* Please provide the unadjusted comparisons as well as the adjusted comparisons in all relevant Tables

* Please specify the variables controlled for in all relevant Tables

* Please consider if moving some of the figures shared in the supplementary information into the main text would aid the reader.

* Please include an Acknowledgments section in your manuscript.

* Please include an acknowledgment of study participants.

* Please ensure that the study is reported according to the CHEERS guideline (available from: https://www.equatornetwork.org/reporting-guidelines/cheers) and include the completed checklist as Supporting Information. Please add the following statement, or similar, to the Methods: "This study is reported as per the

Strengthening the Consolidated Health Economic Evaluation Reporting Standards 2022 (CHEERS 2022) Statement (S1 Checklist)." When completing the checklist, please use section and paragraph numbers, rather than page numbers.

-----------------

Comments from Reviewers:

Reviewer #1: Thanks authors for their great effort to improve the manuscript. The authors have addressed my comments well in a professional way. I am satisfied with the response and revision. No further issues needing attention.

Reviewer #2: I thank the authors for their careful responses to the previous comments and for the substantial revisions made to the manuscript. The revised version has improved in terms of model transparency, discussion of generalizability, and the wording of the conclusions. Overall, this study addresses an important clinical and health economic question by evaluating the economic value of adding pirfenidone to glucocorticoids for the treatment of grade 2 or 3 radiation-induced lung injury (RILI), based on a phase II randomized controlled trial. However, several methodological and reporting issues still require further clarification.

1. The estimation of utility values and the simplification of health states require further discussion. Because the number of patients with grade 3 radiation pneumonitis (RP) was limited, the authors combined grade 2 and grade 3 RP into a single health state to improve the statistical robustness of the model parameters. This approach is understandable from a statistical perspective. However, from clinical and economic perspectives, grade 2 and grade 3 RP may differ substantially in respiratory support, medications for infection, healthcare resource utilization, and utility decrement. For example, patients with grade 2 RP may usually receive low-flow oxygen support and empirical antibiotics when infection is suspected, whereas patients with grade 3 RP may require high-flow oxygen therapy or non-invasive ventilation, as well as more intensive anti-infective and anti-inflammatory management. Therefore, combining these two grades may obscure clinically meaningful heterogeneity and potentially underestimate the cost and utility burden among patients with grade 3 RP. The authors should discuss the potential impact of this assumption and, if feasible, perform a clinical scenario analysis assigning higher costs and larger utility decrements to grade 3 RP.

In addition, treatment-related toxicity was not incorporated into the model because the incidence of grade 3 or higher adverse events was comparable between groups. However, QALY estimates may also be affected by lower-grade adverse events. Pirfenidone-related gastrointestinal discomfort, rash, and other adverse events may be classified as low-grade but may persist over time and affect patients' quality of life and treatment adherence. The authors should further clarify why only grade 3 or higher adverse events were considered relevant sources of treatment-related disutility and consider incorporating treatment-related disutility in sensitivity or scenario analyses to evaluate the impact of this assumption on QALY and ICER results.

2. I appreciate the authors' response to the previous comment regarding the international reference prices of pirfenidone and its generalizability. However, a related issue still requires further clarification. The willingness-to-pay threshold is closely associated with the local economic context, income level, reimbursement system, and healthcare affordability. Therefore, although the supplemented international price comparison helps address the generalizability of drug costs, it does not fully justify the use of $100,000 per QALY as the main decision threshold for a China-based cost-effectiveness analysis.

Moreover, in the Results section, the interpretation of cost-effectiveness remains largely centered on the $100,000 per QALY threshold. Although the cost-effectiveness acceptability curve suggests that pirfenidone becomes more likely to be cost-effective when the WTP threshold exceeds $6,681 per QALY, the authors do not systematically report the probability of being cost-effective under China-relevant WTP thresholds. The authors should provide additional scenario analyses using China-specific thresholds. This would allow readers to better assess whether pirfenidone plus glucocorticoids remains cost-effective within the healthcare and economic context from which the cost data were derived.

3. The Abstract reports that pirfenidone plus glucocorticoids increased costs by $590 compared with glucocorticoids alone, whereas the Base-Case Analysis section reports an incremental cost of $595. The authors should check these values and clarify whether this discrepancy is due to a typographical error or rounding. I also suggest that the authors review the entire manuscript and tables to ensure that there are no similar inconsistencies or misreported data.

4. The authors should clarify how immunotherapy exposure and immune-related pneumonitis were handled. Some patients may have received immunotherapy before, during, or after radiotherapy. Immune-related pneumonitis may overlap with RP in both clinical presentation and imaging findings, and may also influence glucocorticoid treatment strategies and related treatment costs. The manuscript does not clearly report immunotherapy use among patients enrolled in the phase II RCT. The authors should report the proportion and balance of immunotherapy exposure between the two treatment groups and explain how RP was distinguished from immune-related pneumonitis. If such data are unavailable, this should be discussed as a limitation, particularly regarding the potential impact of immunotherapy exposure on model results and generalizability.

Reviewer #3: Regarding Reviewer 3's Comment 5, upon reading the author's more detailed description of the process for comparing model outputs to data, it is not clear that this process involved calibration. It appears no model parameters were tuned to bring model outputs closer to observed data. If the authors agree, they can revert the change that was made. They do not need to refer to this process as "model calibration" as this reviewed had previously, seemingly erroneously suggested.

All other comments were thoroughly addressed. Thank you, it was a pleasure to review this work.

Any attachments provided with reviews can be seen via the following link:

[LINK]

# Author comment: Author response to Decision Letter 3

**DOI:** 10.1371/journal.pmed.1004817.r005

22 May 2026

Attachment Submitted filename: Response_to_reviewers_auresp_3.docx

# Editor report: Decision Letter 3

**DOI:** 10.1371/journal.pmed.1004817.r006

**Authors:** Katarzyna Starowicz

19 Jun 2026

Dear Dr. Chen,

Thank you very much for re-submitting your manuscript "Cost-Effectiveness of Pirfenidone for Cancer Patients with Grade 2 and Grade 3 Radiation-Induced Lung Injury: A Modelling Study" (PMEDICINE-D-25-03853R3) for review by PLOS Medicine.

I have discussed the paper with my colleagues and the academic editor and it was also seen again by one reviewer. Reviewer feels that you did not address remaining concerns in full and we have decided to give you the opportunity to do it.

The remaining issues that need to be addressed are listed at the end of this email. Any accompanying reviewer attachments can be seen via the link below. Please take these into account before resubmitting your manuscript:

[LINK]

***Please note while forming your response, if your article is accepted, you may have the opportunity to make the peer review history publicly available. The record will include editor decision letters (with reviews) and your responses to reviewer comments. If eligible, we will contact you to opt in or out.***

In revising the manuscript for further consideration here, please ensure you address the specific points made by each reviewer and the editors. In your rebuttal letter you should indicate your response to the reviewers' and editors' comments and the changes you have made in the manuscript. Please submit a clean version of the paper as the main article file. A version with changes marked must also be uploaded as a marked up manuscript file.

Please also check the guidelines for revised papers at http://journals.plos.org/plosmedicine/s/revising-your-manuscript for any that apply to your paper. If you haven't already, we ask that you provide a short, non-technical Author Summary of your research to make findings accessible to a wide audience that includes both scientists and non-scientists. The Author Summary should immediately follow the Abstract in your revised manuscript. This text is subject to editorial change and should be distinct from the scientific abstract.

In addition to these revisions, you may need to complete some formatting changes, which you will receive in a follow up email. A member of our team will be in touch with a set of requests shortly. If you do not receive a separate email within a few days, please assume that checks have been completed, and no additional changes are required.

We expect to receive your revised manuscript within 1 week. Please email us (plosmedicine@plos.org) if you have any questions or concerns.

We ask every co-author listed on the manuscript to fill in a contributing author statement. If any of the co-authors have not filled in the statement, we will remind them to do so when the paper is revised. If all statements are not completed in a timely fashion this could hold up the re-review process. Should there be a problem getting one of your co-authors to fill in a statement we will be in contact. YOU MUST NOT ADD OR REMOVE AUTHORS UNLESS YOU HAVE ALERTED THE EDITOR HANDLING THE MANUSCRIPT TO THE CHANGE AND THEY SPECIFICALLY HAVE AGREED TO IT.

Please ensure that the paper adheres to the PLOS Data Availability Policy (see http://journals.plos.org/plosmedicine/s/data-availability), which requires that all data underlying the study's findings be provided in a repository or as Supporting Information. For data residing with a third party, authors are required to provide instructions with contact information for obtaining the data. PLOS journals do not allow statements supported by "data not shown" or "unpublished results." For such statements, authors must provide supporting data or cite public sources that include it.

To enhance the reproducibility of your results, we recommend that you deposit your laboratory protocols in protocols.io, where a protocol can be assigned its own identifier (DOI) such that it can be cited independently in the future. Additionally, PLOS ONE offers an option to publish peer-reviewed clinical study protocols. Read more information on sharing protocols at https://plos.org/protocols?utm_medium=editorial-email&utm_source=authorletters&utm_campaign=protocols

Please review your reference list to ensure that it is complete and correct. If you have cited papers that have been retracted, please include the rationale for doing so in the manuscript text, or remove these references and replace them with relevant current references. Any changes to the reference list should be mentioned in the rebuttal letter that accompanies your revised manuscript.

Please note, when your manuscript is accepted, an uncorrected proof of your manuscript will be published online ahead of the final version, unless you've already opted out via the online submission form. If, for any reason, you do not want an earlier version of your manuscript published online or are unsure if you have already indicated as such, please let the journal staff know immediately at plosmedicine@plos.org.

If you have any questions in the meantime, please contact me or the journal staff on plosmedicine@plos.org.

We look forward to receiving the revised manuscript by Jun 26 2026 11:59PM.

Sincerely,

Katarzyna Starowicz, PhD

Associate Editor

PLOS Medicine

plosmedicine.org

------------------------------------------------------------

Comments from Reviewers:

RESPONSE ACCEPTABLE: I feel that the authors have done a reasonable job of addressing the low number of Grade 3 RILI events, which necessitated pooling Grade 2 and Grade 3 RILI. The authors' one-way sensitivity analyses did not take into account the correlation of cost and disutility, which increase together with greater disease severity, but I would imagine this would only strengthen the study's conclusion that the therapy is cost-effective.

RESPONSE ACCEPTABLE: Regarding the utility of lower-grade adverse events, I did not find the authors' initial response compelling (that other studies have ignored these, and they can be ignored for patients with severe illness). However, the additional analyses presented, benchmarked on Grade 1-2 AE disutility from literature, do appear convincing, as evidence that the cost-effectiveness conclusion is robust to AE (dis)utility. As a minor point, it might be useful to highlight in the Limitations that there was no direct quality-of-life measurement in the study, and therefore the AE disutility was benchmarked from literature not necessarily generalizable to the study population. However, they authors could point out that this is mitigated by the sensitivity analyses.

NEEDS REVISION: Regarding the WTP threshold, the World Health Organization has explicitly withdrawn its guidelines on using 3x the GDP. This was not an appropriate choice for benchmarking the China-specific WTP threshold and should be revised using modern WTP literature (e.g., benchmarked on the marginal productivity of the healthcare system or individuals' WTP for health as a proportion of income).

Any attachments provided with reviews can be seen via the following link:

[LINK]

# Author comment: Author response to Decision Letter 4

**DOI:** 10.1371/journal.pmed.1004817.r007

23 Jun 2026

Attachment Submitted filename: Response_to_reviewers_auresp_4.docx

# Editor report: Decision Letter 4

**DOI:** 10.1371/journal.pmed.1004817.r008

**Authors:** Katarzyna Starowicz

7 Jul 2026

Dear Dr Chen,

On behalf of my colleagues and the Academic Editor, Aadel A Chaudhuri, I am pleased to inform you that we have agreed to publish your manuscript "Cost-Effectiveness of Pirfenidone for Cancer Patients with Grade 2 and Grade 3 Radiation-Induced Lung Injury: A Modelling Study" (PMEDICINE-D-25-03853R4) in PLOS Medicine.

Before your manuscript can be formally accepted you will need to complete some formatting and text changes, which you will receive in a follow up email. Please be aware that it may take several days for you to receive this email; during this time no action is required by you. Once you have received these formatting requests, please note that your manuscript will not be scheduled for publication until you have made the required changes.

Please after receiving the email from our Production Department contact me (kstarowicz@plos.org) to discuss proposed change of the tittle.

In the meantime, please log into Editorial Manager at http://www.editorialmanager.com/pmedicine/, click the "Update My Information" link at the top of the page, and update your user information to ensure an efficient production process.

PRESS

We frequently collaborate with press offices. If your institution or institutions have a press office, please notify them about your upcoming paper at this point, to enable them to help maximise its impact. If the press office is planning to promote your findings, we would be grateful if they could coordinate with medicinepress@plos.org. If you have not yet opted out of the early version process, we ask that you notify us immediately of any press plans so that we may do so on your behalf.

We also ask that you take this opportunity to read our Embargo Policy regarding the discussion, promotion and media coverage of work that is yet to be published by PLOS. As your manuscript is not yet published, it is bound by the conditions of our Embargo Policy. Please be aware that this policy is in place both to ensure that any press coverage of your article is fully substantiated and to provide a direct link between such coverage and the published work. For full details of our Embargo Policy, please visit http://www.plos.org/about/media-inquiries/embargo-policy/.

To enhance the reproducibility of your results, we recommend that you deposit your laboratory protocols in protocols.io, where a protocol can be assigned its own identifier (DOI) such that it can be cited independently in the future. Additionally, PLOS ONE offers an option to publish peer-reviewed clinical study protocols. Read more information on sharing protocols at https://plos.org/protocols?utm_medium=editorial-email&utm_source=authorletters&utm_campaign=protocols

Thank you again for submitting to PLOS Medicine. We look forward to publishing your paper.

Sincerely,

Katarzyna Starowicz, PhD

Associate Editor

PLOS Medicine
